# Comprehensive Model for Epidermal Growth Factor Receptor Ligand Binding Involving Conformational States of the Extracellular and the Kinase Domains

**DOI:** 10.3389/fcell.2020.00776

**Published:** 2020-08-11

**Authors:** Tímea Hajdu, Tímea Váradi, István Rebenku, Tamás Kovács, János Szöllösi, Peter Nagy

**Affiliations:** ^1^Department of Biophysics and Cell Biology, Faculty of Medicine, University of Debrecen, Debrecen, Hungary; ^2^Doctoral School of Molecular Medicine, Faculty of Medicine, University of Debrecen, Debrecen, Hungary; ^3^MTA-DE Cell Biology and Signaling Research Group, Faculty of Medicine, University of Debrecen, Debrecen, Hungary

**Keywords:** EGF receptor, ligand binding, cooperativity, dimerization, kinase domain

## Abstract

The epidermal growth factor (EGF) receptor (EGFR) undergoes ligand-dependent dimerization to initiate transmembrane signaling. Although crystallographic structures of the extracellular and kinase domains are available, ligand binding has not been quantitatively analyzed taking the influence of both domains into account. Here, we developed a model explicitly accounting for conformational changes of the kinase and extracellular domains, their dimerizations and ligand binding to monomeric and dimeric receptor species. The model was fitted to ligand binding data of suspended cells expressing receptors with active or inactive kinase conformations. Receptor dimers with inactive, symmetric configuration of the kinase domains exhibit positive cooperativity and very weak binding affinity for the first ligand, whereas dimers with active, asymmetric kinase dimers are characterized by negative cooperativity and subnanomolar binding affinity for the first ligand. The homodimerization propensity of EGFR monomers with active kinase domains is ∼100-times higher than that of dimers with inactive kinase domains. Despite this fact, constitutive, ligand-independent dimers are mainly generated from monomers with inactive kinase domains due to the excess of such monomers in the membrane. The experimental finding of increased positive cooperativity at high expression levels of EGFR was recapitulated by the model. Quantitative prediction of ligand binding to different receptor species revealed that EGF binds to receptor monomers and dimers in an expression-level dependent manner without significant recruitment of monomers to dimers upon EGF stimulation below the phase transition temperature of the membrane. Results of the fitting offer unique insight into the workings of the EGFR.

## Introduction

The ErbB/HER family of receptor tyrosine kinases (RTKs) comprises four transmembrane receptors activated by a large number and variety of peptide growth factors (Yarden and Sliwkowski, 2001). ErbB1, also known as the epidermal growth factor (EGF) receptor (EGFR), is activated by ligand binding followed by tyrosine phosphorylation of its C-terminal domain and recruitment of SH2 and PTB domain-containing proteins (Wagner et al., 2013). While EGFR is one of the most studied transmembrane receptors, perplexing questions remain about the fine details of its activation (Jovin, 2014). Although larger clusters of EGFR has also been described (Szabó et al., 2008; Needham et al., 2016), dimerization is believed to be the key in regulating receptor activation, a notion strongly supported by crystallographic structures of the extracellular and kinase domains of the receptor (Ogiso et al., 2002; Zhang et al., 2006). The tethered or closed structure of the extracellular domain (ECD) undergoes a rearrangement to an extended conformation forming back-to-back, ligand-bound dimers stabilized by the exposed dimerization arm (Lemmon, 2009; Ziomkiewicz et al., 2013). Besides this dimeric species, ligand-bound head-to-head dimers (Garrett et al., 2002) and ligand-free dimeric species, including side-to-side dimers have also been reported (Moriki et al., 2001; Zanetti-Domingues et al., 2018). Dimerization is not only mediated by the ECD, but also by the transmembrane (TMD) and intracellular domains under the influence of the lipid environment of the plasma membrane (Kovacs et al., 2015; Kovács et al., 2016; Zákány et al., 2020). The TMD of EGFR contains two dimerization motifs, with the N- and C-terminal ones suggested to stabilize active and inactive dimers, respectively (Fleishman et al., 2002). The kinase domain (KD) is also capable of forming at least two different dimeric species. The kinase is activated in an asymmetric dimer by a mechanism recapitulating how cyclins activate cyclin-dependent kinases, while the symmetric dimer, although harboring KDs in their active conformation, is unlikely to be capable of signal transduction (Landau et al., 2004; Zhang et al., 2006; Jura et al., 2009; Kovacs et al., 2015). Most tyrosine kinase inhibitors bind to the ATP-binding pocket of the kinase. Type I inhibitors, including erlotinib, stabilize the active conformation of the kinase, while type II inhibitors, e.g., lapatinib, stabilize its inactive structure (Stamos et al., 2002; Wood et al., 2004; Zhang et al., 2009). Interactions of the juxtamembrane segment with negatively charged lipids in the inner leaflet of the plasma membrane prevent formation of active kinase dimers, while the juxtamembrane segment also forms activating interactions with the KD (Jura et al., 2009). Although the large variety of dimeric structures is complicated, a scheme seems to emerge in which the asymmetric, active kinase dimer, the TMD dimer stabilized by its N-terminal dimerization motif and the liganded, back-to-back ECD dimer mutually favor each other, since this conformation of the ECD holds the C-terminal dimerization motifs of the TMD apart and the asymmetric KD dimer pulls the N-terminal dimerization motifs of the TMD together (Diwanji et al., 2019). The finding of coupling between the KD activated by the L858R mutation and the unliganded, dimerization competent ECD also supports the previous model (Valley et al., 2015). Interactions between different parts of inactive receptors is more controversial. While coupling of the symmetric kinase dimer to the TMD dimer stabilized by its C-terminal dimerization motif is widely accepted, conformational coupling of this symmetric kinase dimer and the transmembrane domains to the ECD is debated. While some evidence suggests that the liganded ECD can couple to both the symmetric and asymmetric kinase dimers (Mi et al., 2011), a strict linkage between symmetric kinase dimers and the closed conformation of the ECD has also been put forward (Bessman et al., 2014; Macdonald-Obermann and Pike, 2018). In addition, the ECD of inactive receptors has been suggested to form alternative structures as well, like side-to-side dimers (Moriki et al., 2001) or head-to-head oligomers (Zanetti-Domingues et al., 2018).

Although significant progress has been made in revealing conformational transitions of isolated receptor parts upon activation, correlations between these receptor states in the full-length protein have not been described due to the flexibility of the juxtamembrane domains and the difficulty of studying structurally homogenous EGFR populations (Mi et al., 2011; Diwanji et al., 2019). These correlations implicitly determine the concentration dependence of ligand binding. Oligomerization linkage takes place when a liganded monomer undergoes dimerization differently than its unliganded counterpart revealing ligand-induced conformational transitions. Throughout the paper this phenomenon will be briefly referred to as linkage. When ligands bind cooperatively to a dimer, the dissociation constants for consecutive ligand bindings are not identical implying conformational changes taking place in the dimer. Although this phenomenon has been named homotropic ligand linkage, the term cooperativity has been so widely accepted that it will be used for referring to this phenomenon (Wyman and Gill, 1990; Pike, 2012). Analysis of equilibrium ligand binding is often carried out by fitting the Hill equation to data providing the Hill cooperativity coefficient. Although a Hill coefficient different from one is interpreted as a sign of cooperativity, we are going to refer to this phenomenon as apparent or phenomenological cooperativity since linkage and molecular cooperativity collectively determine the Hill coefficient.

Quantitative EGF binding assays may produce concave-up Scatchard plots, which were attributed to two independent binding sites for decades (Defize et al., 1988; Bellot et al., 1990; Özcan et al., 2006). However, negative cooperativity has been invoked to account for the apparent heterogeneity of EGF binding sites (Macdonald and Pike, 2008). This phenomenon requires that binding of the first ligand to an EGFR dimer decreases the ligand binding affinity of the other subunit by inducing an asymmetric ECD dimer with a constrained ligand binding site on the unoccupied receptor. While the drosophila EGFR, whose isolated ECD retains negative cooperativity, has indeed been reported to form such asymmetric dimers (Alvarado et al., 2010), its human counterpart only exhibits negative cooperativity when ligands bind to full-length receptors in cells. Although low affinity ligands bound to human EGFR stabilize an asymmetric ECD dimer (Freed et al., 2017), such an asymmetry has not been observed when high affinity ligands, like EGF, are complexed with the receptor (Martin-Fernandez, 2012). These facts suggest that other receptor regions, conformations and/or unknown cellular components must be involved in the regulation of ligand affinity of EGFR in the cellular environment. Although negative cooperativity became the dogma in EGF binding studies, several investigators reported positive cooperative ligand binding to EGFR (Sherrill and Kyte, 1996; Lemmon et al., 1997; Teramura et al., 2006; Chung et al., 2010) with a recent report linking positive cooperativity to receptor dimers with symmetric kinase dimers, but without quantitative analysis based on molecular structures (Macdonald-Obermann and Pike, 2018). The present study was undertaken to generate a structure-based model of EGF binding involving conformational transitions of the extracellular and intracellular domains. The obtained equations not only fit the experimental data well, but are in accordance with most published structural results and can resolve the aforementioned contradictions regarding cooperativity of ligand binding.

## Materials and Methods

### Cells

The CHO-K1 cell line (ATCC CCL-61) was obtained from the American Type Culture Collection (Manassas, VA, United States). It’s subline, F1-4, stably expresses EGFR-GFP (Brock et al., 1999). F1-4 cells were maintained at 37°C and 5% CO_2_ in Dulbecco’s Modified Eagle’s medium (DMEM) supplemented with 10% fetal calf serum and 50 μg/ml gentamicin. The number of passages of cells used for the experiments never exceeded 15. The expression level of EGFR was determined by flow cytometry using Qifikit (Agilent Technologies, Santa Clara, CA, United States). For stimulation of EGFR, cells were cultured in DMEM containing 0.1% fetal calf serum and 50 μg/ml gentamicin for 12 h followed by incubation with 130 nM EGF (R&D Systems, Minneapolis, MN, United States) in Hank’s balanced salt solution at 37°C or 4°C for 15 min or 1 h.

### Reagents

For microscopic experiments cells were cultured on μ-slide 8-well chambered coverglass (Ibidi, Martinsried, Germany). For flow cytometry cells were harvested by trypsinization. Human EGF (cat. no: 236-EG) was purchased from R&D Systems (Minneapolis, MN, United States). Tetramethylrhodamine-labeled EGF (cat. no: E3481) was purchased from Thermo Fisher Scientific (Waltham, MA, United States). EGF-stimulated tyrosine phosphorylation was visualized by labeling with an antibody against phosphotyrosine (PY99, cat. no: sc-7020; Santa Cruz, Dallas, TX, United States) followed by secondary staining with AlexaFluor647-conjugated goat-anti-mouse IgG (cat. no: A-21235; Thermo Fisher Scientific). Tunicamycin (cat. no: T7765), latrunculin-B (cat. no: L5288) and jasplakinolide (Cat. no: J4580) were purchased from Sigma-Aldrich (St. Louis, MO, United States). Tetramethylrhodamine B isothiocyanate-labeled phalloidin (TRITC-phalloidin, cat. no: P1951) and 4′,6-diamidino-2′-phenylindole (DAPI, cat. no: D9542) were obtained from Sigma Aldrich. Lapatinib (cat. no: S2111) and erlotinib (cat. no: S7786) were purchased from Selleckchem (Houston, TX, United States).

### Labeling of Cells With Fluorescent EGF

Tetramethylrhodamine-conjugated EGF (TAMRA-EGF) was reconstituted at a concentration of 10 μM. A two-fold dilution series of TAMRA-EGF was prepared with each vial containing 500 μl TAMRA-EGF dissolved in phosphate-buffered saline supplemented with 1% (w/v) BSA. The solutions were kept on ice before adding 20 μl of a cold cell suspension containing 100,000 cells. Cells were incubated in the presence of TAMRA-EGF for 1 h on ice with shaking. The fluorescence intensity of the samples was measured on a FACS Aria III flow cytometer (BD Biosciences, San Jose, CA, United States) without washing to prevent the dissociation of fluorescent EGF. TAMRA was excited at 561 nm, and its emission was detected through a 595 nm band-pass filter. Analysis of flow cytometric data was carried out with FCS Express (*Denovo* Software, Thornhill, ON, Canada). Mean fluorescence intensities were background-corrected followed by fitting the Hill equation to the binding curves:

(1) $$I=I_{\min}+\frac{I_{\max}-I_{\min}}{1+10^{n\,\left(\log K_{d}\,-\,\log c\right)}}$$

where *I* is the intensity of the sample labeled with concentration *c* of fluorescent EGF, *I*_*min*_ and *I*_*max*_ are the minimal and maximal intensities, respectively. *K*_*d*_ and *n* are the dissociation constant and the Hill coefficient, respectively. The same datasets were also used for fitting of the model described in the present manuscript.

### Treatment of Cells With Kinase Inhibitors

In order to measure the effect of kinase inhibitors on EGF-induced phosphorylation cells were grown on μ-slide 8-well chambered coverglass and were serum-starved for 12 h. Then, they were exposed to erlotinib or lapatinib at a concentration of 5 μM at 37°C for 1 h followed by stimulation with EGF at a concentration of 130 nM for 15 min at 37°C. Cells were fixed in 3.7% (v/v) formaldehyde, permeabilized with a solution of 0.1% (v/v) Triton-X100 containing 1% (w/v) BSA in PBS followed by secondary staining with a pan-phosphotyrosine antibody (PY99) and AlexaFluor647 goat anti-mouse IgG. Samples were observed using a Zeiss LSM880 confocal microscope with a C-Apochromat 40 × (N.A. = 1.2) water immersion objective. GFP and AlexaFluor647 were excited at 488 and 633 nm, respectively. Emissions of GFP and AlexaFluor647 were measured in the wavelength range of 490–570 nm and 635–755 nm, respectively. In order to measure the effect of kinase inhibitors on EGF binding trypsinized cells were treated with erlotinib or lapatinib at a concentration of 5 μM at 37°C for 1 h followed by labeling with TAMRA-EGF as described previously.

### Inhibition of Glycosylation of EGFR

F1-4 cells were treated with tunicamycin at a concentration of 1 μg/ml for 24 h followed by trypsinization and labeling with TAMRA-EGF as described above. In order to show the effect of deglycosylation on the molecular weight of EGFR control and tunicamycin-treated cells were lysed and scraped followed by running the lysate in a 7% SDS-PAGE gel. Proteins were transferred to PVDF membranes, and the blots were incubated with an antibody against EGFR (anti-EGFR, clone F4, Thermo Fisher Scientific; Cat. no: MA1-24226) at 4°C overnight followed by labeling with anti-mouse IgG-peroxidase (Sigma-Aldrich; cat no: AP124P) for detection with a chemiluminescence kit (Thermo Fisher Scientific; cat no: 34577, 34095).

### Inhibition or Promotion of Actin Polymerization

Cells were treated with latrunculin-B at a concentration of 2 μM at 37°C for 10 min or by 1 μM jasplakinolide at 37°C for 30 min followed by labeling with a concentration series of TAMRA-EGF as described above. In order to visualize the effect of latrunculin-B or jasplakinolide on microfilaments, control and treated cells were permeabilized with acetone followed by staining with 4 μg/ml TRITC-phalloidin and 10 μg/ml DAPI. Samples were observed with a Zeiss LSM880 confocal microscope using a C-Apochromat 40 × (N.A. = 1.2) water immersion objective. DAPI and TRITC were excited at 405 nm and 543 nm, respectively. The emissions of DAPI and TRITC were detected in the wavelength range of 410–482 nm and 557–655 nm, respectively.

### Homo-FRET Measurements

F1-4 cells were seeded at a density of 5 × 10^4^ cells/well on μ-slide 8-well chambered coverglass, and serum-starved overnight. After washing with Hank’s balanced salt solution, cells were treated with erlotinib or lapatinib at a concentration of 5 μM at 37°C for 1 h followed by stimulation with EGF at a concentration of 130 nM for 15 min at 37°C or 4°C. Imaging was carried out with a Zeiss LSM880 confocal microscope without washing or diluting the samples. Samples were examined with a C-Apochromat 40 × (N.A. = 1.2) water immersion objective. Stepwise bleaching of GFP was accomplished by a 405-nm laser line till the GFP signal completely faded. Fluorescence was observed in two tracks detecting the fluorescence emission polarized parallel (*I*_∥_) and perpendicular (*I*_⊥_) to the excitation beam by a Quasar detector in the L-format arrangement. Intensities were summed in membrane pixels, identified by manually seeded watershed segmentation, after subtracting background fluorescence determined in a cell-free area of the image. Intensities were corrected for high numerical aperture detection followed by calculating anisotropy (*r*) as follows (Jovin, 1979):

(2) $$r=\frac{I_{∥}-G\,\,I_{⊥}}{I_{∥}+2\,\,G\,\,I_{⊥}}$$

The G factor, characterizing the sensitivity of the detection system to the parallel and perpendicular intensity components, was determined by imaging a GFP solution using microscope settings identical to the ones used for the cells.

Homo-FRET implemented in microscopy or flow cytometry has been used previously to analyze receptor clustering (Lidke et al., 2003; Yeow and Clayton, 2007; Szabó et al., 2008; Hofman et al., 2010). In order to estimate homoclustering of EGFR, the anisotropy, calculated from membrane pixels, of each image in the bleaching sequence was plotted against the residual fractional intensity of GFP, and an equation describing the anisotropy of a mixture of monomers and homoclustersed receptors was fitted to the measured data points. We assumed that a fraction of proteins (*mon*) is unclustered, whereas the rest of them form clusters consisting of *N* fluorophores. The equation describing the anisotropy of such a population of fluorophores (*r*_*s,N*_) was fitted to the measured data (Szabó et al., 2008):

(3) $$\begin{array}{l} r_{s,N}=\frac{\left(1-mon\right)}{Ns}\overset{N}{\underset{k=0}{\sum }}[\left(\frac{N}{k}\right)s^{k}{\left(1-s\right)}^{N-k}k\\ \;\left(r_{1}\frac{1+d^{6}}{1+kd^{6}}+r_{FRET}\frac{\left(k-1\right)d^{6}}{1+kd^{6}}\right)]+mon\cdot r_{1} \end{array}$$

where *s* is the fraction of unbleached fluorophores, *r*_1_ and *r*_*FRET*_ are the anisotropies of an isolated fluorophore and a fluorophore excited by homo-FRET, respectively, and *d* is the reciprocal of the distance between the fluorophores in the clusters normalized to *R*_0_. *r*_1_ was assumed to be 0.34 (Volkmer et al., 2000), while the anisotropy of fluorophores excited by homo-FRET (*r*_*FRET*_) was assumed to be zero (Runnels and Scarlata, 1995). The distance between two fluorophores in a cluster was assumed to be equal to the Förster distance for a GFP-GFP homo-FRET pair (4.8 nm), which is in the same order of magnitude as the size of a GFP barrel (Yang et al., 1996; Kremers and Goedhart, 2009). Fitting provided the number of proteins in a homocluster (*N*, cluster size) and the fraction of monomeric receptors (*mon*). The reliability of the estimation at the given biological variability and measurement error was determined by Monte Carlo simulation. Five hundred anisotropy vs. fractional residual intensity curves were generated using the mean and the standard deviation of the anisotropies. All curves were fitted generating 500 estimations for the cluster size and monomeric fraction. The histograms of these values and their 95% confidence intervals provided an estimation for the reliability of the fitting (Szabó et al., 2008).

## Results

### Equilibrium Binding of EGF to Cells With or Without Kinase Inhibitor Treatment

**FIGURE 1:**
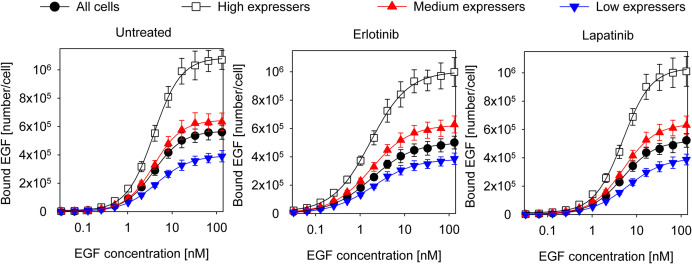
Equilibrium EGF binding and their fits according to the Hill equation. Control F1-4 cells and those treated with 5 μM erlotinib or lapatinib for 1 h were incubated with a 2-fold concentration series of EGF for an hour on ice. Cells pretreated with the kinase inhibitors were incubated with EGF in the continued presence of the inhibitors. The fluorescence intensity of cells was measured by flow cytometry without washing or diluting the samples. The whole cell population and three subpopulations identified according to their different EGFR-GFP expressions were separately analyzed. The symbols represent the mean of three independent measurements with the error bars showing the standard error of the mean. The lines show fits of the Hill equation to the experimental data. Results of the fit are presented in Table 1.

Coupling between the conformations of the intra- and extracellular parts of EGFR are expected to affect the apparent cooperativity and affinity of EGF binding, which can be estimated by analyzing the dependence of these parameters on the expression level of the receptor. In order to look at cell populations having different EGFR expression levels without disturbing the homeostasis of cells significantly, we used F1-4 cells, a CHO subline stably transfected with EGFR-GFP. Flow cytometric gating on the GFP fluorescence makes selection of subpopulations with different expression levels of EGFR possible (Supplementary Figure 1). Although quantitative imaging of fluorescent EGF bound to attached cells is possible (Teramura et al., 2006; Chung et al., 2010), the statistical reliability of such measurements, especially when restricted to a subpopulation with a certain receptor expression level, is poor due to the low number of analyzed cells. We expected that such noisy data would have prevented successful fitting of a complex model. In order for our flow cytometric approach to be relevant for the physiological state of EGFR, we showed that trypsinization, used for detaching cells from culture flasks, had negligible effect on the expression level and molecular weight of EGFR on the cell surface (Supplementary Figure 2). In spite of these findings, it must be pointed out that all binding measurements were carried out with suspended cells. We also established that the EGF binding characteristics of the EGFR-GFP fusion construct are identical to those of native EGFR (Supplementary Figure 3). Fitting of the Hill equation to the equilibrium EGF binding data in F1-4 cells revealed nanomolar apparent *K*_*d*_ and positive apparent cooperativity, which increased as a function of receptor expression (Figure 1 and Table 1). Throughout the analyses presented in the manuscript ligand depletion was assumed to be negligible. The validity of this assumption is shown in the Supplementary Information. In order to test explicitly the influence of different states of the KD on EGF binding, inhibitors stabilizing the kinase in the active and inactive conformations were used. Their effect on EGF-induced tyrosine phosphorylation is shown in Supplementary Figure 4. Erlotinib, binding and stabilizing the active conformation of the kinase, increased the affinity of the receptor for EGF and reduced the Hill coefficient to a range characteristic of negative apparent cooperativity in all cell populations except for those exhibiting the highest expression. Cells treated with lapatinib, an inhibitor stabilizing the inactive conformation of the KD, were characterized by positive cooperative EGF binding with reduced apparent affinity (Figure 1 and Table 1). The results revealed that EGFR exhibits an expression level-dependent tendency for positive apparent cooperativity in ligand binding, which is also influenced by the conformation of the KD.

**TABLE 1:** EGF binding fitted according to the Hill equation.

	Low expresser	Medium expresser	High expresser	All cells
Untreated	*n* = 1.14 ± 0.004	*n* = 1.33 ± 0.008	*n* = 1.41 ± 0.01	*n* = 1.26 ± 0.006
	*K*_*d*_ = 4.4 ± 0.005 nM *N* = 390,000	*K*_*d*_ = 3.8 ± 0.01 nM *N* = 630,000	*K*_*d*_ = 3.8 ± 0.01 nM *N* = 1,070,000	*K*_*d*_ = 3.9 ± 0.007 nM *N* = 560,000
Erlotinib	*n* = 0.92 ± 0.02	*n* = 0.99 ± 0.01	*n* = 1.03 ± 0.01	*n* = 0.95 ± 0.01
	*K*_*d*_ = 2 ± 0.02 nM *N* = 390,000	*K*_*d*_ = 1.8 ± 0.01 nM *N* = 630,000	*K*_*d*_ = 1.7 ± 0.02 nM *N* = 1,000,000	*K*_*d*_ = 1.9 ± 0.01 nM *N* = 500,000
Lapatinib	*n* = 1.04 ± 0.01	*n* = 1.21 ± 0.01	*n* = 1.36 ± 0.01	*n* = 1.2 ± 0.01
	*K*_*d*_ = 6.3 ± 0.01 nM *N* = 390,000	*K*_*d*_ = 5.1 ± 0.01 nM *N* = 630,000	*K*_*d*_ = 4.9 ± 0.02 nM *N* = 1,010,000	*K*_*d*_ = 4.9 ± 0.02 nM *N* = 520,000

*Equilibrium EGF binding data was fitted with the Hill equation. The apparent dissociation constant (*K*_*d*_) and the Hill coefficient (*n*, ± their standard deviations) are displayed for the control sample and cells treated with the kinase inhibitors. The standard deviations were estimated by multiplying the inverse of the Hessian with the standard deviation of the fitting error. The expression level of EGFR of the whole cell population was measured by flow cytometric calibration. EGFR expressions of the low, medium and high expresser subpopulations were determined by assuming that the maximum intensity of EGF fluorescence, determined from the Hill fits, is proportional to the receptor expression level. The number of receptors/cell, designated by N, is shown at the bottom of each cell. The experimental data and the fits are shown in Figure 1.*

### Development of a Model Involving Conformational States of the Ligand-Binding and Kinase Domains

Given the results obtained with cells treated with the two kinase inhibitors, establishing that the KD exerts significant effects on the characteristics of EGF binding, and structural evidence pointing at dimerization of the KD, we developed a molecular model of EGF binding based on the following assumptions. 1. The closed conformation of the ECD is in equilibrium with its extended structure (Klein et al., 2004). Although the ligandless ECD has been found to be dynamic, assuming other conformations as well (Kaplan et al., 2016), they were not included in the model in order to limit the number of free parameters. 2. Receptor dimerization only takes place with receptors whose ECD is in the extended conformation (Garrett et al., 2002; Ogiso et al., 2002). 3. The KD is assumed to adopt either an inactive or active conformation in receptor monomers. Although other conformations of the KD have also been reported (Shan et al., 2012), the number of molecular species had to be minimized so that the model remains tractable. Therefore, these intermediate conformations were excluded from the model. 4. The conformations of the intra- and extracellular domains are uncoupled from each other in monomeric receptors. 5. The extended conformation of the ECD dimers can couple with both symmetric and asymmetric KD dimers, in accordance with high resolution electron microscopic evidence (Mi et al., 2011). While assignment of symmetric and asymmetric configurations to the two different kinds of kinase dimers in the model is not based on firm structural evidence, the assumption of two different receptor dimerization pathways beginning from receptors harboring inactive and active KDs was absolutely required for successful fitting of the data. With this limitation in mind, we still refer to these kinase dimers as symmetric and asymmetric. The structure of kinase domain dimers assigned to these two different dimerization pathways will be further addressed in the Discussion. 6. Dimers with symmetric and asymmetric KD dimers are characterized by different affinities for EGF. Incorporation of this assumption was required so that the model could reproduce the significant dependence of apparent cooperativity on expression levels and on the presence of the two kinase inhibitors. A possible explanation for the validity of this assumption is provided in the Discussion. 7. Ligand binds only to the extended conformation of the ECD. Models describing EGF binding including or neglecting ligand binding to the closed conformation of the ECD have both been published (Klein et al., 2004; Macdonald and Pike, 2008). Since the affinity of a dimer harboring symmetric kinase dimers for binding of the first ligand turned out to be low, incorporation of another low affinity binding site, the closed ECD, would have made the model less reliable. Besides this model, several alternative ones have been tested. Receptor dimerization beginning from a monomer with extended ECD and an asymmetric kinase dimer was included in all of them. Without inclusion of another dimerization pathway the result of the fitting was poor. If the second dimerization pathway began from either of the monomers with closed ECD (“CA” or “CI”), fittings were also of poor quality. The approach taken to construct the model is a compromise between an even more detailed, but mathematically intractable model taking every possible state and transition into consideration and a biologically unrealistic, simplistic model, which is easy to handle. Such a golden mean has been suggested to result in realistic and experimentally verifiable models (Yates et al., 2001).

**FIGURE 2:**
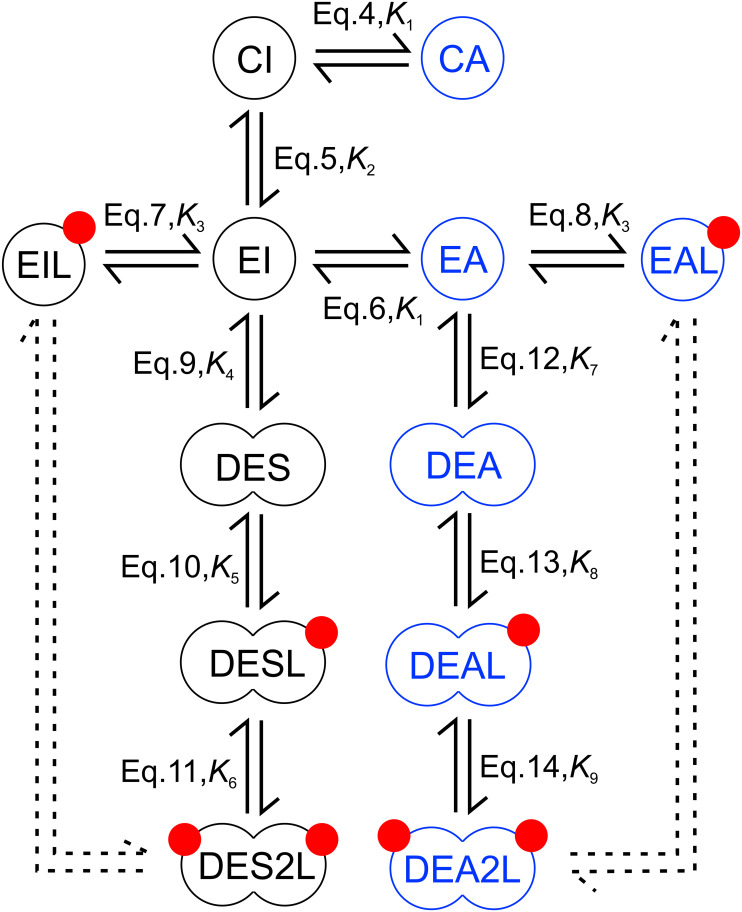
Model for EGF binding involving conformations of the ligand binding and kinase domains. The extracellular part of the receptor is present in either a closed (C) or extended (E) conformation with both of these states coupled to either an active (A) or inactive (I) kinase domain generating four possible monomers (CI, CA – closed extracellular domain with inactive and active kinase domain, respectively; EI, EA – extended extracellular domain with inactive and active kinase domain, respectively). Receptors whose extracellular domain is in the extended conformation can bind EGF (red circles). Liganded species are designated by an ‘L’ at the end of their names. Receptors with extended extracellular domains can form dimers designated by a ‘D’ at the beginning of their names. Monomeric and dimeric species, whose kinase domain is in an active or inactive conformation, are shown in blue and black, respectively. Three different kinds of dimers with inactive, symmetric kinase dimers are present according to their ligand binding state (DES, DESL, DES2L – dimers with inactive, symmetric kinase dimers with 0, 1 and 2 bound EGF, respectively). The same designation principle applies to dimeric species with active, asymmetric kinase dimers (DEA, DEAL, DEA2L). The double arrows (continuous lines) show those transitions, which are explicitly included in describing the equilibrium. The descriptions beside the arrows show the equation and constant describing the equilibrium corresponding to the arrow. The equilibrium of all other conformational changes, ligand binding and dimerization events not explicitly shown in the figure are fully described by the equation set discussed in the main text since the equilibrium constants determine the standard Gibbs energy change of a reaction and consequently the ratio of the equilibrium concentrations of any pairwise selection of species. Although the dashed double arrow labeling the dimerization of liganded monomers is not explicitly included in the model, it is required for determining linkage in the system.

The model, presented in Figure 2, involves twelve molecular species, whose equilibrium state is described by eleven equations and nine constants. The following equations characterize the conformational transitions and ligand binding of monomeric species:

(4) $$\left[C\,I\right]=K_{1}\,\left[C\,A\right]$$

(5) $$\left[C\,I\right]=K_{2}\,\left[E\,I\right]$$

(6) $$\left[E\,I\right]=K_{1}\,\left[E\,A\right]$$

(7) $$\left[E\,I\right]\,\left[E\,G\,F\right]=K_{3}\,\left[E\,I\,L\right]$$

(8) $$\left[E\,A\right]\,\left[E\,G\,F\right]=K_{3}\,\left[E\,A\,L\right]$$

where *K*_1_ and *K*_2_ are the equilibrium constants for the conformational transitions of the KD and the ECD, respectively, and *K*_3_ is the dissociation constant of EGF binding to a monomeric receptor having an extended ECD. The abbreviations of molecular species are defined in the legend to Figure 2. The following equations describe the dimerization of monomers leading to the formation of dimers with symmetric kinase dimers:

(9) $$\left[E\,I\right]\,\left[E\,I\right]=K_{4}\,\left[D\,E\,S\right]$$

(10) $$\left[D\,E\,S\right]\,\left[E\,G\,F\right]=K_{5}\,\left[D\,E\,S\,L\right]$$

(11) $$\left[D\,E\,S\,L\right]\,\left[E\,G\,F\right]=K_{6}\,\left[D\,E\,S\,2\,L\right]$$

where *K*_4_ is the dissociation constant of an unliganded dimer harboring extended ECD and symmetric KD dimer, and the dissociation constants of the first and second EGF binding to this dimer are denoted by *K*_5_ and *K*_6_, respectively. The dimerization and ligand binding pathway of species with asymmetric KD dimers are described by the equations below:

(12) $$\left[E\,A\right]\,\left[E\,A\right]=K_{7}\,\left[D\,E\,A\right]$$

(13) $$\left[D\,E\,A\right]\,\left[E\,G\,F\right]=K_{8}\,\left[D\,E\,A\,L\right]$$

(14) $$\left[D\,E\,A\,L\right]\,\left[E\,G\,F\right]=K_{9}\,\left[D\,E\,A\,2\,L\right]$$

where *K*_7_ is the dissociation constant of an unliganded dimer with extended ECD and asymmetric KD dimer, while *K*_8_ and *K*_9_ denote the dissociation constants of the first and second EGF binding to this dimer, respectively. The relationships describing the quantity of cell-bound EGF and the conservation of receptor number constitute the final two equations of the system:

(15) $$\begin{array}{l} \left[EGF_{bound}]=[EIL]+[EAL]+[DESL]+[DEAL\right]\\ \;+2([DES2L]+[DEA2L]) \end{array}$$

(16) $$\begin{array}{l} \left[R_{tot}]=[CI]+[CA]+[EI]+[EA]+[EIL]+[EAL\right]\\ \;+2([DES]+[DESL]+[DES2L]+[DEA]\\ \;+[DEAL]+[DEA2L]) \end{array}$$

where *R*_*tot*_ is the number of receptors/cell. The unit of ligand dissociation constants in the model is nM, whereas the unit of receptor dissociation constants is number of receptors/cell. Equations (4)-(16), constituting a quadratic equation set containing 13 unknowns (the concentration of the 12 molecular species and [*EGF*_*bound*_]), were solved with Mathematica (Wolfram Research, Champaign, IL). The set of roots in which all 13 concentrations were positive was selected as the meaningful solution, which is presented as a Matlab (Mathworks, Natick, MA) file in the Supplementary Information.

Control cells and the samples treated with erlotinib or lapatinib were divided into three subpopulations corresponding to low, medium and high expressers of EGFR. In this way there were four cell populations for each experimental condition (the three gated subpopulations and the whole cell population), altogether constituting 12 experimental conditions. The sum of squared deviations between the fitted equations and the experimental data was minimized by an algorithm, which was global in two respects: (1) All 12 data sets were fitted simultaneously with parameters *K*_2_-*K*_9_ shared between all of them, while *K*_1_ characterizing the conformational equilibrium between the active and inactive KDs was allowed to have three different values for the control, erlotinib- and lapatinib-treated samples. In this way, the 11 free parameters in the model were globally fitted to 156 data points from the 12 data sets. (2) The Global Search algorithm of Matlab was used for finding the global minimum of the norm. In order to define the confidence interval of the fitted parameters the optimization procedure was repeated 100-times.

#### Fitting of the Model to Equilibrium EGF Binding Data

**FIGURE 3:**
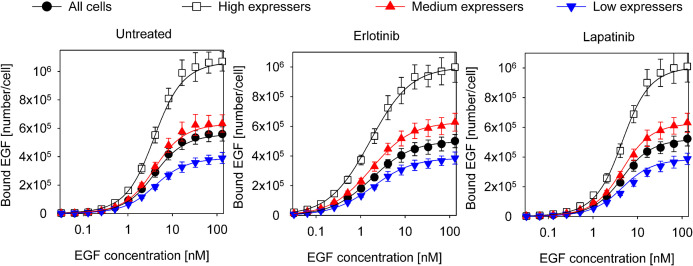
Fitting of equilibrium EGF binding according to the model involving structures of the extracellular and intracellular receptor parts. Experimental data, the same as shown in Figure 1, were fitted to the model introduced in Figure 2. The fitted model parameters are displayed in Table 2.

The data set analyzed previously with the Hill equation was fitted with the model described in the previous section (Figure 3, Table 2). The confidence intervals of the fitted parameters are shown in Supplementary Figure 5. The model reveals that the inactive conformation of the KD is favored under all experimental conditions in the absence of EGF, with the kinase inhibitors shifting this equilibrium in accordance with their presumed tendency to stabilize one of the kinase structures. In the absence of ligand, the ECD is preferentially in the closed conformation. Monomers with an inactive KD dimerize much less efficiently than monomers harboring an active KD. While the ligand binding affinity of EGFR monomers is in the nanomolar range, the KD exerts significant effects on the affinity and cooperativity of dimers. Dimeric structures with asymmetric kinase dimers exhibit subnanomolar affinity for the first ligand, but the second EGF binds with a ∼30-times lower affinity due to significant negative cooperativity. Dimers with symmetric kinase dimers have an extremely low affinity for the first ligand, but a subnanomolar binding constant is found for the second EGF. The model parameters revealed that the KD is strongly coupled to ligand binding suggesting that its involvement cannot be neglected in analyzing EGF binding data.

**TABLE 2:** Fitting of the proposed model to the equilibrium binding of EGF.

Parameter	Description	Scope of parameter	Value (95% CI)
*K*_1_	equilibrium constant for inactive and active KD (>1 favors the inactive conformation)	untreated	104 (67–155)
		erlotinib (active KD)	25 (17–38)
		lapatinib (inactive KD)	162 (101–243)
*K*_2_	equilibrium constant for the closed and extended conformations of the ECD (>1 favors the closed conformation)	global	2.9 (1.6–4.4)
*K*_3_	*K*_*d*_ of EGF binding to a monomer with extended ECD	global	1.3 nM (0.8–1.8 nM)
*K*_4_	dimerization of monomers with inactive KD	global	36,900 (15,600–68,700)
*K*_5_	*K*_*d*_ of binding of the first EGF to a dimer with inactive, symmetric kinase dimer	global	95 nM (69–99 nM)
*K*_6_	*K*_*d*_ of binding of the second EGF to a dimer with inactive, symmetric kinase dimer	global	0.45 nM (0.34–0.53 nM)
*K*_7_	dimerization of monomers with active KD	global	480 (86–2,400)
*K*_8_	*K*_*d*_ of binding of the first EGF to a dimer with active, asymmetric kinase dimer	global	0.1 nM (0.02–0.16 nM)
*K*_9_	*K*_*d*_ of binding of the second EGF to a dimer with active, asymmetric kinase dimer	global	2.96 nM (2.93–2.97 nM)

*The model involving conformational transitions of the kinase domain (KD) and extracellular domains (ECD) was fitted to equilibrium EGF binding. The measured data points and the fitted lines are shown in Figure 3. All twelve curves were fitted globally with the same value of parameters *K*_2_-*K*_9_, while three different values of *K*_1_ were used for the three experimental conditions (untreated/erlotinib/lapatinib). The confidence interval was estimated by repeating the fitting 100-times. Constants describing conformational equilibria (*K*_1_, *K*_2_) express concentration ratios according to equations (4) and (5), therefore they do not have a unit. The unit of equilibrium constants describing dimerizations (*K*_4_, *K*_7_) is number of receptors/cell.*

### Predictions of the Model for Different Molecular Species

The fitted model parameters not only describe the amount of cell-bound EGF, but they also predict the dependence of different receptor species on ligand concentration and receptor expression level. Calculation of the concentration of liganded receptor species revealed that a substantial fraction of EGF binds to monomeric EGFR with inactive KD, while monomers with active KDs do not contribute to ligand binding (Supplementary Figure 6). The fraction of EGF bound to monomer receptors decreases with increased EGFR expression, a feature, which could account for increased apparent positive cooperativity at high receptor expression levels. Dimers with both symmetric and asymmetric kinase dimers participate in ligand binding with the relative contribution of the latter increasing as a function of EGFR expression. Due to favoring the active conformation of the KD, erlotinib eliminates the binding of EGF to dimers with symmetric kinase dimers almost completely and significantly decreases the fraction of EGF binding to monomeric receptors. As a result, EGF binds almost exclusively to dimers harboring asymmetric kinase dimers in the presence of erlotinib. The model predicts that monomeric receptors with inactive KDs become the dominant EGF binding species in the presence of lapatinib at all receptor expression levels tested. In accordance with its ability to stabilize the inactive conformation of the kinase, lapatinib significantly decreases the percentage of liganded receptor dimers with active KDs.

Apparent cooperativity of ligand binding, captured by the Hill coefficient, is not only determined by the affinity of receptor dimers for the first and second EGF, but also by linkage. Linkage was determined by considering the equivalence of two pathways leading to the formation of doubly liganded receptor dimers: (i) dimerization of liganded receptor monomers, and (ii) dimerization of unliganded receptor monomers followed by successive binding of two EGFs (Figure 2). The dissociation constant for dimerization of a ligand-bound EGFR with inactive KD was found to be $K_{4}\,\,K_{5}\,\,K_{6}\,/\,K_{3}^{2}=9.3\cdot 10^{5}$, which implies negative linkage and decreased homodimerization tendency compared to unliganded monomers with inactive KDs. In contrast, the dissociation constant for homodimerization of liganded receptor monomers with active KD, described by the term $K_{7}\,\,K_{8}\,\,K_{9}\,/\,K_{3}^{2}=84$, is significantly smaller than the same constant for unliganded monomers with active KD implying positive linkage. Due to the very strong dimerization tendency of unliganded and liganded EGFR with active KDs, EGF-bound receptor monomers with active KDs are predicted not to exist since they immediately dimerize upon their formation.

According to the model calculations only four molecular species bind EGF significantly under any of the experimental conditions: monomeric EGFR with inactive KD, singly liganded receptor dimer with asymmetric KD dimer and both kinds of doubly liganded receptor dimers (with symmetric and asymmetric KD dimers). The singly liganded dimer with asymmetric KD dimer exhibits the highest, subnanomolar affinity for EGF, while binding to the other three molecular species is saturated above 10 nM EGF (Supplementary Figure 7). Although there are six different, EGF-binding receptor species (two kinds of monomers with active and inactive KDs, singly and doubly liganded dimers of both kinds), only four of them bind EGF to a significant extent. The EGF-binding affinities of these binding sites is characterized by the dissociation constants in Table 2 (*K*_3_, *K*_5_, *K*_6_ *K*_8_, *K*_9_). It is worth pointing out that the ECD is assumed to adopt an extended conformation in all of these EGF-binding receptor species in the model. Therefore, subtle alterations in the conformations and in the stability of the conformations may account for the different ligand-binding affinities. Along this line, the extended ECD of a receptor monomer exhibits nanomolar EGF affinity (Table 2), but this conformation of the ECD in a dimer with asymmetric KD dimers is characterized by subnanomolar EGF affinity, most likely because the dimeric structure stabilizes the extended conformation. The issue of different cooperativities of the two different receptor dimers with asymmetric and symmetric KD dimers will be further considered in the Discussion. Two of the six EGF binding receptor species do not reach significant concentration for the following reason: (i) the liganded receptor monomer with active KD immediately dimerizes; (ii) the singly liganded receptor dimer with symmetric KD dimer immediately binds the second ligand due to the strong positive cooperativity.

Besides confirming the previous conclusions, calculation of the amount of all kinds of receptor species for all experimental conditions also predicts that a substantial fraction of ligand-independent preformed dimers exist (Supplementary Figure 8). These constitutive dimers harbor kinase domains in a symmetric configuration. A peculiar prediction of these calculations is the lack of dependence of the total amount of receptor dimers on EGF concentration in the control and lapatinib-treated samples. In contrast, EGF induces a slight increase in the fraction of receptor dimers in erlotinib-treated cells.

### EGF-Induced Changes in EGFR Clustering Revealed by Homo-FRET Experiments Are in Agreement With the Model

Although the lack of EGF-dependent recruitment of EGFRs to dimers may sound unexpected, one must bear in mind that the experiments were performed at 4°C, below the phase transition temperature of the plasma membrane, to prevent internalization. In order to confirm the predictions of the model, homo-FRET experiments were performed. Since the donor and the acceptor are spectroscopically identical in homo-FRET, energy migrates in a cluster of such fluorophores (Lidke et al., 2003). Therefore, homo-FRET has already been used extensively for characterizing homoclustering of receptors (Lidke et al., 2003; Yeow and Clayton, 2007; Szabó et al., 2008; Hofman et al., 2010). The extent of energy migration is inversely related to fluorescence anisotropy, the only read-out parameter influenced by homo-FRET. Since anisotropy is not only influenced by homo-FRET, the dependence of anisotropy on the density of fluorophores was utilized to determine the cluster size (the number of fluorophores in a cluster) and the fraction of monomeric, unclustered fluorophores according to a method developed previously (Szabó et al., 2008). Different fluorophore densities were generated by gradual photobleaching of EGFR-GFP.

**FIGURE 4:**
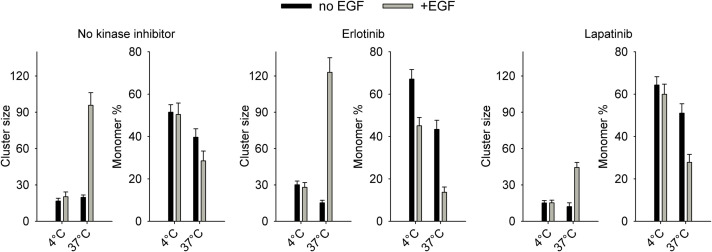
Homoclustering of EGFR in quiescent and EGF-stimulated cells determined by microscopic homo-FRET experiments. Cells were serum-starved overnight followed by a 1-h pretreatment with kinase inhibitors (5 μM, 1 h, 37°C) if indicated. Cells were incubated with 130 nM EGF at 4°C or 37°C for 15 min followed by confocal microscopic determination of the fluorescence anisotropy of EGFR-GFP at different levels of GFP intensity achieved by repeated photobleaching. Fitting of an equation describing the dependence of anisotropy on cluster properties revealed the number of proteins in a homocluster (cluster size) and the fraction of monomeric receptors. The error bars indicate the standard error of the mean determined from four image series.

In control cells without pretreatment with kinase inhibitors, EGF did not induce any change in EGFR homoclustering at 4°C (Figure 4, Supplementary Figure 9). We have shown that 15-min and 60-min incubations were equally ineffective in bringing about changes in EGFR clustering at 4°C (Supplementary Figure 10). These results are in accordance with previous reports revealing that tyrosine phosphorylation of EGFR takes place and reaches saturation in ∼5 min at 4°C, while effects requiring significant lateral diffusion (e.g., internalization) are blocked below the phase transition temperature of the membrane (Campos-Gonzalez and Glenney, 1991; Moehren et al., 2002). Therefore, all homo-FRET experiments were carried out with cells stimulated with EGF for 15 min. As opposed to no effect at 4°C, EGF induced a substantial increase in the cluster size and in the fraction of clustered receptors at 37°C (Figure 4, Supplementary Figure 9). The confidence intervals of the estimations are shown in Supplementary Figure 11, and representative anisotropy images are displayed in Supplementary Figure 12. Erlotinib-pretreated cells responded to EGF with a slight increase in the fraction of clustered receptors at 4°C corroborating this prediction of the model as well. The EGF-induced increase in EGFR clustering at 37°C was augmented by erlotinib pretreatment in agreement with previous results (Coban et al., 2015). While lapatinib did not alter the lack of EGF-induced clustering at 4°C, it slightly decreased the EGF-elicited clustering of EGFR as evidenced by the smaller cluster size.

While the model describing EGF binding only differentiates between monomers and dimers, homo-FRET detects larger clusters as well (Szabó et al., 2008). Therefore, the fraction of dimeric EGFRs and the fraction of clustered receptors in the homo-FRET experiments are not directly comparable. However, changes in dimerization and large-scale clustering are correlated. EGF-induced changes in large-scale clustering only took place at 4°C in our experiments, similar to the requirement for EGF stimulation to be carried out at room temperature or at 37°C so that the growth factor induces dimerization (Gadella and Jovin, 1995). The remarkable correspondence between the homo-FRET experiments and the EGF-dependent changes in EGFR dimerization, shown in Supplementary Figure 8, lends significant support to the model describing ligand binding.

### Experimental Conditions Eliminating Positive Apparent Cooperativity of EGF Binding

**FIGURE 5:**
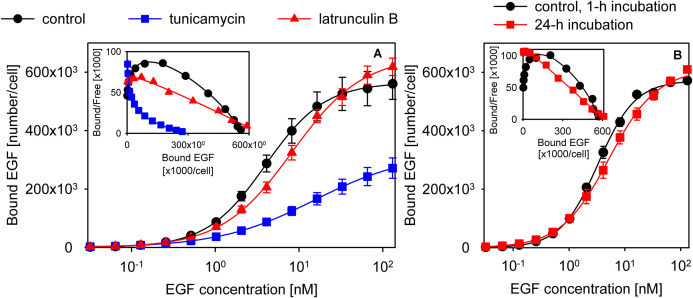
Experimental conditions disturbing positive cooperative binding of EGF. **(A)** The effect of tunicamycin and latrunculin B treatment on apparent cooperativity of EGF binding. Control samples or cells pretreated with tunicamycin or latrunculin B were incubated with a concentration series of EGF to measure the equilibrium binding of the growth factor. The measured data points along with their standard error of the mean and fits according to the Hill equation are shown in the figure. **(B)** Cells were incubated with a concentration series of EGF for 1 h or 24 h, and the equilibrium binding of the growth factor was measured by flow cytometry. The inserts display the Scatchard diagrams.

Since cytoskeletal anchoring has repeatedly been observed to affect ligand binding and activation of EGFR (Wiegant et al., 1986; Lidke et al., 2005; Low-Nam et al., 2011), we tested whether disassembling actin fibers exerts any effect on EGF binding. Latrunculin B treatment disrupted actin fibers and led to a decreased affinity and apparent cooperativity of EGF binding (control cells: *K*_*d*_ = 4 nM (3.9–4.1), *n* = 1.24 (1.21–1.26); latrunculin B-treated cells: *K*_*d*_ = 8.2 nM (7.9–8.5), *n* = 1.03 (1–1.05); the 95% confidence interval is displayed in the parentheses; Figure 5A, Supplementary Figure 13). We also tested if induction of nucleation of actin polymerization by jasplakinolide affects EGF binding. The affinity and apparent cooperativity of EGF binding were not altered by the treatment (Supplementary Figure 14), most likely as a result of jasplakinolide exerting minimal effects on the overall organization of actin filaments and on the subcortical actin meshwork (Supplementary Figure 13). Glycosylation of the ECD has been shown to alter the structure of EGFR significantly (Kaszuba et al., 2015). Tunicamycin treatment successfully deglycosylated EGFR, as evidenced by the decreased molecular weight of the protein (Supplementary Figure 15), and led to an even more pronounced reduction in EGF binding affinity and apparent cooperativity than disrupting actin fibers (*K*_*d*_ = 15 nM (14.6–15.4), *n* = 0.77 (0.75–0.78); Figure 5A). Although the model presented in Figure 2 was fitted to the EGF binding data of tunicamycin- and latrunculin B-treated cells, the results of the fitting were unreproducible and unreliable. We attribute the failure to two circumstances: (i) Since both conditions compromised cell viability, we could not combine these treatments with kinase inhibitors leading to a low number of data points to be fitted. (ii) The low affinity of EGF binding, especially in the tunicamycin-treated cells, resulted in the lack of saturation. Although fitting the Hill equation to the data points does not reveal the molecular background of the observed changes, the results still show that both glycosylation and an intact cytoskeleton are required for positive cooperative EGF binding and for maintaining the affinity of the binding site.

In all of the binding assays reported in the manuscript cells were incubated with EGF on ice for 1 h. The low temperature incubation “freezes” the membrane inhibiting internalization, which would completely invalidate the assumption of equilibrium. However, the low temperature also slows down the rate with which equilibrium binding at the plasma membrane is reached prompting some authors to increase the incubation time to 12–24 h (Macdonald and Pike, 2008; Macdonald-Obermann and Pike, 2018). In order to analyze the effect of a long incubation time EGF binding data obtained with a 1-h and a 24-h incubation with the growth factor were fitted by the Hill equation. The comparison revealed that the long incubation time substantially decreased the apparent cooperativity of EGF binding (1-h incubation: *K*_*d*_ = 3.4 nM (3.3–3.6); *n* = 1.26 (1.23–1.29); 24-h incubation: *K*_*d*_ = 5.2 nM (5–5.5); *n* = 1.02 (0.99–1.05); Figure 5B). Next, we recorded microscopic images of cells incubated with EGF for 1 h or 24 h, and compared the relative amount of fluorescence of TAMRA-EGF from within the intracellular space. Although both incubations were performed on ice, the 24-h incubation led to a significant increase in the intracellular concentration of EGF (Supplementary Figure 16). We concluded that long incubation times allow EGF to be internalized even at low temperatures questioning the assumption that equilibrium binding of EGF to membrane receptors is measured under such experimental conditions.

Since measuring the cell-bound fraction of EGF before reaching equilibrium can increase the apparent cooperativity of ligand binding (see Supplementary Information for a detailed derivation), it was essential to show that the 1-h incubation time used in the experiments is sufficient for EGF binding to reach equilibrium. Time-correlated analysis of flow cytometric data enabled us to conclude that EGF binding is saturated in less than 1 h (Supplementary Figure 17).

## Discussion

In this manuscript we developed and tested a structure-based model for the ligand binding and dimerization of EGFR, whose defining feature is the different dimerization and ligand binding propensity of receptors harboring a kinase in its active or inactive conformation. In order to keep the number of model parameters as low as possible, several kinds of molecular species and processes were neglected, e.g., conformations of the ECD other than the extended and the closed ones, dimerization of monomers with an ECD conformation other than the extended structure, dimers formed only by interactions between the kinase domains, conformational states of the juxta- and transmembrane domains, interactions of the juxtamembrane domain with the membrane and local heterogeneity of receptor concentrations. Oligomerization is not explicitly involved in the model either, although interpretation of the different ligand binding affinities of EGFR dimers harboring active and inactive KDs involves higher-order receptor complexes, a feature of the model to be explained later. Therefore, although the absolute values of the fitted parameters may be inaccurate, the general tendencies and properties have reliably been identified, a conclusion supported by the fact that our experimental data were successfully fitted and that the predictions of the model are in accordance with most published data. Analysis of the fitted model allowed us to reach the following major conclusions: (i) dimers harboring asymmetric and symmetric KD dimers exhibit negative and positive cooperative ligand binding, respectively; (ii) the homodimerization tendency of erlotinib-stabilized active KDs is higher than that of lapatinib-bound, inactive KDs; (iii) the dimerization tendency of liganded EGFR with active KD is stronger than that of unliganded monomers (positive linkage), while the opposite tendency applies to the inactive KD; (iv) both receptor monomers and dimers contribute to EGF binding with the importance of dimers increasing at high receptor expression levels; (v) A significant amount of preformed, ligand-independent dimers harboring inactive, symmetric KD dimers are present, and the fraction of receptor dimers does not change significantly upon ligand binding. While this latter conclusion may seem to be at odds with common sense, the gel-like state of the plasma membrane at the temperature of the experiments (4°C) most likely prevents diffusion-driven alterations of the monomer-dimer equilibrium in the plasma membrane. This prediction is also in accordance with our homo-FRET experiments and with previous results showing that EGF bound to cells on ice only induces receptor dimerization upon elevation of the temperature to 20°C or 37°C (Gadella and Jovin, 1995). The quantitative model proposed by Macdonald and Pike, neglecting structural transitions of the ECD and different conformations of the KD, predicts that EGF binding leads to a decreased fraction of receptor dimers (see recalculation of the model in the Supplementary Material). This feature is the consequence of the lower dimerization propensity of liganded monomers compared to their unliganded counterparts. We believe that predictions of our model are in better agreement with experimental findings than those of the previous model. The fact that our homo-FRET experimental results are in agreement also with the prediction of increased dimerization of EGFRs, whose KD is locked in the active conformation, confirms the major properties of the proposed model. This erlotinib-induced increase in EGFR dimerization has also been reported by Coban et al. (2015).

According to our model, dimerization of EGFR monomers with active and inactive KDs leads to the formation of dimers harboring asymmetric and symmetric KD dimers, respectively. While the formation of asymmetric KD dimers from the active conformation of the kinase is in accordance with the proposed resemblance of EGFR activation to cyclin-induced activation of cyclin-dependent kinases (Zhang et al., 2006; Kovacs et al., 2015), the structural identity of the other KD dimer is dubious. The assumption of two different KD dimers was required for fitting the measurement data successfully. The hypothesis that the KD dimer other than the asymmetric dimer is identical to symmetric KD dimers is somewhat arbitrary. While symmetric KD dimers have been suggested to contain the kinase in the active conformation (Stamos et al., 2002; Landau et al., 2004), our observation that this dimeric species increased in abundance in the presence of lapatinib (Supplementary Figure 8) argues that they contain the kinase in its inactive conformation. While assignment of a specific structure to this KD dimer is arguable based on our experiments, the fact that a KD dimer different from the asymmetric dimer must exist and that this KD dimer is coupled to an ECD whose ligand binding properties are markedly different from the negatively cooperative, high affinity binding site seems certain. We also tested a model in which formation of this dimeric species containing a symmetric kinase dimer began from the “CI” monomer (containing inhibited kinase and closed ECD), but fitting of this model to the experimental data was not successful (data not shown).

Other aspects of the predictions of the proposed model are also in agreement with previous literature data. The fact that a significant fraction of EGF binds to monomeric receptor species may be the consequence of hindered long-range diffusion in the gel-like membrane, an inherent consequence of the experimental condition, but such a phenomenon has repeatedly been reported previously (Teramura et al., 2006; Szabó et al., 2008; Liu et al., 2012; Vámosi et al., 2019). Coupling of a liganded ECD dimer to both active and inactive KD dimers, a key feature in the proposed model, has been experimentally observed in electron microscopy (Mi et al., 2011). Substantial controversy exists in the literature regarding constitutive, ligand-independent EGFR dimers. While several pieces of evidence point at their existence (Clayton et al., 2007; Szabó et al., 2008; Zanetti-Domingues et al., 2018), their transient nature and dependence on receptor expression levels has also been emphasized (Nagy et al., 2010; Low-Nam et al., 2011). Our model calculations show that such constitutive dimers harbor receptors with symmetric, inactive kinase dimers. This conclusion is in agreement with a recent study showing that mutations introduced into the active, asymmetric kinase dimer interface do not significantly affect the stability of ligand-independent, preformed dimers (Byrne et al., 2020). Due to the very low affinity of these dimeric species for binding of the first ligand, they hardly bind EGF at low ligand concentrations, i.e., the fraction of these preformed dimers is constant in this concentration range of EGF. When they do bind EGF beginning from the 1–10 nM range, they do so with positive cooperativity, a prediction, which is in accordance with previous single-molecule experiments (Teramura et al., 2006). There is a perplexing contradiction regarding the cooperativity of ligand binding in the EGFR system. While negative cooperativity has become widely accepted (Macdonald and Pike, 2008; Alvarado et al., 2010; Martin-Fernandez, 2012), positive cooperativity has also been repeatedly observed (Sherrill and Kyte, 1996; Lemmon et al., 1997; Teramura et al., 2006; Chung et al., 2010; Macdonald-Obermann and Pike, 2018). Our model predicts that the type of apparent cooperativity of EGF binding depends on receptor expression levels, and it is attributable to the different ligand binding properties of receptor dimers with the two different kinase dimers. Our observation and prediction that positive cooperativity increases as a function of receptor expression levels have already been reported (Lemmon et al., 1997). The fact that cooperativity of EGF binding depends on the relative abundance of receptors with active and inactive KDs and on receptor expression levels may account for the inconsistency in the literature in this regard.

Among dimers those harboring an inactive, symmetric kinase dimer are the dominant species in the absence or at low concentrations of EGF, while dimers with active, asymmetric kinase dimers are preferred at high ligand concentrations (Supplementary Figure 8). This tendency seems to prevent unintended kinase activation in the absence of stimulation, and to favor initiation of signaling at sufficiently high EGF concentrations. Although the dimerization tendency of unliganded monomers with an inactive KD is weaker than that of unliganded monomers with an active KD, dimers with an inactive KD in the absence or at low concentrations of EGF are favored for the following reasons: (i) the equilibrium between the inactive and active KDs is shifted toward the inactive conformation (*K*_1_ in Table 2); (ii) the binding affinity of dimers with inactive, symmetric kinase dimers for the first ligand is very weak. In contrast, dimers with active, asymmetric KD dimers are favored at high ligand concentrations, which is brought about by the positive linkage between ligand binding and dimer assembly if the KD is in the active conformation and by the inherently stronger dimerization tendency of monomers harboring an active KD.

We identified two conditions, depolymerization of actin filaments and inhibition of glycosylation, which reduced receptor affinity and abolished positive apparent cooperativity of EGF binding. Since the quality of these data did not allow for model fitting, only speculations can be put forward regarding the explanation. Both treatments may inhibit positive apparent cooperativity by reducing the local receptor concentration. Actin depolymerization may achieve this effect by abolishing confinement (Low-Nam et al., 2011), while deglycosylation may reduce the affinity of EGFR to putative raft-like domains or glycolipids (Furukawa et al., 2012). Since deglycosylation has been shown to alter the conformation of EGFR ECD and its orientation relative to the membrane, its affinity for dimerization and ligand binding is expected to be altered (Kaszuba et al., 2015). Since tunicamycin is a general inhibitor of N-glycosylation, it is also possible that the effect of this treatment is attributable to effects on the glycosylation of other proteins.

While the conformations of the extra- and intracellular domains are unlikely to be coupled in monomeric receptors, they can be indirectly linked to each other in dimers or higher order clusters. A possible explanation for the assignment of different EGF affinities to dimers with symmetric and asymmetric KD dimers invoking higher order clusters is provided below. Preformed, unliganded EGFR dimers harbor inactive, symmetric kinase dimers as revealed by the fitting and also supported by literature data (Byrne et al., 2020). Such preformed dimers have been found to form chains or polymers of dimers (Zanetti-Domingues et al., 2018). It is reasonable to assume, as suggested previously, that access of EGF to the ligand binding site of such receptors is blocked explaining their very low affinity for binding the first ligand. Ligand binding must remove these preformed dimers from these receptor polymers since their orientation would not allow cross-phosphorylation to happen. Once they are removed from the receptor polymers to form dimers (not forming larger clusters) (Needham et al., 2016), binding of the second ligand takes place much more easily leading to positive cooperativity. Dimers harboring active kinase domains (asymmetric KD dimers) are not incorporated to dimer chains, therefore the inherent negative cooperativity of the ECD is manifested in their case. Successful fitting of the experimental data using these assumptions implies that the influence of higher-order clusters on the affinity of EGF binding to receptors should not be overlooked.

The proposed model has important implications for interpreting the action of tyrosine kinase inhibitors. These inhibitors not only block the enzymatic activity of the KD, but they also alter the abundance of different molecular species. In particular, EGFR with an active kinase domain has a stronger dimerization tendency than receptors with inactive kinase domains (Table 2). In addition, they also differ in terms of their propensity to form receptor oligomers as explained in the previous paragraph. Therefore, the effect of kinase inhibitors on EGF binding is determined by how they shift the concentration of different receptor states. Inhibitors stabilizing the active conformation of the kinase (e.g., erlotinib) enhance dimerization, a proposition supported by our homo-FRET experiments and previous data (Lichtner et al., 2001; Anido et al., 2003; Coban et al., 2015). In contrast, lapatinib, stabilizing the inactive conformation of the kinase, does not bring about such an increase in receptor dimerization in agreement with previous data (Bublil et al., 2010). Since the kinase in the majority of receptors is already in the inactive conformation in the absence of inhibitors (Table 2), the effect of lapatinib, stabilizing the inactive conformation of the KD, on the monomer/dimer equilibrium is much less pronounced than that of erlotinib. In light of the effect of kinase inhibitors on the monomer/dimer equilibrium, inhibitors stabilizing the inactive conformation of the kinase seem to be more potent and safer from a theoretical point of view.

In conclusion, the model developed in the current manuscript provides a comprehensive view on the molecular transitions taking place upon EGF binding to its receptor. Although different experimental approaches can capture distinct steps of the ligand-induced alterations in the conformation and assembly of receptor dimers, practically none of them can decipher all of them in a quantitative manner. Global analysis of EGF binding equilibria allowed us to generate a model providing insight into most steps of the activation pathway at a pseudo-molecular level.

## Data Availability Statement

All datasets presented in this study are included in the article/Supplementary Material.

## Author Contributions

TH carried out and analyzed most of the experiments and wrote the initial version of the manuscript. TV and IR contributed to the flow cytometric and confocal microscopic experiments, respectively. TK performed part of the experiments with compounds modifying actin polymerization and protein glycosylation. JS advised about the flow cytometric experiments and revised the manuscript. PN conceived, supervised and funded the project, developed the mathematical model and revised the manuscript. All authors contributed to the article and approved the submitted version.

## Conflict of Interest

The authors declare that the research was conducted in the absence of any commercial or financial relationships that could be construed as a potential conflict of interest.
