# Supplementary material for: Comprehensive Model for Epidermal Growth Factor Receptor Ligand Binding Involving Conformational States of the Extracellular and the Kinase Domains
Source: Front Cell Dev Biol. 2020 Aug 11;8:776. doi: 10.3389/fcell.2020.00776 (PMC7431817; doi:10.3389/fcell.2020.00776)
Supplement: Supplementary file 1 [file Data_Sheet_1.PDF]

```
function [boundSignal,allSpecies,whichRoot]=boundEGF(varargin)
% input arguments:
% 3 inputs: ks, rtot, egfcc
% 12 inputs: k1, k2, ..., k10, rtot, egfcc
% k1 - equilibrium between inactive and active kinase
% k2 - equilibrium between closed and extended ECD
% k3 - ligand binding by an extended monomer
% k4 - dimerization of EI
% k5 - first ligand binding of DES
% k6 - second ligand binding of DES
% k7 - dimerization of EA
% k8 - first ligand binding of DEA
% k9 - second ligand binding of DEA
%
% output arguemnts: boundSignal, allSpecies (ci, ca, ei, ea, eil, eal, des,
desl, des2l, dea, deal, dea2l}, whichRoot
%
% 1 ci - closed ECD, inhibited KD
% 2 ca - closed, active KD
% 3 ei - extended, inhibited KD
% 4 ea - extended, active KD
% 5 eil - extended, inhibited KD + EGF
% 6 eal - extended, active KD + EGF
% 7 des - dimer of extended, inhibited (symmetric) KD
% 8 desl - dimer of extended, inhibited (symmetric) KD + EGF
% 9 des2l - dimer of extended, inhibited (symmetric) KD + 2 EGF
% 10 dea - dimer of extended, active (asymmetric) KD
% 11 deal - dimer of extended, active (asymmetric) KD + EGF
% 12 dea2l - dimer of extended, active (asymmetric) KD + 2 EGF
if nargin==11
    k1=varargin{1};
    k2=varargin{2};
    k3=varargin{3};
    k4=varargin{4};
    k5=varargin{5};
    k6=varargin{6};
    k7=varargin{7};
    k8=varargin{8};
    k9=varargin{9};
    rtot=varargin{10};
    egf=varargin{11};
elseif nargin==3
    k1=varargin{1}(1);
    k2=varargin{1}(2);
    k3=varargin{1}(3);
    k4=varargin{1}(4);
    k5=varargin{1}(5);
    k6=varargin{1}(6);
    k7=varargin{1}(7);
    k8=varargin{1}(8);
    k9=varargin{1}(9);
    rtot=varargin{2};
    egf=varargin{3};
else
    error('Number of arguments must be 3 or 11.');
end
% first series of roots
```

```
egfbound1=(1/8).*egf.*k1.^(-1).*(egf.^2.*k3.*k4.*k5.*k6+k3.*(egf.*k4.*k5.*
...
  k6+k4.*k5.*k6.*k8+k1.^2.*(egf.^2+(egf+k5).*k6).*k7.*k8).*k9).^(-2) ...
  .*(8.*egf.^3.*k1.*k3.^2.*(k4.*k5.*k6+k1.^2.*k7.*k8.*k9).^2.*rtot+ ...
  egf.^2.*k1.*k6.*k9.*((-1).*(1+k1).^2.*k4.*k5.*k6.*k7.*k8.*(k4.*k5+ ...
  k1.^2.*k7.*k8).*k9+2.*(1+k1).^2.*(1+k2).*k3.*k4.*k5.*k7.*k8.*(k4.* ...
  k5.*k6+k1.^2.*k7.*k8.*k9)+12.*k3.^2.*(k4.*k5+k1.^2.*k7.*k8).*(k4.* ...
  k5.*k6+k1.^2.*k7.*k8.*k9).*rtot)+k6.*k9.*(2.*k1.^4.*(1+k2).*k3.* ...
  k4.*(k3+k2.*k3+(-2).*k5).*k5.*k6.*k7.^2.*k8.^2.*k9+k1.^5.*k3.*k5.* ...
  k6.*k7.^2.*k8.^2.*k9.*((1+k2).*k4.*(k3+k2.*k3+(-2).*k5)+4.*k3.* ...
  rtot)+k4.*k5.*(k3+k2.*k3+(-2).*k8).*(k1.^2.*k4.*k5.*k6.*k7.*k8.* ...
  k9.*((1+k1).^2.*(egf+k3+k2.*k3).^2.*k4.*k5.*k6.*k7.*k8.*k9+8.* ...
  k3.^2.*(egf.^2.*k4.*k5.*k6+(egf.*k4.*k5.*k6+k4.*k5.*k6.*k8+k1.^2.* ...
  (egf.^2+(egf+k5).*k6).*k7.*k8).*k9).*rtot)).^(1/2)+k1.^2.*k7.*k8.* ...
  (2.*(1+k2).^2.*k3.^2.*k4.^2.*k5.^2.*k6.*k9+(-2).*k5.*(k1.^2.*k4.* ...
  k5.*k6.*k7.*k8.*k9.*((1+k1).^2.*(egf+k3+k2.*k3).^2.*k4.*k5.*k6.* ...
  k7.*k8.*k9+8.*k3.^2.*(egf.^2.*k4.*k5.*k6+(egf.*k4.*k5.*k6+k4.*k5.* ...
  k6.*k8+k1.^2.*(egf.^2+(egf+k5).*k6).*k7.*k8).*k9).*rtot)).^(1/2)+( ...
  1+k2).*k3.*((-4).*k4.^2.*k5.^2.*k6.*k8.*k9+(k1.^2.*k4.*k5.*k6.* ...
  k7.*k8.*k9.*((1+k1).^2.*(egf+k3+k2.*k3).^2.*k4.*k5.*k6.*k7.*k8.* ...
  k9+8.*k3.^2.*(egf.^2.*k4.*k5.*k6+(egf.*k4.*k5.*k6+k4.*k5.*k6.*k8+ ...
  k1.^2.*(egf.^2+(egf+k5).*k6).*k7.*k8).*k9).*rtot)).^(1/2)))+ ...
  k1.^3.*k7.*k8.*(k3.^2.*k4.*k5.*k6.*k9.*((1+k2).^2.*(k4.*k5+k7.*k8) ...
  +4.*(k5+k8).*rtot)+(-2).*k5.*(k1.^2.*k4.*k5.*k6.*k7.*k8.*k9.*((1+ ...
  k1).^2.*(egf+k3+k2.*k3).^2.*k4.*k5.*k6.*k7.*k8.*k9+8.*k3.^2.*( ...
  egf.^2.*k4.*k5.*k6+(egf.*k4.*k5.*k6+k4.*k5.*k6.*k8+k1.^2.*(egf.^2+ ...
  (egf+k5).*k6).*k7.*k8).*k9).*rtot)).^(1/2)+(1+k2).*k3.*((-2).*k4.* ...
  k5.^2.*k6.*(k4+k7).*k8.*k9+(k1.^2.*k4.*k5.*k6.*k7.*k8.*k9.*((1+k1) ...
  .^2.*(egf+k3+k2.*k3).^2.*k4.*k5.*k6.*k7.*k8.*k9+8.*k3.^2.*( ...
  egf.^2.*k4.*k5.*k6+(egf.*k4.*k5.*k6+k4.*k5.*k6.*k8+k1.^2.*(egf.^2+ ...
  (egf+k5).*k6).*k7.*k8).*k9).*rtot)).^(1/2)))+k1.*k4.*k5.*(k3.^2.* ...
  k4.*k5.*k6.*k8.*k9.*((1+k2).^2.*k7+4.*rtot)+(-2).*k8.*(k1.^2.*k4.* ...
  k5.*k6.*k7.*k8.*k9.*((1+k1).^2.*(egf+k3+k2.*k3).^2.*k4.*k5.*k6.* ...
  k7.*k8.*k9+8.*k3.^2.*(egf.^2.*k4.*k5.*k6+(egf.*k4.*k5.*k6+k4.*k5.* ...
  k6.*k8+k1.^2.*(egf.^2+(egf+k5).*k6).*k7.*k8).*k9).*rtot)).^(1/2)+( ...
  1+k2).*k3.*((-2).*k4.*k5.*k6.*k7.*k8.^2.*k9+(k1.^2.*k4.*k5.*k6.* ...
  k7.*k8.*k9.*((1+k1).^2.*(egf+k3+k2.*k3).^2.*k4.*k5.*k6.*k7.*k8.* ...
  k9+8.*k3.^2.*(egf.^2.*k4.*k5.*k6+(egf.*k4.*k5.*k6+k4.*k5.*k6.*k8+ ...
  k1.^2.*(egf.^2+(egf+k5).*k6).*k7.*k8).*k9).*rtot)).^(1/2))))+egf.* ...
  (4.*k1.^4.*k4.*k5.*k6.*((1+k2).^2.*k3.^2+(-1).*k5.*k6).*k7.^2.* ...
  k8.^2.*k9.^2+k4.*k5.*k6.*(2.*(1+k2).*k3+(-1).*k9).*(k1.^2.*k4.* ...
  k5.*k6.*k7.*k8.*k9.*((1+k1).^2.*(egf+k3+k2.*k3).^2.*k4.*k5.*k6.* ...
  k7.*k8.*k9+8.*k3.^2.*(egf.^2.*k4.*k5.*k6+(egf.*k4.*k5.*k6+k4.*k5.* ...
  k6.*k8+k1.^2.*(egf.^2+(egf+k5).*k6).*k7.*k8).*k9).*rtot)).^(1/2)+ ...
  2.*k1.^5.*k6.*k7.^2.*k8.^2.*k9.^2.*((-1).*k4.*k5.^2.*k6+k3.^2.*(( ...
  1+k2).^2.*k4.*k5+2.*(2.*k5+k6).*rtot))+k1.^3.*k7.*k8.*k9.*(2.* ...
  k3.^2.*k4.*k5.*k6.*((1+k2).^2.*(k4.*k5.*k6+k7.*k8.*k9)+4.*(k5.*k6+ ...
  (k6+k8).*k9).*rtot)+2.*(1+k2).*k3.*(k1.^2.*k4.*k5.*k6.*k7.*k8.* ...
  k9.*((1+k1).^2.*(egf+k3+k2.*k3).^2.*k4.*k5.*k6.*k7.*k8.*k9+8.* ...
  k3.^2.*(egf.^2.*k4.*k5.*k6+(egf.*k4.*k5.*k6+k4.*k5.*k6.*k8+k1.^2.* ...
  (egf.^2+(egf+k5).*k6).*k7.*k8).*k9).*rtot)).^(1/2)+k6.*((-2).*k4.* ...
  k5.^2.*k6.*(k4+k7).*k8.*k9+(-1).*(k1.^2.*k4.*k5.*k6.*k7.*k8.*k9.*( ...
  (1+k1).^2.*(egf+k3+k2.*k3).^2.*k4.*k5.*k6.*k7.*k8.*k9+8.*k3.^2.*( ...
  egf.^2.*k4.*k5.*k6+(egf.*k4.*k5.*k6+k4.*k5.*k6.*k8+k1.^2.*(egf.^2+ ...
  (egf+k5).*k6).*k7.*k8).*k9).*rtot)).^(1/2)))+k1.^2.*k7.*k8.*k9.*( ...
  4.*(1+k2).^2.*k3.^2.*k4.^2.*k5.^2.*k6.^2+2.*(1+k2).*k3.*(k1.^2.* ...
  k4.*k5.*k6.*k7.*k8.*k9.*((1+k1).^2.*(egf+k3+k2.*k3).^2.*k4.*k5.* ...
```

```
  k6.*k7.*k8.*k9+8.*k3.^2.*(egf.^2.*k4.*k5.*k6+(egf.*k4.*k5.*k6+k4.* ...
  k5.*k6.*k8+k1.^2.*(egf.^2+(egf+k5).*k6).*k7.*k8).*k9).*rtot)).^( ...
  1/2)+(-1).*k6.*(4.*k4.^2.*k5.^2.*k6.*k8.*k9+(k1.^2.*k4.*k5.*k6.* ...
  k7.*k8.*k9.*((1+k1).^2.*(egf+k3+k2.*k3).^2.*k4.*k5.*k6.*k7.*k8.* ...
  k9+8.*k3.^2.*(egf.^2.*k4.*k5.*k6+(egf.*k4.*k5.*k6+k4.*k5.*k6.*k8+ ...
  k1.^2.*(egf.^2+(egf+k5).*k6).*k7.*k8).*k9).*rtot)).^(1/2)))+k1.* ...
  k4.*k5.*k6.*(2.*k3.^2.*k4.*k5.*k6.*k9.*((1+k2).^2.*k7.*k8+2.*(2.* ...
  k8+k9).*rtot)+2.*(1+k2).*k3.*(k1.^2.*k4.*k5.*k6.*k7.*k8.*k9.*((1+ ...
  k1).^2.*(egf+k3+k2.*k3).^2.*k4.*k5.*k6.*k7.*k8.*k9+8.*k3.^2.*( ...
  egf.^2.*k4.*k5.*k6+(egf.*k4.*k5.*k6+k4.*k5.*k6.*k8+k1.^2.*(egf.^2+ ...
  (egf+k5).*k6).*k7.*k8).*k9).*rtot)).^(1/2)+(-1).*k9.*(2.*k4.*k5.* ...
  k6.*k7.*k8.^2.*k9+(k1.^2.*k4.*k5.*k6.*k7.*k8.*k9.*((1+k1).^2.*( ...
  egf+k3+k2.*k3).^2.*k4.*k5.*k6.*k7.*k8.*k9+8.*k3.^2.*(egf.^2.*k4.* ...
  k5.*k6+(egf.*k4.*k5.*k6+k4.*k5.*k6.*k8+k1.^2.*(egf.^2+(egf+k5).* ...
  k6).*k7.*k8).*k9).*rtot)).^(1/2)))));


ci1=(1/4).*k2.*k3.^(-1).*(egf.^2.*k4.*k5.*k6+(egf.*k4.*k5.*k6+k4.*k5.* ...
  k6.*k8+k1.^2.*(egf.^2+(egf+k5).*k6).*k7.*k8).*k9).^(-1).*((-1).* ...
  k1.*(1+k1).*(egf+k3+k2.*k3).*k4.*k5.*k6.*k7.*k8.*k9+(-1).*(k1.^2.* ...
  k4.*k5.*k6.*k7.*k8.*k9.*((1+k1).^2.*(egf+k3+k2.*k3).^2.*k4.*k5.* ...
  k6.*k7.*k8.*k9+8.*k3.^2.*(egf.^2.*k4.*k5.*k6+(egf.*k4.*k5.*k6+k4.* ...
  k5.*k6.*k8+k1.^2.*(egf.^2+(egf+k5).*k6).*k7.*k8).*k9).*rtot)).^( ...
  1/2));


ca1=(1/4).*k1.^(-1).*k2.*k3.^(-1).*(egf.^2.*k4.*k5.*k6+(egf.*k4.*k5.* ...
  k6+k4.*k5.*k6.*k8+k1.^2.*(egf.^2+(egf+k5).*k6).*k7.*k8).*k9).^(-1) ...
  .*((-1).*k1.*(1+k1).*(egf+k3+k2.*k3).*k4.*k5.*k6.*k7.*k8.*k9+(-1) ...
  .*(k1.^2.*k4.*k5.*k6.*k7.*k8.*k9.*((1+k1).^2.*(egf+k3+k2.*k3).^2.* ...
  k4.*k5.*k6.*k7.*k8.*k9+8.*k3.^2.*(egf.^2.*k4.*k5.*k6+(egf.*k4.* ...
  k5.*k6+k4.*k5.*k6.*k8+k1.^2.*(egf.^2+(egf+k5).*k6).*k7.*k8).*k9).* ...
  rtot)).^(1/2));


ei1=(-1/4).*k3.^(-1).*(egf.^2.*k4.*k5.*k6+(egf.*k4.*k5.*k6+k4.*k5.* ...
  k6.*k8+k1.^2.*(egf.^2+(egf+k5).*k6).*k7.*k8).*k9).^(-1).*(k1.*(1+ ...
  k1).*(egf+k3+k2.*k3).*k4.*k5.*k6.*k7.*k8.*k9+(k1.^2.*k4.*k5.*k6.* ...
  k7.*k8.*k9.*((1+k1).^2.*(egf+k3+k2.*k3).^2.*k4.*k5.*k6.*k7.*k8.* ...
  k9+8.*k3.^2.*(egf.^2.*k4.*k5.*k6+(egf.*k4.*k5.*k6+k4.*k5.*k6.*k8+ ...
  k1.^2.*(egf.^2+(egf+k5).*k6).*k7.*k8).*k9).*rtot)).^(1/2));


ea1=(-1/4).*k1.^(-1).*k3.^(-1).*(egf.^2.*k4.*k5.*k6+(egf.*k4.*k5.*k6+ ...
  k4.*k5.*k6.*k8+k1.^2.*(egf.^2+(egf+k5).*k6).*k7.*k8).*k9).^(-1).*( ...
  k1.*(1+k1).*(egf+k3+k2.*k3).*k4.*k5.*k6.*k7.*k8.*k9+(k1.^2.*k4.* ...
  k5.*k6.*k7.*k8.*k9.*((1+k1).^2.*(egf+k3+k2.*k3).^2.*k4.*k5.*k6.* ...
  k7.*k8.*k9+8.*k3.^2.*(egf.^2.*k4.*k5.*k6+(egf.*k4.*k5.*k6+k4.*k5.* ...
  k6.*k8+k1.^2.*(egf.^2+(egf+k5).*k6).*k7.*k8).*k9).*rtot)).^(1/2)); ...



eil1=(1/4).*egf.*k3.^(-2).*(egf.^2.*k4.*k5.*k6+(egf.*k4.*k5.*k6+k4.* ...
  k5.*k6.*k8+k1.^2.*(egf.^2+(egf+k5).*k6).*k7.*k8).*k9).^(-1).*((-1) ...
  .*k1.*(1+k1).*(egf+k3+k2.*k3).*k4.*k5.*k6.*k7.*k8.*k9+(-1).*( ...
```

```
  k1.^2.*k4.*k5.*k6.*k7.*k8.*k9.*((1+k1).^2.*(egf+k3+k2.*k3).^2.* ...
  k4.*k5.*k6.*k7.*k8.*k9+8.*k3.^2.*(egf.^2.*k4.*k5.*k6+(egf.*k4.* ...
  k5.*k6+k4.*k5.*k6.*k8+k1.^2.*(egf.^2+(egf+k5).*k6).*k7.*k8).*k9).* ...
  rtot)).^(1/2));


eal1=(1/4).*egf.*k1.^(-1).*k3.^(-2).*(egf.^2.*k4.*k5.*k6+(egf.*k4.*k5.* ...
  k6+k4.*k5.*k6.*k8+k1.^2.*(egf.^2+(egf+k5).*k6).*k7.*k8).*k9).^(-1) ...
  .*((-1).*k1.*(1+k1).*(egf+k3+k2.*k3).*k4.*k5.*k6.*k7.*k8.*k9+(-1) ...
  .*(k1.^2.*k4.*k5.*k6.*k7.*k8.*k9.*((1+k1).^2.*(egf+k3+k2.*k3).^2.* ...
  k4.*k5.*k6.*k7.*k8.*k9+8.*k3.^2.*(egf.^2.*k4.*k5.*k6+(egf.*k4.* ...
  k5.*k6+k4.*k5.*k6.*k8+k1.^2.*(egf.^2+(egf+k5).*k6).*k7.*k8).*k9).* ...
  rtot)).^(1/2));


des1=(1/8).*k1.*k5.*k6.*k7.*k8.*k9.*(egf.^2.*k3.*k4.*k5.*k6+k3.*(egf.* ...
  k4.*k5.*k6+k4.*k5.*k6.*k8+k1.^2.*(egf.^2+(egf+k5).*k6).*k7.*k8).* ...
  k9).^(-2).*(egf.^2.*k1.*((1+k1).^2.*k4.*k5.*k6.*k7.*k8.*k9+4.* ...
  k3.^2.*(k4.*k5.*k6+k1.^2.*k7.*k8.*k9).*rtot)+k3.*(2.*k1.^2.*(1+k2) ...
  .^2.*k3.*k4.*k5.*k6.*k7.*k8.*k9+k1.^3.*k3.*k5.*k6.*k7.*k8.*k9.*(( ...
  1+k2).^2.*k4+4.*rtot)+k1.*k3.*k4.*k5.*k6.*k8.*k9.*((1+k2).^2.*k7+ ...
  4.*rtot)+(1+k2).*(k1.^2.*k4.*k5.*k6.*k7.*k8.*k9.*((1+k1).^2.*(egf+ ...
  k3+k2.*k3).^2.*k4.*k5.*k6.*k7.*k8.*k9+8.*k3.^2.*(egf.^2.*k4.*k5.* ...
  k6+(egf.*k4.*k5.*k6+k4.*k5.*k6.*k8+k1.^2.*(egf.^2+(egf+k5).*k6).* ...
  k7.*k8).*k9).*rtot)).^(1/2)+k1.*(1+k2).*(k1.^2.*k4.*k5.*k6.*k7.* ...
  k8.*k9.*((1+k1).^2.*(egf+k3+k2.*k3).^2.*k4.*k5.*k6.*k7.*k8.*k9+8.* ...
  k3.^2.*(egf.^2.*k4.*k5.*k6+(egf.*k4.*k5.*k6+k4.*k5.*k6.*k8+k1.^2.* ...
  (egf.^2+(egf+k5).*k6).*k7.*k8).*k9).*rtot)).^(1/2))+egf.*((k1.^2.* ...
  k4.*k5.*k6.*k7.*k8.*k9.*((1+k1).^2.*(egf+k3+k2.*k3).^2.*k4.*k5.* ...
  k6.*k7.*k8.*k9+8.*k3.^2.*(egf.^2.*k4.*k5.*k6+(egf.*k4.*k5.*k6+k4.* ...
  k5.*k6.*k8+k1.^2.*(egf.^2+(egf+k5).*k6).*k7.*k8).*k9).*rtot)).^( ...
  1/2)+k1.*(2.*k3.*k6.*k9.*((1+k1).^2.*(1+k2).*k4.*k5.*k7.*k8+2.* ...
  k3.*(k4.*k5+k1.^2.*k7.*k8).*rtot)+(k1.^2.*k4.*k5.*k6.*k7.*k8.*k9.* ...
  ((1+k1).^2.*(egf+k3+k2.*k3).^2.*k4.*k5.*k6.*k7.*k8.*k9+8.*k3.^2.*( ...
  egf.^2.*k4.*k5.*k6+(egf.*k4.*k5.*k6+k4.*k5.*k6.*k8+k1.^2.*(egf.^2+ ...
  (egf+k5).*k6).*k7.*k8).*k9).*rtot)).^(1/2))));


dea1=(1/8).*k1.^(-1).*k4.*k5.*k6.*k8.*k9.*(egf.^2.*k3.*k4.*k5.*k6+k3.*( ...
  egf.*k4.*k5.*k6+k4.*k5.*k6.*k8+k1.^2.*(egf.^2+(egf+k5).*k6).*k7.* ...
  k8).*k9).^(-2).*(egf.^2.*k1.*((1+k1).^2.*k4.*k5.*k6.*k7.*k8.*k9+ ...
  4.*k3.^2.*(k4.*k5.*k6+k1.^2.*k7.*k8.*k9).*rtot)+k3.*(2.*k1.^2.*(1+ ...
  k2).^2.*k3.*k4.*k5.*k6.*k7.*k8.*k9+k1.^3.*k3.*k5.*k6.*k7.*k8.*k9.* ...
  ((1+k2).^2.*k4+4.*rtot)+k1.*k3.*k4.*k5.*k6.*k8.*k9.*((1+k2).^2.* ...
  k7+4.*rtot)+(1+k2).*(k1.^2.*k4.*k5.*k6.*k7.*k8.*k9.*((1+k1).^2.*( ...
  egf+k3+k2.*k3).^2.*k4.*k5.*k6.*k7.*k8.*k9+8.*k3.^2.*(egf.^2.*k4.* ...
  k5.*k6+(egf.*k4.*k5.*k6+k4.*k5.*k6.*k8+k1.^2.*(egf.^2+(egf+k5).* ...
  k6).*k7.*k8).*k9).*rtot)).^(1/2)+k1.*(1+k2).*(k1.^2.*k4.*k5.*k6.* ...
  k7.*k8.*k9.*((1+k1).^2.*(egf+k3+k2.*k3).^2.*k4.*k5.*k6.*k7.*k8.* ...
  k9+8.*k3.^2.*(egf.^2.*k4.*k5.*k6+(egf.*k4.*k5.*k6+k4.*k5.*k6.*k8+ ...
  k1.^2.*(egf.^2+(egf+k5).*k6).*k7.*k8).*k9).*rtot)).^(1/2))+egf.*(( ...
  k1.^2.*k4.*k5.*k6.*k7.*k8.*k9.*((1+k1).^2.*(egf+k3+k2.*k3).^2.* ...
  k4.*k5.*k6.*k7.*k8.*k9+8.*k3.^2.*(egf.^2.*k4.*k5.*k6+(egf.*k4.* ...
  k5.*k6+k4.*k5.*k6.*k8+k1.^2.*(egf.^2+(egf+k5).*k6).*k7.*k8).*k9).* ...
  rtot)).^(1/2)+k1.*(2.*k3.*k6.*k9.*((1+k1).^2.*(1+k2).*k4.*k5.*k7.* ...
  k8+2.*k3.*(k4.*k5+k1.^2.*k7.*k8).*rtot)+(k1.^2.*k4.*k5.*k6.*k7.* ...
```

```
  k8.*k9.*((1+k1).^2.*(egf+k3+k2.*k3).^2.*k4.*k5.*k6.*k7.*k8.*k9+8.* ...
  k3.^2.*(egf.^2.*k4.*k5.*k6+(egf.*k4.*k5.*k6+k4.*k5.*k6.*k8+k1.^2.* ...
  (egf.^2+(egf+k5).*k6).*k7.*k8).*k9).*rtot)).^(1/2))));


desl1=(1/8).*egf.*k1.*k6.*k7.*k8.*k9.*(egf.^2.*k3.*k4.*k5.*k6+k3.*(egf.* ...
  k4.*k5.*k6+k4.*k5.*k6.*k8+k1.^2.*(egf.^2+(egf+k5).*k6).*k7.*k8).* ...
  k9).^(-2).*(egf.^2.*k1.*((1+k1).^2.*k4.*k5.*k6.*k7.*k8.*k9+4.* ...
  k3.^2.*(k4.*k5.*k6+k1.^2.*k7.*k8.*k9).*rtot)+k3.*(2.*k1.^2.*(1+k2) ...
  .^2.*k3.*k4.*k5.*k6.*k7.*k8.*k9+k1.^3.*k3.*k5.*k6.*k7.*k8.*k9.*(( ...
  1+k2).^2.*k4+4.*rtot)+k1.*k3.*k4.*k5.*k6.*k8.*k9.*((1+k2).^2.*k7+ ...
  4.*rtot)+(1+k2).*(k1.^2.*k4.*k5.*k6.*k7.*k8.*k9.*((1+k1).^2.*(egf+ ...
  k3+k2.*k3).^2.*k4.*k5.*k6.*k7.*k8.*k9+8.*k3.^2.*(egf.^2.*k4.*k5.* ...
  k6+(egf.*k4.*k5.*k6+k4.*k5.*k6.*k8+k1.^2.*(egf.^2+(egf+k5).*k6).* ...
  k7.*k8).*k9).*rtot)).^(1/2)+k1.*(1+k2).*(k1.^2.*k4.*k5.*k6.*k7.* ...
  k8.*k9.*((1+k1).^2.*(egf+k3+k2.*k3).^2.*k4.*k5.*k6.*k7.*k8.*k9+8.* ...
  k3.^2.*(egf.^2.*k4.*k5.*k6+(egf.*k4.*k5.*k6+k4.*k5.*k6.*k8+k1.^2.* ...
  (egf.^2+(egf+k5).*k6).*k7.*k8).*k9).*rtot)).^(1/2))+egf.*((k1.^2.* ...
  k4.*k5.*k6.*k7.*k8.*k9.*((1+k1).^2.*(egf+k3+k2.*k3).^2.*k4.*k5.* ...
  k6.*k7.*k8.*k9+8.*k3.^2.*(egf.^2.*k4.*k5.*k6+(egf.*k4.*k5.*k6+k4.* ...
  k5.*k6.*k8+k1.^2.*(egf.^2+(egf+k5).*k6).*k7.*k8).*k9).*rtot)).^( ...
  1/2)+k1.*(2.*k3.*k6.*k9.*((1+k1).^2.*(1+k2).*k4.*k5.*k7.*k8+2.* ...
  k3.*(k4.*k5+k1.^2.*k7.*k8).*rtot)+(k1.^2.*k4.*k5.*k6.*k7.*k8.*k9.* ...
  ((1+k1).^2.*(egf+k3+k2.*k3).^2.*k4.*k5.*k6.*k7.*k8.*k9+8.*k3.^2.*( ...
  egf.^2.*k4.*k5.*k6+(egf.*k4.*k5.*k6+k4.*k5.*k6.*k8+k1.^2.*(egf.^2+ ...
  (egf+k5).*k6).*k7.*k8).*k9).*rtot)).^(1/2))));


des2l1=(1/8).*egf.^2.*k1.*k7.*k8.*k9.*(egf.^2.*k3.*k4.*k5.*k6+k3.*(egf.* ...
  k4.*k5.*k6+k4.*k5.*k6.*k8+k1.^2.*(egf.^2+(egf+k5).*k6).*k7.*k8).* ...
  k9).^(-2).*(egf.^2.*k1.*((1+k1).^2.*k4.*k5.*k6.*k7.*k8.*k9+4.* ...
  k3.^2.*(k4.*k5.*k6+k1.^2.*k7.*k8.*k9).*rtot)+k3.*(2.*k1.^2.*(1+k2) ...
  .^2.*k3.*k4.*k5.*k6.*k7.*k8.*k9+k1.^3.*k3.*k5.*k6.*k7.*k8.*k9.*(( ...
  1+k2).^2.*k4+4.*rtot)+k1.*k3.*k4.*k5.*k6.*k8.*k9.*((1+k2).^2.*k7+ ...
  4.*rtot)+(1+k2).*(k1.^2.*k4.*k5.*k6.*k7.*k8.*k9.*((1+k1).^2.*(egf+ ...
  k3+k2.*k3).^2.*k4.*k5.*k6.*k7.*k8.*k9+8.*k3.^2.*(egf.^2.*k4.*k5.* ...
  k6+(egf.*k4.*k5.*k6+k4.*k5.*k6.*k8+k1.^2.*(egf.^2+(egf+k5).*k6).* ...
  k7.*k8).*k9).*rtot)).^(1/2)+k1.*(1+k2).*(k1.^2.*k4.*k5.*k6.*k7.* ...
  k8.*k9.*((1+k1).^2.*(egf+k3+k2.*k3).^2.*k4.*k5.*k6.*k7.*k8.*k9+8.* ...
  k3.^2.*(egf.^2.*k4.*k5.*k6+(egf.*k4.*k5.*k6+k4.*k5.*k6.*k8+k1.^2.* ...
  (egf.^2+(egf+k5).*k6).*k7.*k8).*k9).*rtot)).^(1/2))+egf.*((k1.^2.* ...
  k4.*k5.*k6.*k7.*k8.*k9.*((1+k1).^2.*(egf+k3+k2.*k3).^2.*k4.*k5.* ...
  k6.*k7.*k8.*k9+8.*k3.^2.*(egf.^2.*k4.*k5.*k6+(egf.*k4.*k5.*k6+k4.* ...
  k5.*k6.*k8+k1.^2.*(egf.^2+(egf+k5).*k6).*k7.*k8).*k9).*rtot)).^( ...
  1/2)+k1.*(2.*k3.*k6.*k9.*((1+k1).^2.*(1+k2).*k4.*k5.*k7.*k8+2.* ...
  k3.*(k4.*k5+k1.^2.*k7.*k8).*rtot)+(k1.^2.*k4.*k5.*k6.*k7.*k8.*k9.* ...
  ((1+k1).^2.*(egf+k3+k2.*k3).^2.*k4.*k5.*k6.*k7.*k8.*k9+8.*k3.^2.*( ...
  egf.^2.*k4.*k5.*k6+(egf.*k4.*k5.*k6+k4.*k5.*k6.*k8+k1.^2.*(egf.^2+ ...
  (egf+k5).*k6).*k7.*k8).*k9).*rtot)).^(1/2))));


deal1=(1/8).*egf.*k1.^(-1).*k4.*k5.*k6.*k9.*(egf.^2.*k3.*k4.*k5.*k6+k3.* ...
  (egf.*k4.*k5.*k6+k4.*k5.*k6.*k8+k1.^2.*(egf.^2+(egf+k5).*k6).*k7.* ...
  k8).*k9).^(-2).*(egf.^2.*k1.*((1+k1).^2.*k4.*k5.*k6.*k7.*k8.*k9+ ...
  4.*k3.^2.*(k4.*k5.*k6+k1.^2.*k7.*k8.*k9).*rtot)+k3.*(2.*k1.^2.*(1+ ...
  k2).^2.*k3.*k4.*k5.*k6.*k7.*k8.*k9+k1.^3.*k3.*k5.*k6.*k7.*k8.*k9.* ...
```

```
  ((1+k2).^2.*k4+4.*rtot)+k1.*k3.*k4.*k5.*k6.*k8.*k9.*((1+k2).^2.* ...
  k7+4.*rtot)+(1+k2).*(k1.^2.*k4.*k5.*k6.*k7.*k8.*k9.*((1+k1).^2.*( ...
  egf+k3+k2.*k3).^2.*k4.*k5.*k6.*k7.*k8.*k9+8.*k3.^2.*(egf.^2.*k4.* ...
  k5.*k6+(egf.*k4.*k5.*k6+k4.*k5.*k6.*k8+k1.^2.*(egf.^2+(egf+k5).* ...
  k6).*k7.*k8).*k9).*rtot)).^(1/2)+k1.*(1+k2).*(k1.^2.*k4.*k5.*k6.* ...
  k7.*k8.*k9.*((1+k1).^2.*(egf+k3+k2.*k3).^2.*k4.*k5.*k6.*k7.*k8.* ...
  k9+8.*k3.^2.*(egf.^2.*k4.*k5.*k6+(egf.*k4.*k5.*k6+k4.*k5.*k6.*k8+ ...
  k1.^2.*(egf.^2+(egf+k5).*k6).*k7.*k8).*k9).*rtot)).^(1/2))+egf.*(( ...
  k1.^2.*k4.*k5.*k6.*k7.*k8.*k9.*((1+k1).^2.*(egf+k3+k2.*k3).^2.* ...
  k4.*k5.*k6.*k7.*k8.*k9+8.*k3.^2.*(egf.^2.*k4.*k5.*k6+(egf.*k4.* ...
  k5.*k6+k4.*k5.*k6.*k8+k1.^2.*(egf.^2+(egf+k5).*k6).*k7.*k8).*k9).* ...
  rtot)).^(1/2)+k1.*(2.*k3.*k6.*k9.*((1+k1).^2.*(1+k2).*k4.*k5.*k7.* ...
  k8+2.*k3.*(k4.*k5+k1.^2.*k7.*k8).*rtot)+(k1.^2.*k4.*k5.*k6.*k7.* ...
  k8.*k9.*((1+k1).^2.*(egf+k3+k2.*k3).^2.*k4.*k5.*k6.*k7.*k8.*k9+8.* ...
  k3.^2.*(egf.^2.*k4.*k5.*k6+(egf.*k4.*k5.*k6+k4.*k5.*k6.*k8+k1.^2.* ...
  (egf.^2+(egf+k5).*k6).*k7.*k8).*k9).*rtot)).^(1/2))));


dea2l1=(1/8).*egf.^2.*k1.^(-1).*k4.*k5.*k6.*(egf.^2.*k3.*k4.*k5.*k6+k3.*( ...
  egf.*k4.*k5.*k6+k4.*k5.*k6.*k8+k1.^2.*(egf.^2+(egf+k5).*k6).*k7.* ...
  k8).*k9).^(-2).*(egf.^2.*k1.*((1+k1).^2.*k4.*k5.*k6.*k7.*k8.*k9+ ...
  4.*k3.^2.*(k4.*k5.*k6+k1.^2.*k7.*k8.*k9).*rtot)+k3.*(2.*k1.^2.*(1+ ...
  k2).^2.*k3.*k4.*k5.*k6.*k7.*k8.*k9+k1.^3.*k3.*k5.*k6.*k7.*k8.*k9.* ...
  ((1+k2).^2.*k4+4.*rtot)+k1.*k3.*k4.*k5.*k6.*k8.*k9.*((1+k2).^2.* ...
  k7+4.*rtot)+(1+k2).*(k1.^2.*k4.*k5.*k6.*k7.*k8.*k9.*((1+k1).^2.*( ...
  egf+k3+k2.*k3).^2.*k4.*k5.*k6.*k7.*k8.*k9+8.*k3.^2.*(egf.^2.*k4.* ...
  k5.*k6+(egf.*k4.*k5.*k6+k4.*k5.*k6.*k8+k1.^2.*(egf.^2+(egf+k5).* ...
  k6).*k7.*k8).*k9).*rtot)).^(1/2)+k1.*(1+k2).*(k1.^2.*k4.*k5.*k6.* ...
  k7.*k8.*k9.*((1+k1).^2.*(egf+k3+k2.*k3).^2.*k4.*k5.*k6.*k7.*k8.* ...
  k9+8.*k3.^2.*(egf.^2.*k4.*k5.*k6+(egf.*k4.*k5.*k6+k4.*k5.*k6.*k8+ ...
  k1.^2.*(egf.^2+(egf+k5).*k6).*k7.*k8).*k9).*rtot)).^(1/2))+egf.*(( ...
  k1.^2.*k4.*k5.*k6.*k7.*k8.*k9.*((1+k1).^2.*(egf+k3+k2.*k3).^2.* ...
  k4.*k5.*k6.*k7.*k8.*k9+8.*k3.^2.*(egf.^2.*k4.*k5.*k6+(egf.*k4.* ...
  k5.*k6+k4.*k5.*k6.*k8+k1.^2.*(egf.^2+(egf+k5).*k6).*k7.*k8).*k9).* ...
  rtot)).^(1/2)+k1.*(2.*k3.*k6.*k9.*((1+k1).^2.*(1+k2).*k4.*k5.*k7.* ...
  k8+2.*k3.*(k4.*k5+k1.^2.*k7.*k8).*rtot)+(k1.^2.*k4.*k5.*k6.*k7.* ...
  k8.*k9.*((1+k1).^2.*(egf+k3+k2.*k3).^2.*k4.*k5.*k6.*k7.*k8.*k9+8.* ...
  k3.^2.*(egf.^2.*k4.*k5.*k6+(egf.*k4.*k5.*k6+k4.*k5.*k6.*k8+k1.^2.* ...
  (egf.^2+(egf+k5).*k6).*k7.*k8).*k9).*rtot)).^(1/2))));
% second series of roots
egfbound2=(1/8).*egf.*k1.^(-1).*(egf.^2.*k3.*k4.*k5.*k6+k3.*(egf.*k4.*k5.*
...
  k6+k4.*k5.*k6.*k8+k1.^2.*(egf.^2+(egf+k5).*k6).*k7.*k8).*k9).^(-2) ...
  .*(8.*egf.^3.*k1.*k3.^2.*(k4.*k5.*k6+k1.^2.*k7.*k8.*k9).^2.*rtot+ ...
  egf.^2.*k1.*k6.*k9.*((-1).*(1+k1).^2.*k4.*k5.*k6.*k7.*k8.*(k4.*k5+ ...
  k1.^2.*k7.*k8).*k9+2.*(1+k1).^2.*(1+k2).*k3.*k4.*k5.*k7.*k8.*(k4.* ...
  k5.*k6+k1.^2.*k7.*k8.*k9)+12.*k3.^2.*(k4.*k5+k1.^2.*k7.*k8).*(k4.* ...
  k5.*k6+k1.^2.*k7.*k8.*k9).*rtot)+egf.*(4.*k1.^4.*k4.*k5.*k6.*((1+ ...
  k2).^2.*k3.^2+(-1).*k5.*k6).*k7.^2.*k8.^2.*k9.^2+k4.*k5.*k6.*((-2) ...
  .*(1+k2).*k3+k9).*(k1.^2.*k4.*k5.*k6.*k7.*k8.*k9.*((1+k1).^2.*( ...
  egf+k3+k2.*k3).^2.*k4.*k5.*k6.*k7.*k8.*k9+8.*k3.^2.*(egf.^2.*k4.* ...
  k5.*k6+(egf.*k4.*k5.*k6+k4.*k5.*k6.*k8+k1.^2.*(egf.^2+(egf+k5).* ...
  k6).*k7.*k8).*k9).*rtot)).^(1/2)+2.*k1.^5.*k6.*k7.^2.*k8.^2.* ...
  k9.^2.*((-1).*k4.*k5.^2.*k6+k3.^2.*((1+k2).^2.*k4.*k5+2.*(2.*k5+ ...
  k6).*rtot))+k1.^2.*k7.*k8.*k9.*(4.*(1+k2).^2.*k3.^2.*k4.^2.* ...
  k5.^2.*k6.^2+(-2).*(1+k2).*k3.*(k1.^2.*k4.*k5.*k6.*k7.*k8.*k9.*(( ...
```

```
1+k1).^2.*(egf+k3+k2.*k3).^2.*k4.*k5.*k6.*k7.*k8.*k9+8.*k3.^2.*( ...
egf.^2.*k4.*k5.*k6+(egf.*k4.*k5.*k6+k4.*k5.*k6.*k8+k1.^2.*(egf.^2+ ...
(egf+k5).*k6).*k7.*k8).*k9).*rtot)).^(1/2)+k6.*((-4).*k4.^2.* ...
k5.^2.*k6.*k8.*k9+(k1.^2.*k4.*k5.*k6.*k7.*k8.*k9.*((1+k1).^2.*( ...
egf+k3+k2.*k3).^2.*k4.*k5.*k6.*k7.*k8.*k9+8.*k3.^2.*(egf.^2.*k4.* ...
k5.*k6+(egf.*k4.*k5.*k6+k4.*k5.*k6.*k8+k1.^2.*(egf.^2+(egf+k5).* ...
k6).*k7.*k8).*k9).*rtot)).^(1/2)))+k1.^3.*k7.*k8.*k9.*(2.*k3.^2.* ...
k4.*k5.*k6.*((1+k2).^2.*(k4.*k5.*k6+k7.*k8.*k9)+4.*(k5.*k6+(k6+k8) ...
.*k9).*rtot)+(-2).*(1+k2).*k3.*(k1.^2.*k4.*k5.*k6.*k7.*k8.*k9.*(( ...
1+k1).^2.*(egf+k3+k2.*k3).^2.*k4.*k5.*k6.*k7.*k8.*k9+8.*k3.^2.*( ...
egf.^2.*k4.*k5.*k6+(egf.*k4.*k5.*k6+k4.*k5.*k6.*k8+k1.^2.*(egf.^2+ ...
(egf+k5).*k6).*k7.*k8).*k9).*rtot)).^(1/2)+k6.*((-2).*k4.*k5.^2.* ...
k6.*(k4+k7).*k8.*k9+(k1.^2.*k4.*k5.*k6.*k7.*k8.*k9.*((1+k1).^2.*( ...
egf+k3+k2.*k3).^2.*k4.*k5.*k6.*k7.*k8.*k9+8.*k3.^2.*(egf.^2.*k4.* ...
k5.*k6+(egf.*k4.*k5.*k6+k4.*k5.*k6.*k8+k1.^2.*(egf.^2+(egf+k5).* ...
k6).*k7.*k8).*k9).*rtot)).^(1/2)))+k1.*k4.*k5.*k6.*(2.*k3.^2.*k4.* ...
k5.*k6.*k9.*((1+k2).^2.*k7.*k8+2.*(2.*k8+k9).*rtot)+(-2).*(1+k2).* ...
k3.*(k1.^2.*k4.*k5.*k6.*k7.*k8.*k9.*((1+k1).^2.*(egf+k3+k2.*k3) ...
.^2.*k4.*k5.*k6.*k7.*k8.*k9+8.*k3.^2.*(egf.^2.*k4.*k5.*k6+(egf.* ...
k4.*k5.*k6+k4.*k5.*k6.*k8+k1.^2.*(egf.^2+(egf+k5).*k6).*k7.*k8).* ...
k9).*rtot)).^(1/2)+k9.*((-2).*k4.*k5.*k6.*k7.*k8.^2.*k9+(k1.^2.* ...
k4.*k5.*k6.*k7.*k8.*k9.*((1+k1).^2.*(egf+k3+k2.*k3).^2.*k4.*k5.* ...
k6.*k7.*k8.*k9+8.*k3.^2.*(egf.^2.*k4.*k5.*k6+(egf.*k4.*k5.*k6+k4.* ...
k5.*k6.*k8+k1.^2.*(egf.^2+(egf+k5).*k6).*k7.*k8).*k9).*rtot)).^( ...
1/2))))+k6.*k9.*(2.*k1.^4.*(1+k2).*k3.*k4.*(k3+k2.*k3+(-2).*k5).* ...
k5.*k6.*k7.^2.*k8.^2.*k9+k1.^5.*k3.*k5.*k6.*k7.^2.*k8.^2.*k9.*((1+ ...
k2).*k4.*(k3+k2.*k3+(-2).*k5)+4.*k3.*rtot)+k4.*k5.*((-1).*(1+k2).* ...
k3+2.*k8).*(k1.^2.*k4.*k5.*k6.*k7.*k8.*k9.*((1+k1).^2.*(egf+k3+ ...
k2.*k3).^2.*k4.*k5.*k6.*k7.*k8.*k9+8.*k3.^2.*(egf.^2.*k4.*k5.*k6+( ...
egf.*k4.*k5.*k6+k4.*k5.*k6.*k8+k1.^2.*(egf.^2+(egf+k5).*k6).*k7.* ...
k8).*k9).*rtot)).^(1/2)+k1.^2.*k7.*k8.*(2.*(1+k2).^2.*k3.^2.* ...
k4.^2.*k5.^2.*k6.*k9+2.*k5.*(k1.^2.*k4.*k5.*k6.*k7.*k8.*k9.*((1+ ...
k1).^2.*(egf+k3+k2.*k3).^2.*k4.*k5.*k6.*k7.*k8.*k9+8.*k3.^2.*( ...
egf.^2.*k4.*k5.*k6+(egf.*k4.*k5.*k6+k4.*k5.*k6.*k8+k1.^2.*(egf.^2+ ...
(egf+k5).*k6).*k7.*k8).*k9).*rtot)).^(1/2)+(-1).*(1+k2).*k3.*(4.* ...
k4.^2.*k5.^2.*k6.*k8.*k9+(k1.^2.*k4.*k5.*k6.*k7.*k8.*k9.*((1+k1) ...
.^2.*(egf+k3+k2.*k3).^2.*k4.*k5.*k6.*k7.*k8.*k9+8.*k3.^2.*( ...
egf.^2.*k4.*k5.*k6+(egf.*k4.*k5.*k6+k4.*k5.*k6.*k8+k1.^2.*(egf.^2+ ...
(egf+k5).*k6).*k7.*k8).*k9).*rtot)).^(1/2)))+k1.^3.*k7.*k8.*( ...
k3.^2.*k4.*k5.*k6.*k9.*((1+k2).^2.*(k4.*k5+k7.*k8)+4.*(k5+k8).* ...
rtot)+2.*k5.*(k1.^2.*k4.*k5.*k6.*k7.*k8.*k9.*((1+k1).^2.*(egf+k3+ ...
k2.*k3).^2.*k4.*k5.*k6.*k7.*k8.*k9+8.*k3.^2.*(egf.^2.*k4.*k5.*k6+( ...
egf.*k4.*k5.*k6+k4.*k5.*k6.*k8+k1.^2.*(egf.^2+(egf+k5).*k6).*k7.* ...
k8).*k9).*rtot)).^(1/2)+(-1).*(1+k2).*k3.*(2.*k4.*k5.^2.*k6.*(k4+ ...
k7).*k8.*k9+(k1.^2.*k4.*k5.*k6.*k7.*k8.*k9.*((1+k1).^2.*(egf+k3+ ...
k2.*k3).^2.*k4.*k5.*k6.*k7.*k8.*k9+8.*k3.^2.*(egf.^2.*k4.*k5.*k6+( ...
egf.*k4.*k5.*k6+k4.*k5.*k6.*k8+k1.^2.*(egf.^2+(egf+k5).*k6).*k7.* ...
k8).*k9).*rtot)).^(1/2)))+k1.*k4.*k5.*(k3.^2.*k4.*k5.*k6.*k8.*k9.* ...
((1+k2).^2.*k7+4.*rtot)+2.*k8.*(k1.^2.*k4.*k5.*k6.*k7.*k8.*k9.*(( ...
1+k1).^2.*(egf+k3+k2.*k3).^2.*k4.*k5.*k6.*k7.*k8.*k9+8.*k3.^2.*( ...
egf.^2.*k4.*k5.*k6+(egf.*k4.*k5.*k6+k4.*k5.*k6.*k8+k1.^2.*(egf.^2+ ...
(egf+k5).*k6).*k7.*k8).*k9).*rtot)).^(1/2)+(-1).*(1+k2).*k3.*(2.* ...
k4.*k5.*k6.*k7.*k8.^2.*k9+(k1.^2.*k4.*k5.*k6.*k7.*k8.*k9.*((1+k1) ...
.^2.*(egf+k3+k2.*k3).^2.*k4.*k5.*k6.*k7.*k8.*k9+8.*k3.^2.*( ...
egf.^2.*k4.*k5.*k6+(egf.*k4.*k5.*k6+k4.*k5.*k6.*k8+k1.^2.*(egf.^2+ ...
(egf+k5).*k6).*k7.*k8).*k9).*rtot)).^(1/2)))));
```

```
ci2=(1/4).*k2.*k3.^(-1).*(egf.^2.*k4.*k5.*k6+(egf.*k4.*k5.*k6+k4.*k5.* ...
  k6.*k8+k1.^2.*(egf.^2+(egf+k5).*k6).*k7.*k8).*k9).^(-1).*((-1).* ...
  k1.*(1+k1).*(egf+k3+k2.*k3).*k4.*k5.*k6.*k7.*k8.*k9+(k1.^2.*k4.* ...
  k5.*k6.*k7.*k8.*k9.*((1+k1).^2.*(egf+k3+k2.*k3).^2.*k4.*k5.*k6.* ...
  k7.*k8.*k9+8.*k3.^2.*(egf.^2.*k4.*k5.*k6+(egf.*k4.*k5.*k6+k4.*k5.* ...
  k6.*k8+k1.^2.*(egf.^2+(egf+k5).*k6).*k7.*k8).*k9).*rtot)).^(1/2)); ...



ca2=(1/4).*k1.^(-1).*k2.*k3.^(-1).*(egf.^2.*k4.*k5.*k6+(egf.*k4.*k5.* ...
  k6+k4.*k5.*k6.*k8+k1.^2.*(egf.^2+(egf+k5).*k6).*k7.*k8).*k9).^(-1) ...
  .*((-1).*k1.*(1+k1).*(egf+k3+k2.*k3).*k4.*k5.*k6.*k7.*k8.*k9+( ...
  k1.^2.*k4.*k5.*k6.*k7.*k8.*k9.*((1+k1).^2.*(egf+k3+k2.*k3).^2.* ...
  k4.*k5.*k6.*k7.*k8.*k9+8.*k3.^2.*(egf.^2.*k4.*k5.*k6+(egf.*k4.* ...
  k5.*k6+k4.*k5.*k6.*k8+k1.^2.*(egf.^2+(egf+k5).*k6).*k7.*k8).*k9).* ...
  rtot)).^(1/2));


ei2=(1/4).*k3.^(-1).*(egf.^2.*k4.*k5.*k6+(egf.*k4.*k5.*k6+k4.*k5.*k6.* ...
  k8+k1.^2.*(egf.^2+(egf+k5).*k6).*k7.*k8).*k9).^(-1).*((-1).*k1.*( ...
  1+k1).*(egf+k3+k2.*k3).*k4.*k5.*k6.*k7.*k8.*k9+(k1.^2.*k4.*k5.* ...
  k6.*k7.*k8.*k9.*((1+k1).^2.*(egf+k3+k2.*k3).^2.*k4.*k5.*k6.*k7.* ...
  k8.*k9+8.*k3.^2.*(egf.^2.*k4.*k5.*k6+(egf.*k4.*k5.*k6+k4.*k5.*k6.* ...
  k8+k1.^2.*(egf.^2+(egf+k5).*k6).*k7.*k8).*k9).*rtot)).^(1/2));


ea2=(1/4).*k1.^(-1).*k3.^(-1).*(egf.^2.*k4.*k5.*k6+(egf.*k4.*k5.*k6+ ...
  k4.*k5.*k6.*k8+k1.^2.*(egf.^2+(egf+k5).*k6).*k7.*k8).*k9).^(-1).*( ...
  (-1).*k1.*(1+k1).*(egf+k3+k2.*k3).*k4.*k5.*k6.*k7.*k8.*k9+(k1.^2.* ...
  k4.*k5.*k6.*k7.*k8.*k9.*((1+k1).^2.*(egf+k3+k2.*k3).^2.*k4.*k5.* ...
  k6.*k7.*k8.*k9+8.*k3.^2.*(egf.^2.*k4.*k5.*k6+(egf.*k4.*k5.*k6+k4.* ...
  k5.*k6.*k8+k1.^2.*(egf.^2+(egf+k5).*k6).*k7.*k8).*k9).*rtot)).^( ...
  1/2));


eil2=(1/4).*egf.*k3.^(-2).*(egf.^2.*k4.*k5.*k6+(egf.*k4.*k5.*k6+k4.* ...
  k5.*k6.*k8+k1.^2.*(egf.^2+(egf+k5).*k6).*k7.*k8).*k9).^(-1).*((-1) ...
  .*k1.*(1+k1).*(egf+k3+k2.*k3).*k4.*k5.*k6.*k7.*k8.*k9+(k1.^2.*k4.* ...
  k5.*k6.*k7.*k8.*k9.*((1+k1).^2.*(egf+k3+k2.*k3).^2.*k4.*k5.*k6.* ...
  k7.*k8.*k9+8.*k3.^2.*(egf.^2.*k4.*k5.*k6+(egf.*k4.*k5.*k6+k4.*k5.* ...
  k6.*k8+k1.^2.*(egf.^2+(egf+k5).*k6).*k7.*k8).*k9).*rtot)).^(1/2)); ...



eal2=(1/4).*egf.*k1.^(-1).*k3.^(-2).*(egf.^2.*k4.*k5.*k6+(egf.*k4.*k5.* ...
  k6+k4.*k5.*k6.*k8+k1.^2.*(egf.^2+(egf+k5).*k6).*k7.*k8).*k9).^(-1) ...
  .*((-1).*k1.*(1+k1).*(egf+k3+k2.*k3).*k4.*k5.*k6.*k7.*k8.*k9+( ...
  k1.^2.*k4.*k5.*k6.*k7.*k8.*k9.*((1+k1).^2.*(egf+k3+k2.*k3).^2.* ...
  k4.*k5.*k6.*k7.*k8.*k9+8.*k3.^2.*(egf.^2.*k4.*k5.*k6+(egf.*k4.* ...
  k5.*k6+k4.*k5.*k6.*k8+k1.^2.*(egf.^2+(egf+k5).*k6).*k7.*k8).*k9).* ...
  rtot)).^(1/2));
```

```
des2=(1/8).*k1.*k5.*k6.*k7.*k8.*k9.*(egf.^2.*k3.*k4.*k5.*k6+k3.*(egf.* ...
  k4.*k5.*k6+k4.*k5.*k6.*k8+k1.^2.*(egf.^2+(egf+k5).*k6).*k7.*k8).* ...
  k9).^(-2).*(egf.^2.*k1.*((1+k1).^2.*k4.*k5.*k6.*k7.*k8.*k9+4.* ...
  k3.^2.*(k4.*k5.*k6+k1.^2.*k7.*k8.*k9).*rtot)+egf.*(2.*k1.*k3.*k6.* ...
  k9.*((1+k1).^2.*(1+k2).*k4.*k5.*k7.*k8+2.*k3.*(k4.*k5+k1.^2.*k7.* ...
  k8).*rtot)+(-1).*(k1.^2.*k4.*k5.*k6.*k7.*k8.*k9.*((1+k1).^2.*(egf+ ...
  k3+k2.*k3).^2.*k4.*k5.*k6.*k7.*k8.*k9+8.*k3.^2.*(egf.^2.*k4.*k5.* ...
  k6+(egf.*k4.*k5.*k6+k4.*k5.*k6.*k8+k1.^2.*(egf.^2+(egf+k5).*k6).* ...
  k7.*k8).*k9).*rtot)).^(1/2)+(-1).*k1.*(k1.^2.*k4.*k5.*k6.*k7.*k8.* ...
  k9.*((1+k1).^2.*(egf+k3+k2.*k3).^2.*k4.*k5.*k6.*k7.*k8.*k9+8.* ...
  k3.^2.*(egf.^2.*k4.*k5.*k6+(egf.*k4.*k5.*k6+k4.*k5.*k6.*k8+k1.^2.* ...
  (egf.^2+(egf+k5).*k6).*k7.*k8).*k9).*rtot)).^(1/2))+k3.*(2.* ...
  k1.^2.*(1+k2).^2.*k3.*k4.*k5.*k6.*k7.*k8.*k9+k1.^3.*k3.*k5.*k6.* ...
  k7.*k8.*k9.*((1+k2).^2.*k4+4.*rtot)+k1.*k3.*k4.*k5.*k6.*k8.*k9.*(( ...
  1+k2).^2.*k7+4.*rtot)+(-1).*(1+k2).*(k1.^2.*k4.*k5.*k6.*k7.*k8.* ...
  k9.*((1+k1).^2.*(egf+k3+k2.*k3).^2.*k4.*k5.*k6.*k7.*k8.*k9+8.* ...
  k3.^2.*(egf.^2.*k4.*k5.*k6+(egf.*k4.*k5.*k6+k4.*k5.*k6.*k8+k1.^2.* ...
  (egf.^2+(egf+k5).*k6).*k7.*k8).*k9).*rtot)).^(1/2)+(-1).*k1.*(1+ ...
  k2).*(k1.^2.*k4.*k5.*k6.*k7.*k8.*k9.*((1+k1).^2.*(egf+k3+k2.*k3) ...
  .^2.*k4.*k5.*k6.*k7.*k8.*k9+8.*k3.^2.*(egf.^2.*k4.*k5.*k6+(egf.* ...
  k4.*k5.*k6+k4.*k5.*k6.*k8+k1.^2.*(egf.^2+(egf+k5).*k6).*k7.*k8).* ...
  k9).*rtot)).^(1/2)));


dea2=(1/8).*k1.^(-1).*k4.*k5.*k6.*k8.*k9.*(egf.^2.*k3.*k4.*k5.*k6+k3.*( ...
  egf.*k4.*k5.*k6+k4.*k5.*k6.*k8+k1.^2.*(egf.^2+(egf+k5).*k6).*k7.* ...
  k8).*k9).^(-2).*(egf.^2.*k1.*((1+k1).^2.*k4.*k5.*k6.*k7.*k8.*k9+ ...
  4.*k3.^2.*(k4.*k5.*k6+k1.^2.*k7.*k8.*k9).*rtot)+egf.*(2.*k1.*k3.* ...
  k6.*k9.*((1+k1).^2.*(1+k2).*k4.*k5.*k7.*k8+2.*k3.*(k4.*k5+k1.^2.* ...
  k7.*k8).*rtot)+(-1).*(k1.^2.*k4.*k5.*k6.*k7.*k8.*k9.*((1+k1).^2.*( ...
  egf+k3+k2.*k3).^2.*k4.*k5.*k6.*k7.*k8.*k9+8.*k3.^2.*(egf.^2.*k4.* ...
  k5.*k6+(egf.*k4.*k5.*k6+k4.*k5.*k6.*k8+k1.^2.*(egf.^2+(egf+k5).* ...
  k6).*k7.*k8).*k9).*rtot)).^(1/2)+(-1).*k1.*(k1.^2.*k4.*k5.*k6.* ...
  k7.*k8.*k9.*((1+k1).^2.*(egf+k3+k2.*k3).^2.*k4.*k5.*k6.*k7.*k8.* ...
  k9+8.*k3.^2.*(egf.^2.*k4.*k5.*k6+(egf.*k4.*k5.*k6+k4.*k5.*k6.*k8+ ...
  k1.^2.*(egf.^2+(egf+k5).*k6).*k7.*k8).*k9).*rtot)).^(1/2))+k3.*( ...
  2.*k1.^2.*(1+k2).^2.*k3.*k4.*k5.*k6.*k7.*k8.*k9+k1.^3.*k3.*k5.* ...
  k6.*k7.*k8.*k9.*((1+k2).^2.*k4+4.*rtot)+k1.*k3.*k4.*k5.*k6.*k8.* ...
  k9.*((1+k2).^2.*k7+4.*rtot)+(-1).*(1+k2).*(k1.^2.*k4.*k5.*k6.*k7.* ...
  k8.*k9.*((1+k1).^2.*(egf+k3+k2.*k3).^2.*k4.*k5.*k6.*k7.*k8.*k9+8.* ...
  k3.^2.*(egf.^2.*k4.*k5.*k6+(egf.*k4.*k5.*k6+k4.*k5.*k6.*k8+k1.^2.* ...
  (egf.^2+(egf+k5).*k6).*k7.*k8).*k9).*rtot)).^(1/2)+(-1).*k1.*(1+ ...
  k2).*(k1.^2.*k4.*k5.*k6.*k7.*k8.*k9.*((1+k1).^2.*(egf+k3+k2.*k3) ...
  .^2.*k4.*k5.*k6.*k7.*k8.*k9+8.*k3.^2.*(egf.^2.*k4.*k5.*k6+(egf.* ...
  k4.*k5.*k6+k4.*k5.*k6.*k8+k1.^2.*(egf.^2+(egf+k5).*k6).*k7.*k8).* ...
  k9).*rtot)).^(1/2)));


desl2=(1/8).*egf.*k1.*k6.*k7.*k8.*k9.*(egf.^2.*k3.*k4.*k5.*k6+k3.*(egf.* ...
  k4.*k5.*k6+k4.*k5.*k6.*k8+k1.^2.*(egf.^2+(egf+k5).*k6).*k7.*k8).* ...
  k9).^(-2).*(egf.^2.*k1.*((1+k1).^2.*k4.*k5.*k6.*k7.*k8.*k9+4.* ...
  k3.^2.*(k4.*k5.*k6+k1.^2.*k7.*k8.*k9).*rtot)+egf.*(2.*k1.*k3.*k6.* ...
  k9.*((1+k1).^2.*(1+k2).*k4.*k5.*k7.*k8+2.*k3.*(k4.*k5+k1.^2.*k7.* ...
  k8).*rtot)+(-1).*(k1.^2.*k4.*k5.*k6.*k7.*k8.*k9.*((1+k1).^2.*(egf+ ...
  k3+k2.*k3).^2.*k4.*k5.*k6.*k7.*k8.*k9+8.*k3.^2.*(egf.^2.*k4.*k5.* ...
  k6+(egf.*k4.*k5.*k6+k4.*k5.*k6.*k8+k1.^2.*(egf.^2+(egf+k5).*k6).* ...
```

```
  k7.*k8).*k9).*rtot)).^(1/2)+(-1).*k1.*(k1.^2.*k4.*k5.*k6.*k7.*k8.* ...
  k9.*((1+k1).^2.*(egf+k3+k2.*k3).^2.*k4.*k5.*k6.*k7.*k8.*k9+8.* ...
  k3.^2.*(egf.^2.*k4.*k5.*k6+(egf.*k4.*k5.*k6+k4.*k5.*k6.*k8+k1.^2.* ...
  (egf.^2+(egf+k5).*k6).*k7.*k8).*k9).*rtot)).^(1/2))+k3.*(2.* ...
  k1.^2.*(1+k2).^2.*k3.*k4.*k5.*k6.*k7.*k8.*k9+k1.^3.*k3.*k5.*k6.* ...
  k7.*k8.*k9.*((1+k2).^2.*k4+4.*rtot)+k1.*k3.*k4.*k5.*k6.*k8.*k9.*(( ...
  1+k2).^2.*k7+4.*rtot)+(-1).*(1+k2).*(k1.^2.*k4.*k5.*k6.*k7.*k8.* ...
  k9.*((1+k1).^2.*(egf+k3+k2.*k3).^2.*k4.*k5.*k6.*k7.*k8.*k9+8.* ...
  k3.^2.*(egf.^2.*k4.*k5.*k6+(egf.*k4.*k5.*k6+k4.*k5.*k6.*k8+k1.^2.* ...
  (egf.^2+(egf+k5).*k6).*k7.*k8).*k9).*rtot)).^(1/2)+(-1).*k1.*(1+ ...
  k2).*(k1.^2.*k4.*k5.*k6.*k7.*k8.*k9.*((1+k1).^2.*(egf+k3+k2.*k3) ...
  .^2.*k4.*k5.*k6.*k7.*k8.*k9+8.*k3.^2.*(egf.^2.*k4.*k5.*k6+(egf.* ...
  k4.*k5.*k6+k4.*k5.*k6.*k8+k1.^2.*(egf.^2+(egf+k5).*k6).*k7.*k8).* ...
  k9).*rtot)).^(1/2)));


des2l2=(1/8).*egf.^2.*k1.*k7.*k8.*k9.*(egf.^2.*k3.*k4.*k5.*k6+k3.*(egf.* ...
  k4.*k5.*k6+k4.*k5.*k6.*k8+k1.^2.*(egf.^2+(egf+k5).*k6).*k7.*k8).* ...
  k9).^(-2).*(egf.^2.*k1.*((1+k1).^2.*k4.*k5.*k6.*k7.*k8.*k9+4.* ...
  k3.^2.*(k4.*k5.*k6+k1.^2.*k7.*k8.*k9).*rtot)+egf.*(2.*k1.*k3.*k6.* ...
  k9.*((1+k1).^2.*(1+k2).*k4.*k5.*k7.*k8+2.*k3.*(k4.*k5+k1.^2.*k7.* ...
  k8).*rtot)+(-1).*(k1.^2.*k4.*k5.*k6.*k7.*k8.*k9.*((1+k1).^2.*(egf+ ...
  k3+k2.*k3).^2.*k4.*k5.*k6.*k7.*k8.*k9+8.*k3.^2.*(egf.^2.*k4.*k5.* ...
  k6+(egf.*k4.*k5.*k6+k4.*k5.*k6.*k8+k1.^2.*(egf.^2+(egf+k5).*k6).* ...
  k7.*k8).*k9).*rtot)).^(1/2)+(-1).*k1.*(k1.^2.*k4.*k5.*k6.*k7.*k8.* ...
  k9.*((1+k1).^2.*(egf+k3+k2.*k3).^2.*k4.*k5.*k6.*k7.*k8.*k9+8.* ...
  k3.^2.*(egf.^2.*k4.*k5.*k6+(egf.*k4.*k5.*k6+k4.*k5.*k6.*k8+k1.^2.* ...
  (egf.^2+(egf+k5).*k6).*k7.*k8).*k9).*rtot)).^(1/2))+k3.*(2.* ...
  k1.^2.*(1+k2).^2.*k3.*k4.*k5.*k6.*k7.*k8.*k9+k1.^3.*k3.*k5.*k6.* ...
  k7.*k8.*k9.*((1+k2).^2.*k4+4.*rtot)+k1.*k3.*k4.*k5.*k6.*k8.*k9.*(( ...
  1+k2).^2.*k7+4.*rtot)+(-1).*(1+k2).*(k1.^2.*k4.*k5.*k6.*k7.*k8.* ...
  k9.*((1+k1).^2.*(egf+k3+k2.*k3).^2.*k4.*k5.*k6.*k7.*k8.*k9+8.* ...
  k3.^2.*(egf.^2.*k4.*k5.*k6+(egf.*k4.*k5.*k6+k4.*k5.*k6.*k8+k1.^2.* ...
  (egf.^2+(egf+k5).*k6).*k7.*k8).*k9).*rtot)).^(1/2)+(-1).*k1.*(1+ ...
  k2).*(k1.^2.*k4.*k5.*k6.*k7.*k8.*k9.*((1+k1).^2.*(egf+k3+k2.*k3) ...
  .^2.*k4.*k5.*k6.*k7.*k8.*k9+8.*k3.^2.*(egf.^2.*k4.*k5.*k6+(egf.* ...
  k4.*k5.*k6+k4.*k5.*k6.*k8+k1.^2.*(egf.^2+(egf+k5).*k6).*k7.*k8).* ...
  k9).*rtot)).^(1/2)));


deal2=(1/8).*egf.*k1.^(-1).*k4.*k5.*k6.*k9.*(egf.^2.*k3.*k4.*k5.*k6+k3.* ...
  (egf.*k4.*k5.*k6+k4.*k5.*k6.*k8+k1.^2.*(egf.^2+(egf+k5).*k6).*k7.* ...
  k8).*k9).^(-2).*(egf.^2.*k1.*((1+k1).^2.*k4.*k5.*k6.*k7.*k8.*k9+ ...
  4.*k3.^2.*(k4.*k5.*k6+k1.^2.*k7.*k8.*k9).*rtot)+egf.*(2.*k1.*k3.* ...
  k6.*k9.*((1+k1).^2.*(1+k2).*k4.*k5.*k7.*k8+2.*k3.*(k4.*k5+k1.^2.* ...
  k7.*k8).*rtot)+(-1).*(k1.^2.*k4.*k5.*k6.*k7.*k8.*k9.*((1+k1).^2.*( ...
  egf+k3+k2.*k3).^2.*k4.*k5.*k6.*k7.*k8.*k9+8.*k3.^2.*(egf.^2.*k4.* ...
  k5.*k6+(egf.*k4.*k5.*k6+k4.*k5.*k6.*k8+k1.^2.*(egf.^2+(egf+k5).* ...
  k6).*k7.*k8).*k9).*rtot)).^(1/2)+(-1).*k1.*(k1.^2.*k4.*k5.*k6.* ...
  k7.*k8.*k9.*((1+k1).^2.*(egf+k3+k2.*k3).^2.*k4.*k5.*k6.*k7.*k8.* ...
  k9+8.*k3.^2.*(egf.^2.*k4.*k5.*k6+(egf.*k4.*k5.*k6+k4.*k5.*k6.*k8+ ...
  k1.^2.*(egf.^2+(egf+k5).*k6).*k7.*k8).*k9).*rtot)).^(1/2))+k3.*( ...
  2.*k1.^2.*(1+k2).^2.*k3.*k4.*k5.*k6.*k7.*k8.*k9+k1.^3.*k3.*k5.* ...
  k6.*k7.*k8.*k9.*((1+k2).^2.*k4+4.*rtot)+k1.*k3.*k4.*k5.*k6.*k8.* ...
  k9.*((1+k2).^2.*k7+4.*rtot)+(-1).*(1+k2).*(k1.^2.*k4.*k5.*k6.*k7.* ...
  k8.*k9.*((1+k1).^2.*(egf+k3+k2.*k3).^2.*k4.*k5.*k6.*k7.*k8.*k9+8.* ...
```

```
  k3.^2.*(egf.^2.*k4.*k5.*k6+(egf.*k4.*k5.*k6+k4.*k5.*k6.*k8+k1.^2.* ...
  (egf.^2+(egf+k5).*k6).*k7.*k8).*k9).*rtot)).^(1/2)+(-1).*k1.*(1+ ...
  k2).*(k1.^2.*k4.*k5.*k6.*k7.*k8.*k9.*((1+k1).^2.*(egf+k3+k2.*k3) ...
  .^2.*k4.*k5.*k6.*k7.*k8.*k9+8.*k3.^2.*(egf.^2.*k4.*k5.*k6+(egf.* ...
  k4.*k5.*k6+k4.*k5.*k6.*k8+k1.^2.*(egf.^2+(egf+k5).*k6).*k7.*k8).* ...
  k9).*rtot)).^(1/2)));


dea2l2=(1/8).*egf.^2.*k1.^(-1).*k4.*k5.*k6.*(egf.^2.*k3.*k4.*k5.*k6+k3.*( ...
  egf.*k4.*k5.*k6+k4.*k5.*k6.*k8+k1.^2.*(egf.^2+(egf+k5).*k6).*k7.* ...
  k8).*k9).^(-2).*(egf.^2.*k1.*((1+k1).^2.*k4.*k5.*k6.*k7.*k8.*k9+ ...
  4.*k3.^2.*(k4.*k5.*k6+k1.^2.*k7.*k8.*k9).*rtot)+egf.*(2.*k1.*k3.* ...
  k6.*k9.*((1+k1).^2.*(1+k2).*k4.*k5.*k7.*k8+2.*k3.*(k4.*k5+k1.^2.* ...
  k7.*k8).*rtot)+(-1).*(k1.^2.*k4.*k5.*k6.*k7.*k8.*k9.*((1+k1).^2.*( ...
  egf+k3+k2.*k3).^2.*k4.*k5.*k6.*k7.*k8.*k9+8.*k3.^2.*(egf.^2.*k4.* ...
  k5.*k6+(egf.*k4.*k5.*k6+k4.*k5.*k6.*k8+k1.^2.*(egf.^2+(egf+k5).* ...
  k6).*k7.*k8).*k9).*rtot)).^(1/2)+(-1).*k1.*(k1.^2.*k4.*k5.*k6.* ...
  k7.*k8.*k9.*((1+k1).^2.*(egf+k3+k2.*k3).^2.*k4.*k5.*k6.*k7.*k8.* ...
  k9+8.*k3.^2.*(egf.^2.*k4.*k5.*k6+(egf.*k4.*k5.*k6+k4.*k5.*k6.*k8+ ...
  k1.^2.*(egf.^2+(egf+k5).*k6).*k7.*k8).*k9).*rtot)).^(1/2))+k3.*( ...
  2.*k1.^2.*(1+k2).^2.*k3.*k4.*k5.*k6.*k7.*k8.*k9+k1.^3.*k3.*k5.* ...
  k6.*k7.*k8.*k9.*((1+k2).^2.*k4+4.*rtot)+k1.*k3.*k4.*k5.*k6.*k8.* ...
  k9.*((1+k2).^2.*k7+4.*rtot)+(-1).*(1+k2).*(k1.^2.*k4.*k5.*k6.*k7.* ...
  k8.*k9.*((1+k1).^2.*(egf+k3+k2.*k3).^2.*k4.*k5.*k6.*k7.*k8.*k9+8.* ...
  k3.^2.*(egf.^2.*k4.*k5.*k6+(egf.*k4.*k5.*k6+k4.*k5.*k6.*k8+k1.^2.* ...
  (egf.^2+(egf+k5).*k6).*k7.*k8).*k9).*rtot)).^(1/2)+(-1).*k1.*(1+ ...
  k2).*(k1.^2.*k4.*k5.*k6.*k7.*k8.*k9.*((1+k1).^2.*(egf+k3+k2.*k3) ...
  .^2.*k4.*k5.*k6.*k7.*k8.*k9+8.*k3.^2.*(egf.^2.*k4.*k5.*k6+(egf.* ...
  k4.*k5.*k6+k4.*k5.*k6.*k8+k1.^2.*(egf.^2+(egf+k5).*k6).*k7.*k8).* ...
  k9).*rtot)).^(1/2)));


numOfElements=numel([egfbound1, ci1, ca1, ei1, ea1, eil1, eal1, des1, desl1,
des2l1, dea1, deal1, dea2l1]);
if sum(sum([egfbound1, ci1, ca1, ei1, ea1, eil1, eal1, des1, desl1, des2l1,
dea1, deal1, dea2l1]>=0))==numOfElements
    boundSignal=egfbound1;
    allSpecies=[ci1, ca1, ei1, ea1, eil1, eal1, des1, desl1, des2l1, dea1,
deal1, dea2l1];
    whichRoot=1;
elseif sum(sum([egfbound2, ci2, ca2, ei2, ea2, eil2, eal2, des2, desl2,
des2l2, dea2, deal2, dea2l2]>=0))==numOfElements
    boundSignal=egfbound2;
    allSpecies=[ci2, ca2, ei2, ea2, eil2, eal2, des2, desl2, des2l2, dea2,
deal2, dea2l2];
    whichRoot=2;
else
    boundSignal=nan;
    allSpecies=nan;
    whichRoot=0;
end
```
