# Supplementary material for: Comprehensive Model for Epidermal Growth Factor Receptor Ligand Binding Involving Conformational States of the Extracellular and the Kinase Domains
Source: Front Cell Dev Biol. 2020 Aug 11;8:776. doi: 10.3389/fcell.2020.00776 (PMC7431817; doi:10.3389/fcell.2020.00776)
Supplement: Supplementary file 2 [file Data_Sheet_2.PDF]

Supplementary Material

Comprehensive model for epidermal growth factor receptor ligand binding involving conformational states of the extracellular and the kinase domains

Tímea Hajdu, Tímea Váradi, István Rebenku, Tamás Kovács, János Szöllősi, Peter Nagy

1 Supplementary figures

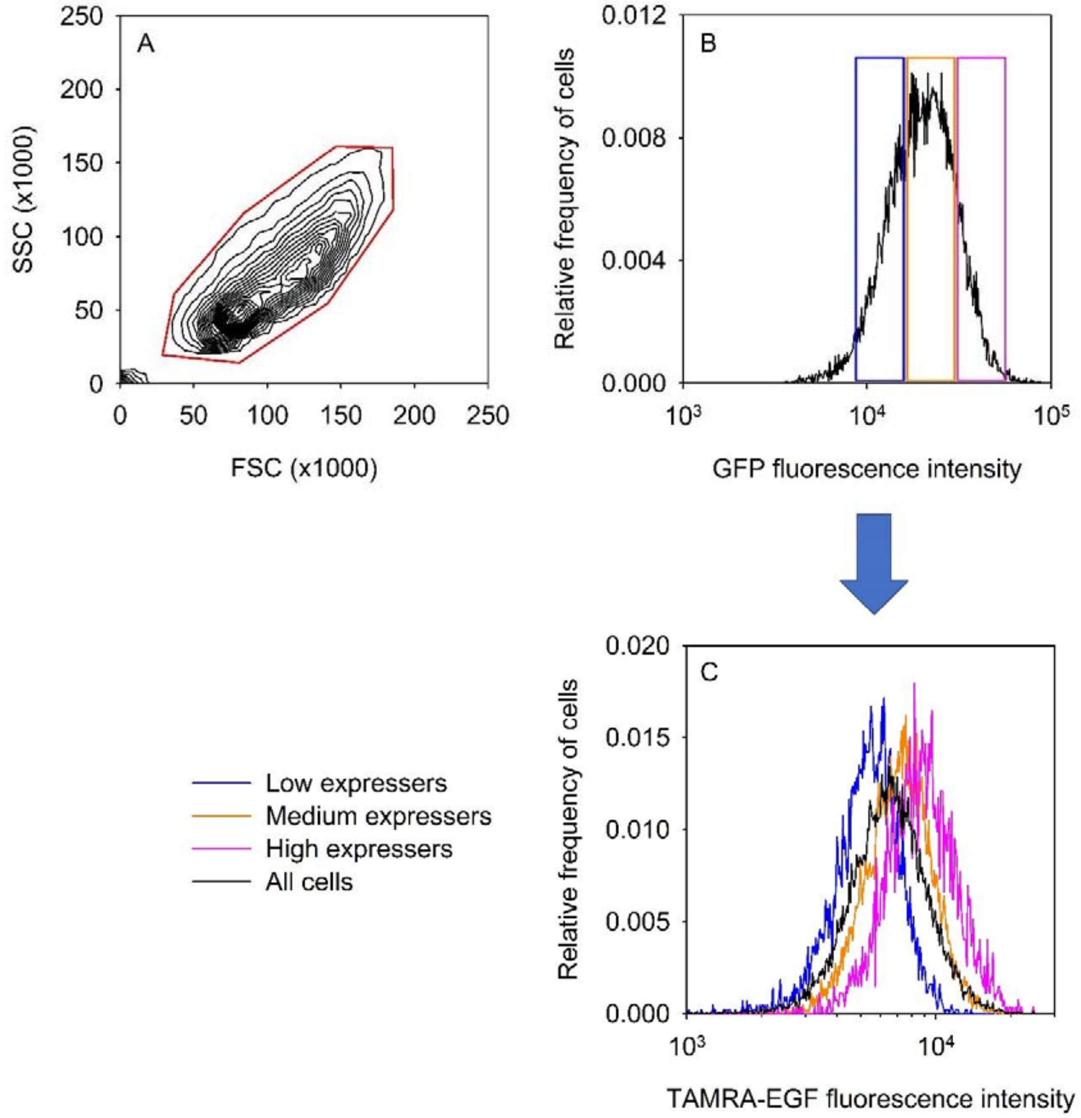

Supplementary Figure 1. Principle of selecting cell subpopulations with different levels of EGFR expression. Intact cells were selected by gating on the forward scatter (FSC) vs. side-scatter (SSC) contour plot. The selected cells are within the red polygon gate (A). The GFP fluorescence intensity histogram of these cells was further gated dividing the cells into high, medium and low expressers of EGFR-GFP (purple, orange and blue markers, respectively, panel B). The fluorescence intensity of TAMRA-EGF, representing cell-bound EGF, was separately plotted for the four cell populations followed by determining the mean of each histogram (C). The mean TAMRA-EGF fluorescence intensities were converted to the number of cell-bound EGF molecules by equating the maximum TAMRA-EGF fluorescence intensity (taken from fitting the Hill equation to the EGF concentration dependence of TAMRA-EGF fluorescence) with the number of EGFR/cell determined by flow cytometric calibration with Qifikit.

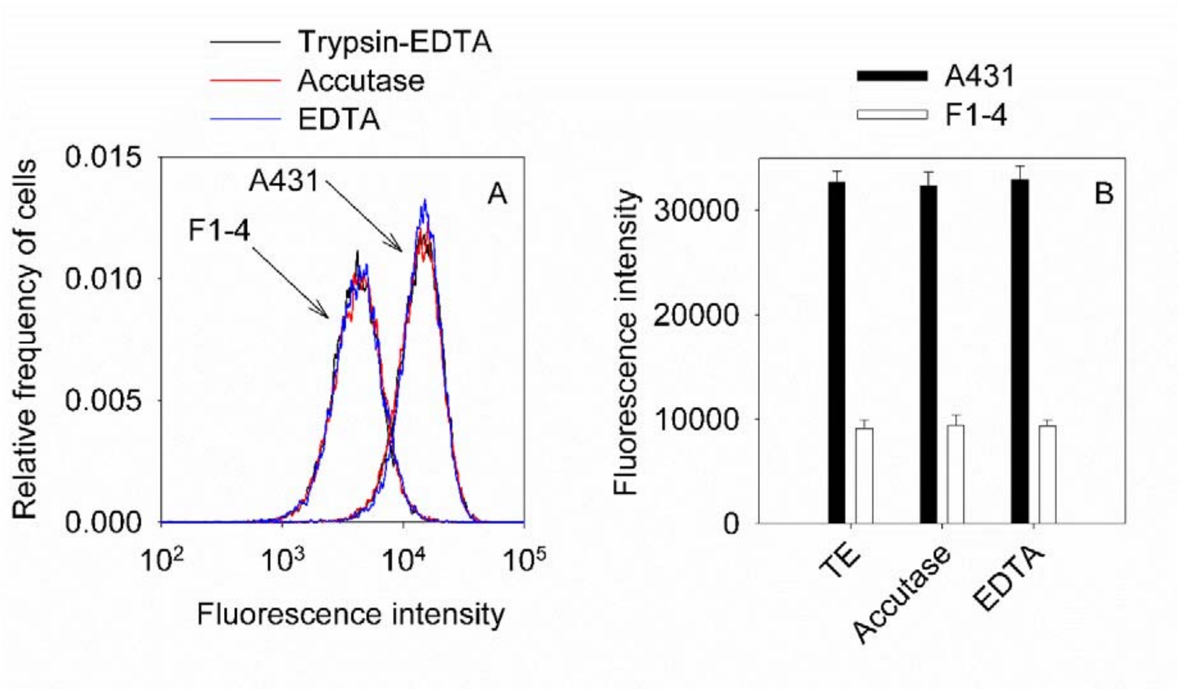

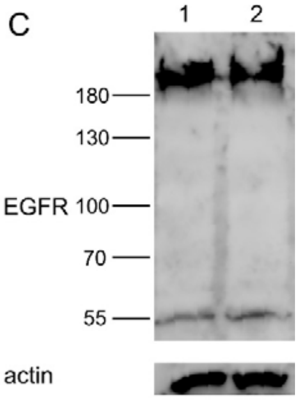

Supplementary Figure 2. Lack of significant effect of trypsinization on the expression level and molecular weight of EGFR on the cell surface. **A-B.** A431 and F1-4 cells were removed from the substrate by trypsinization ("TE", standing for trypsin-EDTA, in B), Accutase (Sigma-Aldrich, A6964) or EDTA followed by incubating cells with 20 nM TAMRA-EGF for 60 min on ice. The fluorescence intensity of cells was measured by flow cytometry. The fluorescence intensity of unlabeled cells was subtracted from the intensity of labeled samples. Representative background-corrected flow cytometric histograms (A) and the mean±standard error of the three independent measurements (B) are shown. **C.** F1-4 cells were lysed on the plate (lane 1) or they were first trypsinized followed by lysis (lane 2). Proteins were separated based on molecular weight on a 7% polyacrylamide gel followed by Western blotting. The membranes were labeled with an anti-EGFR antibody (clone F4, Sigma-Aldrich; top image) or with an anti-actin antibody (clone AC40, Sigma-Aldrich; bottom image) followed by enhanced chemiluminescence to visualize the bands. The numbers beside the blots display the molecular weight in kDa.

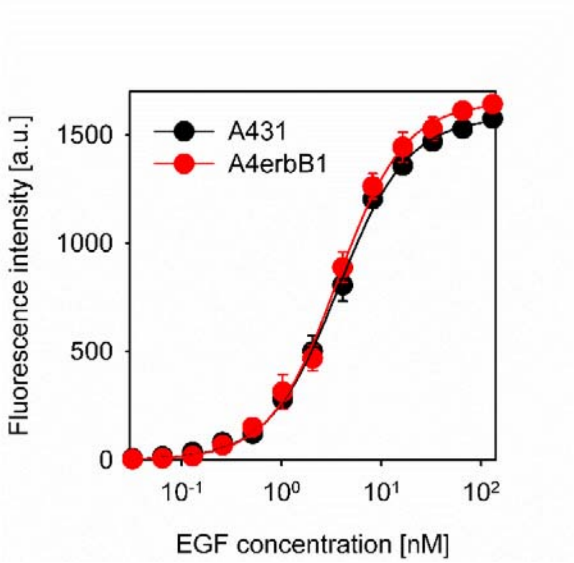

	A431	A4erbB1
K_d	3.8±0.02 nM	3.7±0.02 nM
n	1.28±0.02	1.32±0.02

Supplementary Figure 3. The apparent cooperativity and affinity of EGF binding to EGFR is not altered by GFP. A431 cells (ATCC catalog number: CRL-1555) were stably transfected with the same EGFR-GFP plasmid used throughout the experiments reported in the current manuscript. This subline of A431, named A4erbB1, was kindly provided by Donna Arndt-Jovin (Max Planck Institute for Biophysical Chemistry, Göttingen, Germany). The EGFR expression level of both cell lines was determined by flow cytometric calibration using Qifikit (Agilent Technologies, Santa Clara, CA). Although A4erbB1 expresses ~2.9 million EGFRs/cell as opposed to ~2 million EGFRs/cell on A431, gating on GFP fluorescence intensity in A4erbB1 cells, as described in Suppl. Fig. 1, was used for selecting a subpopulation whose total EGFR expression was ~2 million/cell. The ratio of endogenous, dark EGFR to EGFR-GFP in A4erbB1 cells is approximately 2:1. Both cell types were incubated with a concentration series of TAMRA-EGF for 60 min on ice followed by flow cytometry. The mean of three independent measurements (±SEM) is shown by the symbols. The Hill equation was fitted to the data (shown by the continuous lines) revealing apparent dissociations constants and cooperativities identical within experimental error for the two cell lines. If the dimerization propensity of EGFR-GFP, its ligand binding affinity or its interaction with wild-type EGFR were any different from those of native EGFR, the apparent K_d or Hill coefficient characterizing A4erbB1 cells would be different from those of A431 cells. Since the curves characterizing the two cell lines perfectly overlap each other, we conclude that the GFP tag does not significantly modify the ligand binding and dimerization properties of EGFR.

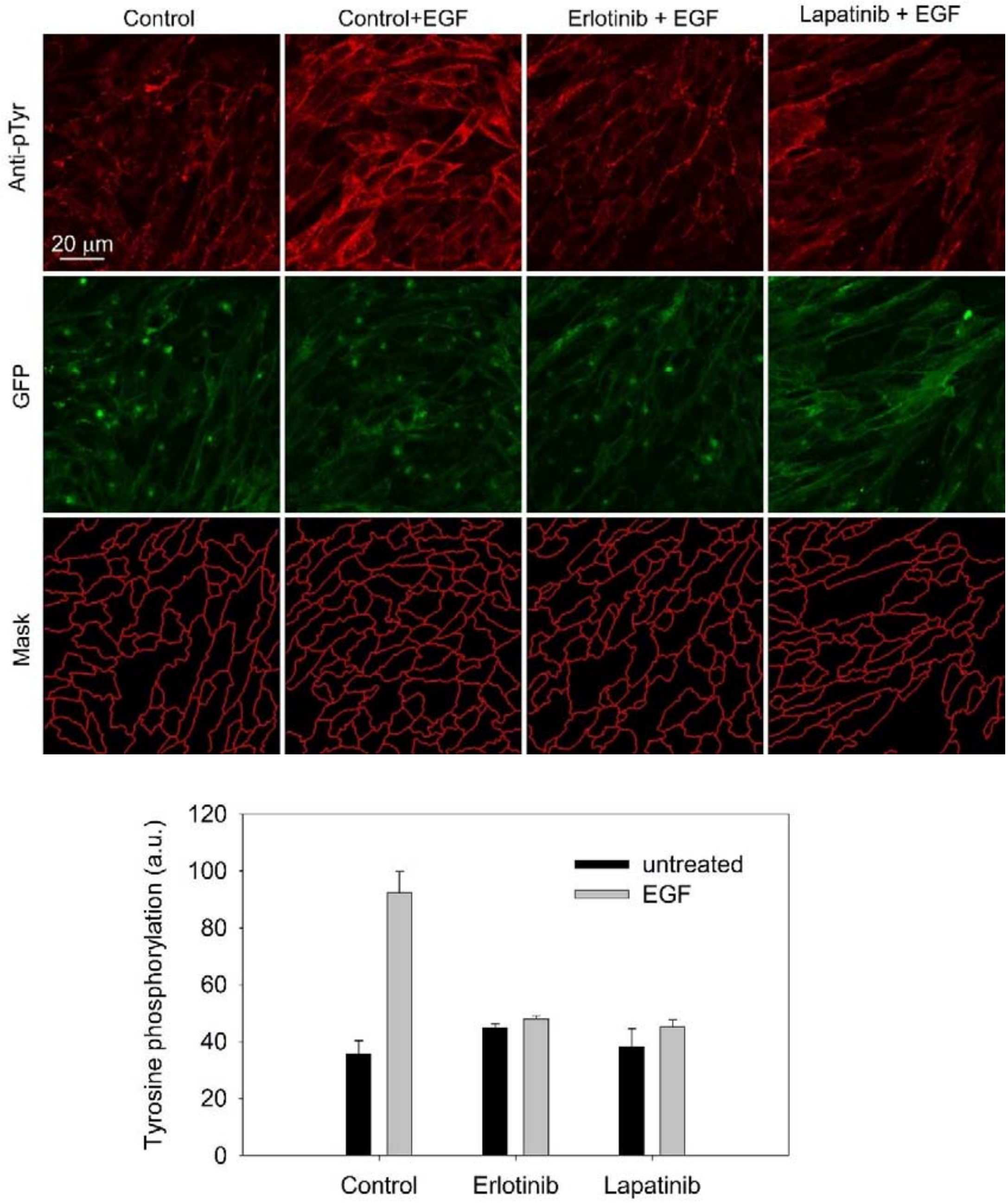

Supplementary Figure 4. Effect of kinase inhibitors on EGF-induced tyrosine phosphorylation. F1-4 cells were serum-starved overnight followed by treatment with 5 μM erlotinib or lapatinib at 37°C for 60 min. Cells were stimulated with 130 nM EGF at 37°C for 15 min followed by fixation, permeabilization and secondary staining with an anti-pTyr antibody (PY99, Santa Cruz Biotechnology) and AlexaFluor647-goat anti-mouse IgG. The GFP fluorescence corresponds to EGFR-GFP expression in the F1-4 cells. The membrane was identified with manually-seeded watershed segmentation and the intensities were analyzed only in the membrane masks. Representative images are shown in the top panel, while the evaluation of three independent measurements is displayed in the bar chart at the bottom. The error bars represent the standard error of the mean.

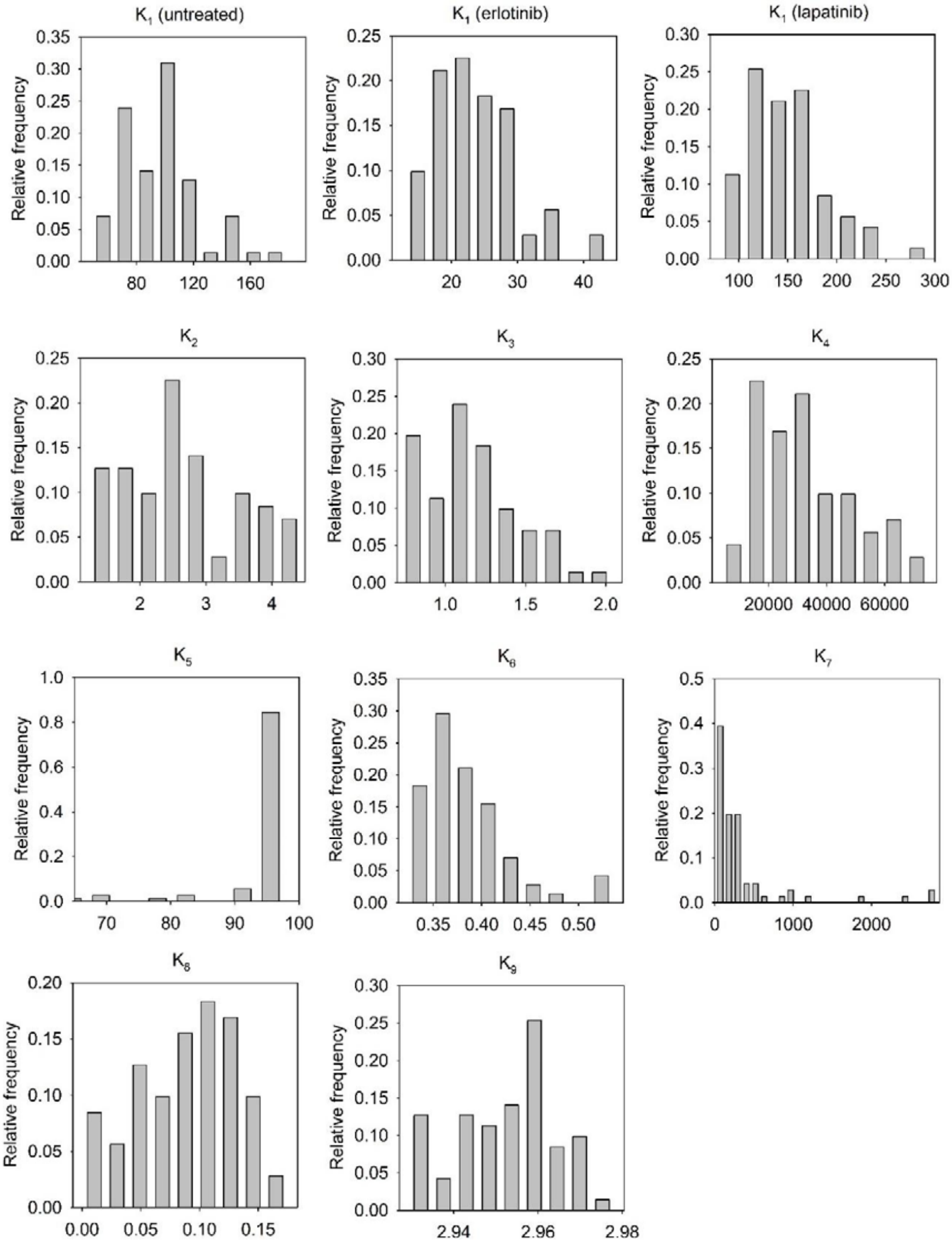

Supplementary Figure 5. Distribution of model parameters obtained from multiple fitting of the experimental results. The experimental data shown in Figure 3 of the manuscript was fitted 100-times, and the distributions of the 11 model parameters are shown by the histograms. The confidence intervals of the parameters, shown in Table 2 of the main text, were estimated from these data.

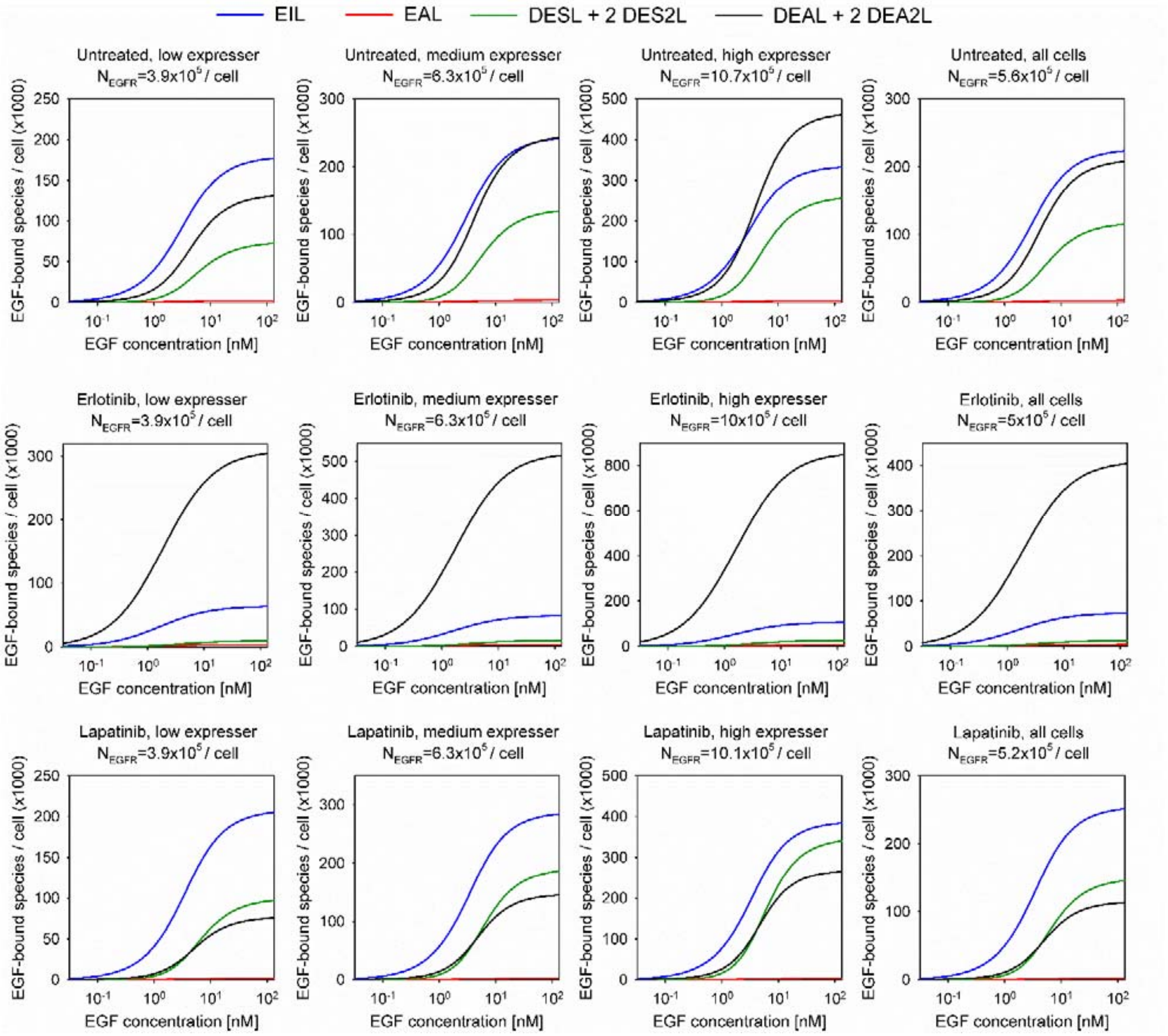

Supplementary Figure 6. Binding of EGF to different monomeric and dimeric receptor species. Equilibrium binding of EGF was calculated according to the parameters presented in Table 2 of the main text. Calculations were separately performed for control cells and cells treated with kinase inhibitors exhibiting different EGFR expressions. The number of EGF molecules bound to monomeric receptors with inactive (EIL) and active (EAL) kinase domains as well as to dimeric receptors with symmetric (DESL + 2 DES2L) and asymmetric (DEAL + 2 DEA2L) kinase domain dimers are shown in the figure. The amount of dimeric receptor species binding two EGF molecules (DES2L, DEA2L) is multiplied by two so that the displayed curves represent the amount of bound growth factor.

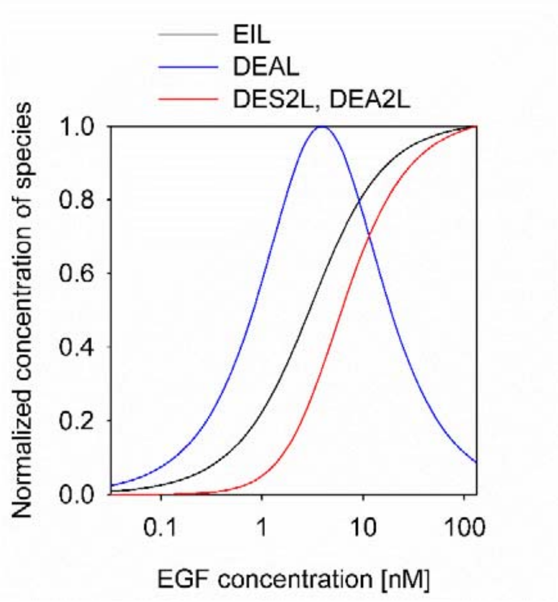

Supplementary Figure 7. Binding of EGF to different receptor species. The concentration of EGF-bound molecular species, normalized to their maximal values, were calculated according to parameters presented in Table 2 and displayed for the four species significantly binding the ligand under any of the experimental conditions (EIL –ligand-bound receptor with inactive KD; DEAL – singly liganded dimer with asymmetric KD dimer; DES2L, DEA2L – doubly-liganded dimers with symmetric and asymmetric KD dimers, respectively; the curves of these two latter species overlap). Since these normalized values do not change as a function of receptor expression or the presence or absence of an inhibitor, only the results for the low expresser subpopulation of the control sample are shown.

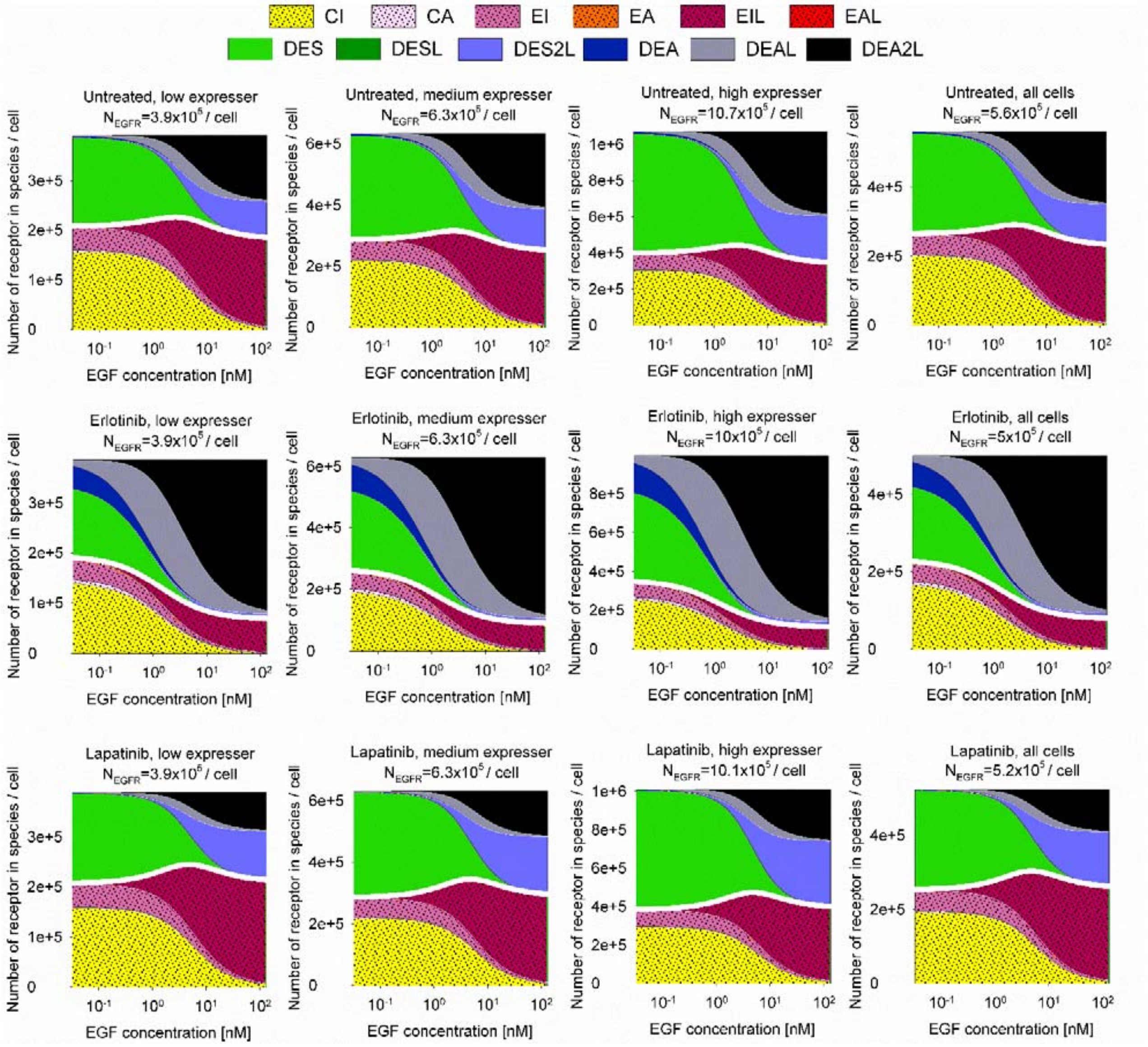

Supplementary Figure 8. EGF concentration dependent changes of monomeric and dimeric EGFR species. The amount of each receptor species is displayed in area plots as a function of EGF concentration for control and kinase inhibitor-treated cells exhibiting different receptor expression levels. The graph shows the number of receptors present in certain species, i.e. the concentration of dimeric species is multiplied by two. The thick white line separates the upper and bottom part of the plots corresponding to dimeric and monomeric receptor species, respectively. The calculation was performed according to the best fit parameters shown in Table 2 of the main text. Monomeric species whose concentrations are sufficient so that they are observable in the graphs: CI – closed ECD, inactive KD; EI – extended ECD, inactive KD; EIL – extended ECD with bound EGF, inactive KD. Monomeric species whose concentrations are too low to appear in the graphs: CA – closed ECD, active KD; EA – extended ECD, active KD; EAL – extended ECD with bound EGF, active KD. Dimeric species whose concentrations are sufficient so that they are observable in the graphs: DES – extended ECD, symmetric KD dimer; DES2L – extended ECD with two bound EGFs, symmetric KD dimer; DEA – extended ECD, asymmetric KD; DEAL - extended ECD with one bound EGF, asymmetric KD; DEA2L –

extended ECD with two bound EGFs, asymmetric KD. Dimeric species whose concentration is too low to appear in the graphs: DESL – extended ECD with one bound EGF, symmetric KD dimer.

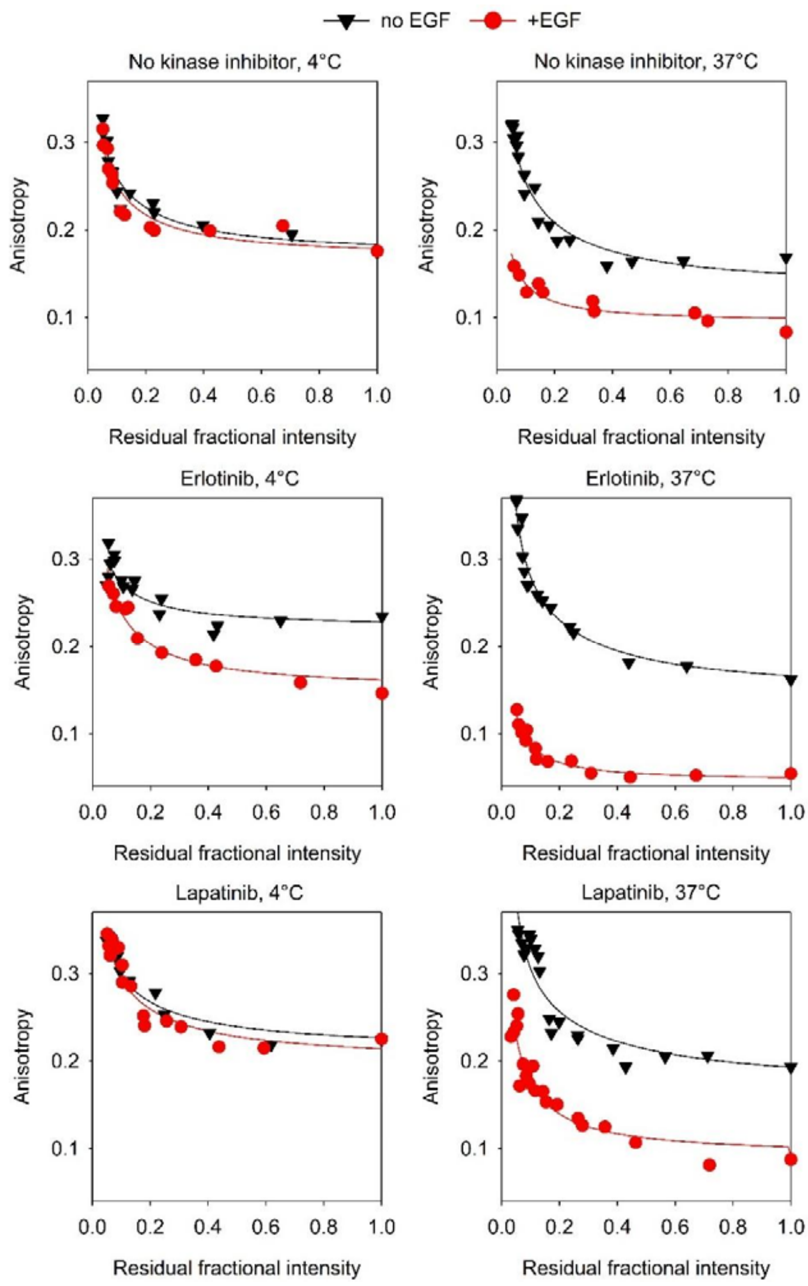

Supplementary Figure 9. Representative homo-FRET experiments of EGFR homoclustering in quiescent and growth factor-stimulated cells. F1-4 cells, stably transfected with EGFR-GFP, were serum-starved overnight. If indicated, cells were pretreated with kinase inhibitors (5 µM, 1h, 37°C) followed by stimulation with 130 nM EGF for 15 minutes at 4°C or 37°C. Anisotropy was measured by polarized detection of GFP fluorescence in unbleached cells and in cells in which GFP fluorescence was photobleached by illumination at 405 nm. The symbols represent the anisotropy values determined from confocal microscopic images plotted as a function of residual fractional GFP intensity. The lines correspond to fitting of a model describing the anisotropy of a mixture of monomeric and clustered receptors to the measured data (1).

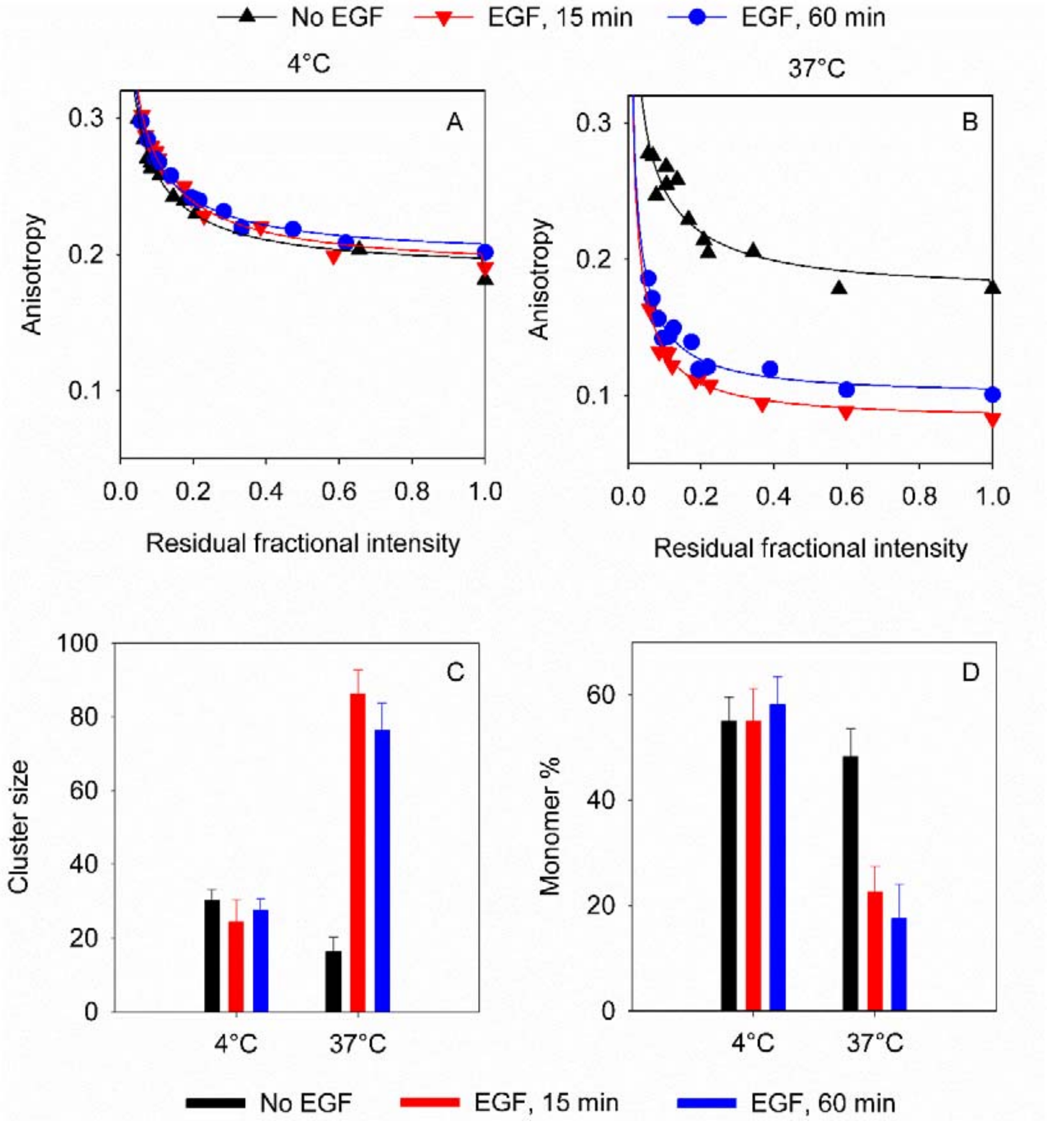

Supplementary Figure 10. Time-dependence of EGF-induced homoclustering of EGFR at 4°C and 37°C. The anisotropy of control and EGF-stimulated cells was measured by microscopy. The anisotropy was measured at different residual fractional intensities generated by bleaching of GFP. Representative data and their fits from experiments carried out at 4°C and 37°C are shown in A and B, respectively. Fitting revealed the cluster size and the monomer fraction (C, D). The error bars represent the standard error of the mean.

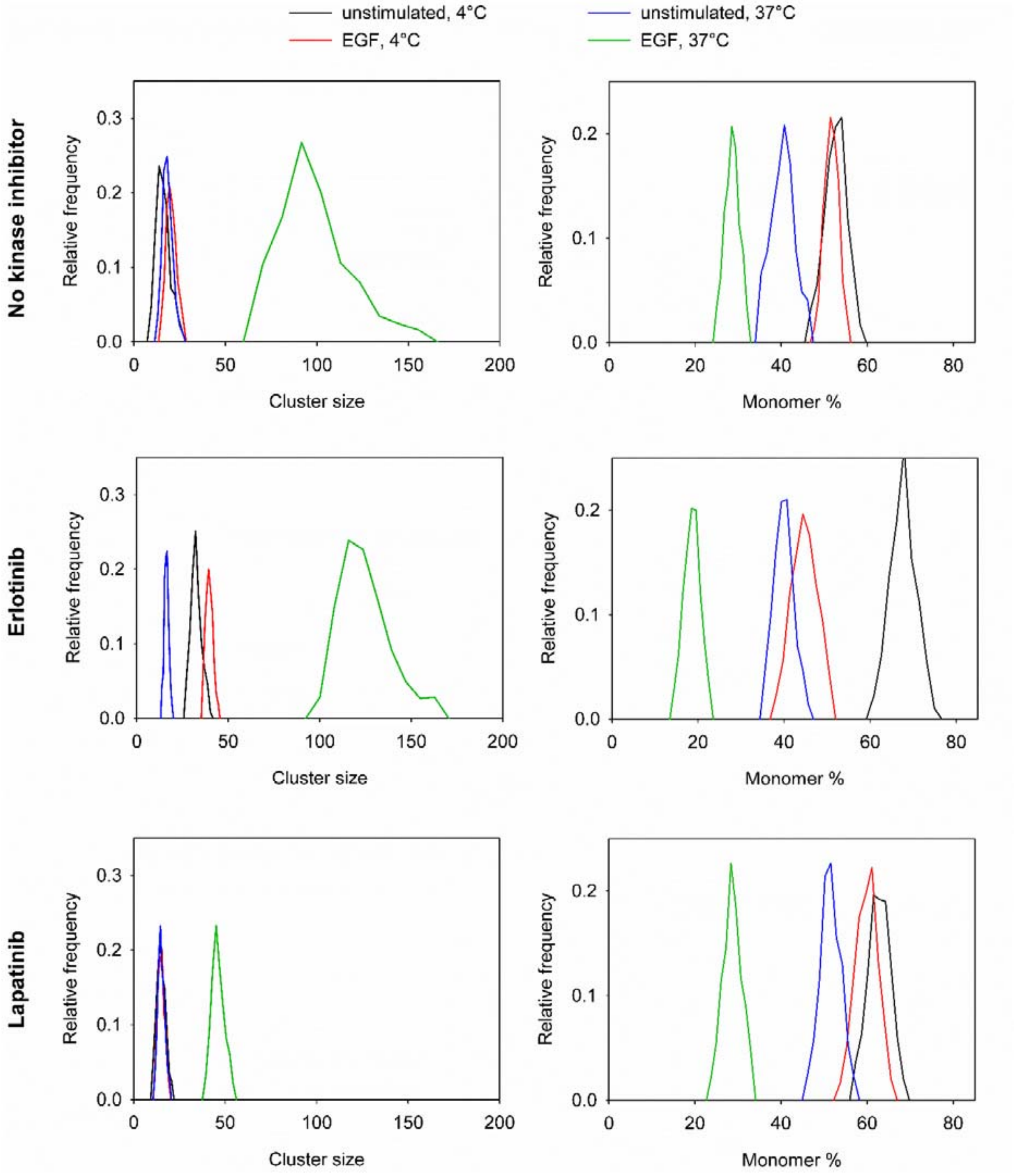

Supplementary Figure 11. Confidence intervals of cluster size and monomer percentage estimated from homo-FRET measurements. The measured anisotropy curves, shown in Suppl. Fig. 9, and the measurement errors were used for generating 500 simulated curves that were fitted resulting in 500 cluster sizes and monomer percentages. The distribution of these values, showing the reliability of the estimation, are displayed in the graphs. The number of histogram bins was determined according to Sturges' rule.

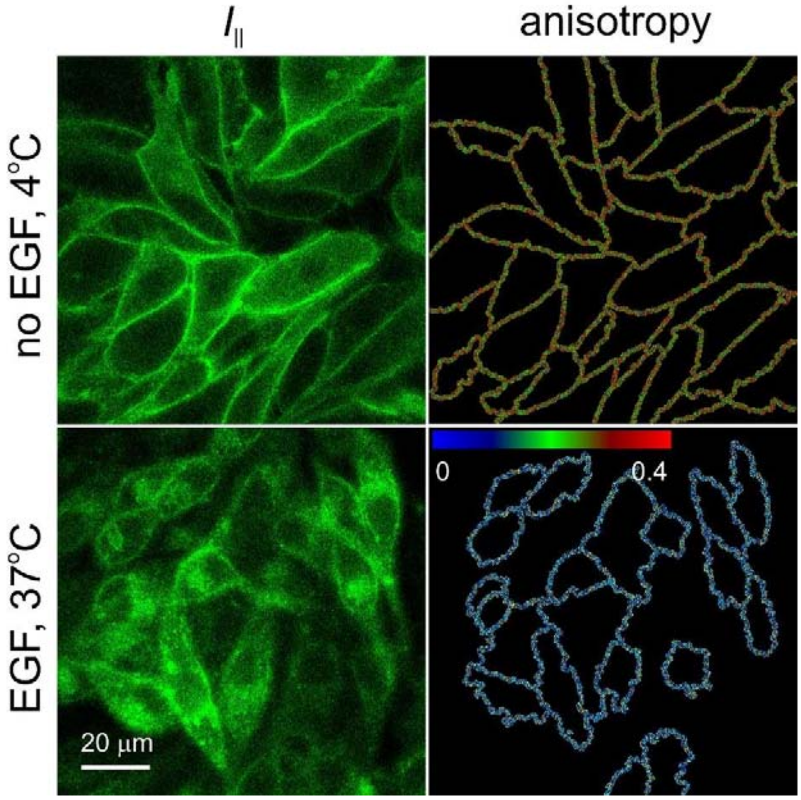

Supplementary Figure 12. Representative anisotropy images of control and EGF-stimulated cells. Control, unstimulated F1-4 cells and those incubated with 130 nM EGF at 4°C for 15 min were imaged using polarized detection of fluorescence. Since GFP was not photobleached, these images correspond to the initial condition (residual fractional intensity=1) in Suppl. Fig. 9 and 10. Fluorescence images recorded with polarizers parallel to the polarization of excitation are shown on the left. The cell membrane was identified using manually-seeded watershed segmentation followed by calculating the anisotropy in the membrane mask, which is shown in color-coded images on the right.

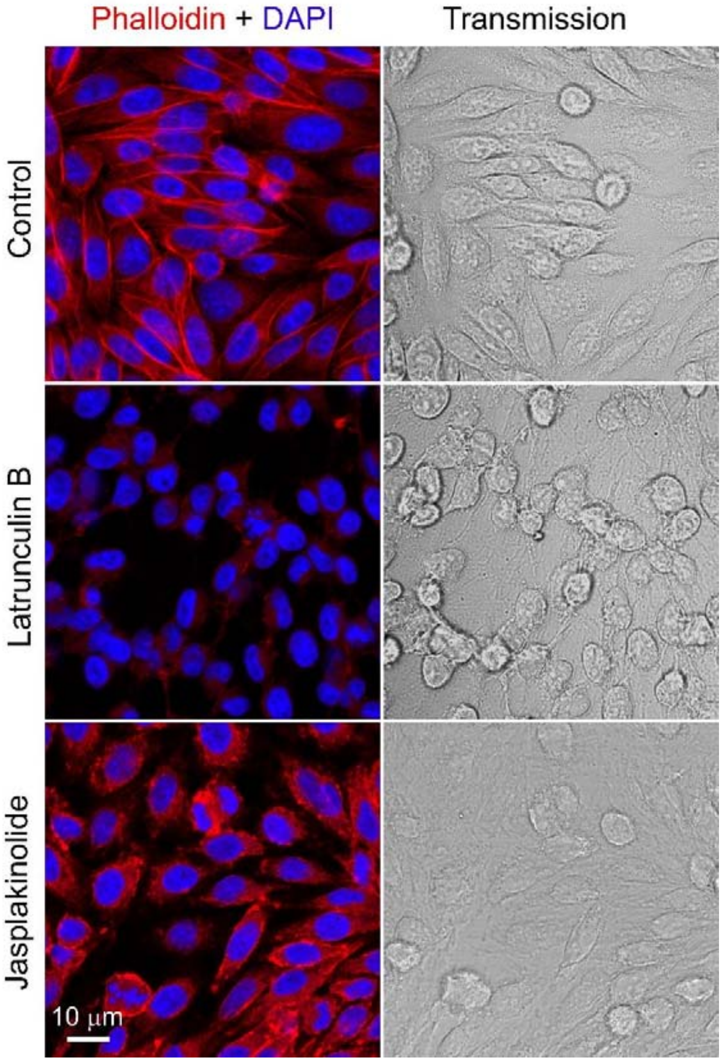

Supplementary Figure 13. Effect of latrunculin B and jasplakinolide treatment on the organization of actin filaments. Cells were treated with 2 μM latrunculin B for 10 minutes or by 1 μM jasplakinolide for 30 minutes followed by permeabilization in acetone and staining with 4 μg/ml tetramethylrhodamine-labeled phalloidin and 10 μg/ml DAPI to visualize actin filaments and the nucleus, respectively. The overlay of confocal microscopic fluorescence images and the transmission images are shown in the figure. The scale bar applies to all three conditions.

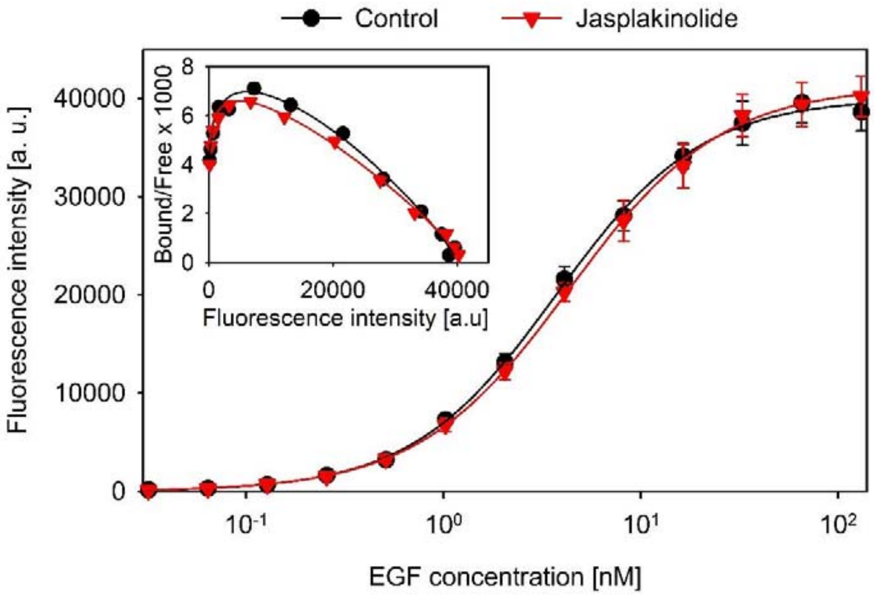

	Untreated	Jasplakinolide
K_d [nM]	3.7 ± 0.3	4.3 ± 0.3
n	1.18 ± 0.03	1.13 ± 0.05

Supplementary Figure 14. The effect of jasplakinolide on EGF binding. F1-4 cells were incubated in the presence of 1 µM jasplakinolide for 30 min at 37°C or left untreated. Cells were incubated with a concentration series of TAMRA-EGF and equilibrium binding was measured as described in Materials and Methods. The symbols display the measured data points (±SD), and the lines are fits of the Hill equation, the result of which is displayed in the table below the graph.

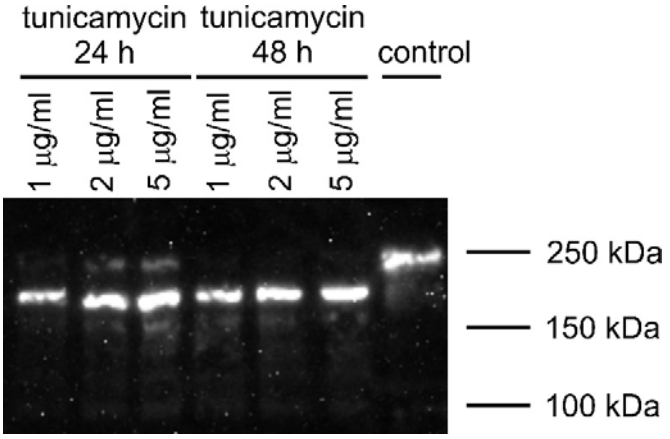

Supplementary Figure 15. Tunicamycin-induced inhibition of glycosylation shifts the molecular weight of EGFR. F1-4 cells were treated with the indicated concentrations of tunicamycin for 24 or 48 h. Proteins were separated by polyacrylamide gel electrophoresis. The EGFR on the membranes was labeled with anti-EGFR antibody (clone F4, Sigma Aldrich), and the lanes were visualized by enhanced chemiluminescence.

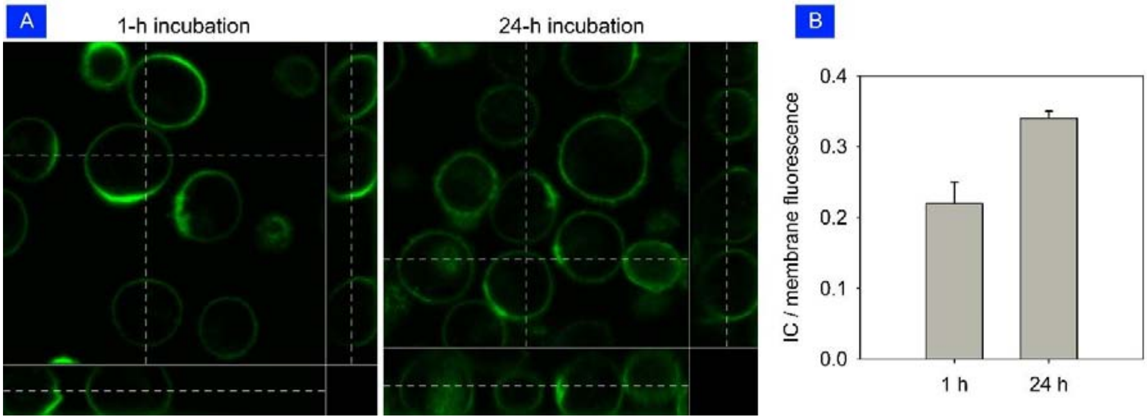

Supplementary Figure 16. Impact of 24-h incubation with fluorescent EGF on the distribution of EGF within cells. Cells were incubated with fluorescent EGF for 1 h or 24 h, and images of both samples were recorded with confocal microscopy. Orthogonal views of representative images are shown in A. Pictures were segmented with manually-seeded watershed segmentation to identify the plasma membrane. Pixels inside the membrane mask constituted the intracellular mask. The fluorescence intensity in the plasma membrane and in the intracellular space was calculated, and their ratio is plotted in part B. The difference between the ratios corresponding to the 1-h and 24-h incubations was significant (Student's t-test, $p<0.01$).

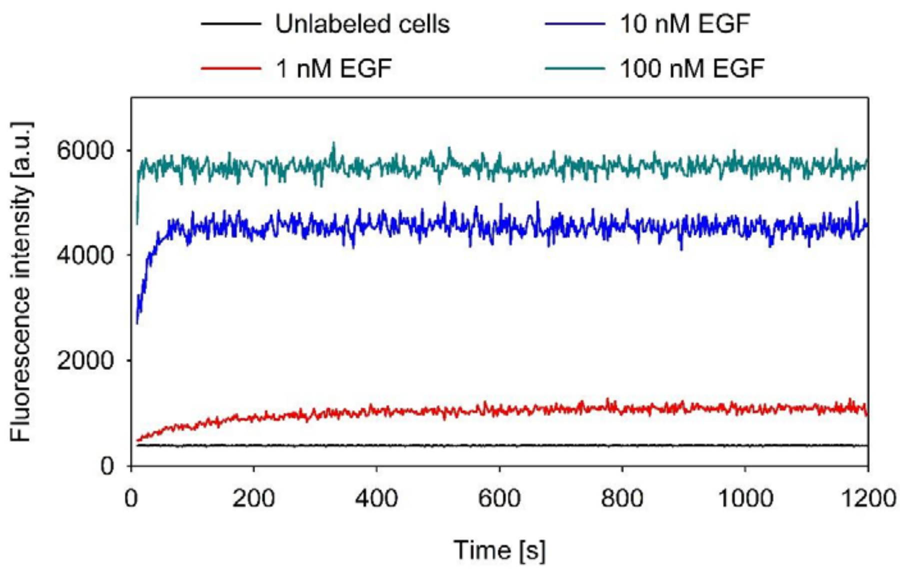

Supplementary Figure 17. Kinetics of EGF binding to cells measured by flow cytometry. Control cells or cells incubated with three different concentrations of TAMRA-EGF (1 nM, 10 nM, 100 nM) were analyzed. The sample holder of the instrument was adjusted to 4°C to mimic the conditions applied during the binding studies. Data acquisition was started immediately after adding EGF to the samples. Since the acquisition time was also saved in the list-mode files, a time-correlated analysis of cell-bound EGF could be performed. Fluorescence intensities were averaged for a sliding window width of 5 s. The experiments revealed that equilibrium is reached before 1 h, the duration of the incubation time in the equilibrium binding experiments, independent of the concentration of EGF used.

2 Supplementary theory

2.1 Estimation of ligand depletion

The total molar quantity (m_{total}) of EGF added to the incubation volume (V), in which the concentration of the growth factor was c_{EGF}, was calculated according to the following equation:

$$m_{total} = c_{EGF} V \tag{S1}$$

In order to estimate the molar quantity of bound EGF the mean intensity of each sample (I) normalized to the saturation intensity, obtained from the Hill fits ($I_{\max}$), was assumed to represent the fractional saturation (f) of EGFRs with EGF:

$$f = \frac{I}{I_{\max}} \tag{S2}$$

The molar quantity of bound EGF was calculated by multiplying the product of this fractional saturation with the number of EGFRs/cell, determined by flow cytometric calibration (N_{receptor}), and with the number of cells in the incubation volume (N_{cell}), followed by dividing with Avogadro's number (N_{A}):

$$m_{bound} = \frac{f\ N_{receptor}\ N_{cell}}{N_A} \tag{S3}$$

The percent of cell-bound growth factor, $m_{\mathrm{bound}} / m_{\mathrm{total}}$, was under 10% for all experimental conditions.

2.2 Evaluation of the negative cooperativity model about its predictions for EGFR species

The model proposed by Macdonald and Pike involves the dimerization of EGFR monomers disregarding the exteded-closed structural transition of the ECD and the conformation of the KD (2). The model is described by the following equations:

$$[M][M] = L_{20}[D] \tag{S4}$$

$$[M][EGF] = K_{11}[MEGF] \tag{S5}$$

$$[D][EGF] = K_{21}[DEGF] \tag{S6}$$

$$[DEGF][EGF] = K_{22}[D2EGF] \tag{S7}$$

$$signal = [MEGF] + [DEGF] + 2[D2EGF] \tag{S8}$$

$$[R_{tot}] = [M] + [MEGF] + 2([D] + [DEGF] + [D2EGF]) \tag{S9}$$

where M and D are the monomeric and dimeric receptor, respectively, without bound ligand, $MEGF$ is the liganded monomer, $DEGF$ and $D2EGF$ are the singly and doubly liganded receptor dimer,

respectively. L_{20} is the dissociation constant for the receptor dimer, K_{11} is the dissociation constant of the receptor monomer for EGF, and K_{21} and K_{22} are the dissociation constants for binding of the first and second ligand to the dimer, respectively. Equation (S8) describes the cell-bound signal, while equation (S9) expresses the conservation of the receptor. The Matlab function containing the solutions for all species is provided below. The function selects that root series as the meaningful solution in which all concentrations are positive.

```
function
[boundSignal,allSpecies,whichRoot]=boundEGFpike(l20,k11,k21,k22,rtot,egf)
% 1 m - monomer unliganded receptor
% 2 megf - monomer ligand-bound receptor
% 3 d - unliganded dimer
% 4 degf - dimer with one EGF
% 5 d2egf - dimer with two EGFs

signal1=(1/8).*egf.*k11.^(-2).*(egf.^2+(egf+k21).*k22).^(-2).*((-2).*(egf+ ...
  k11).*k21.^2.*k22.^2.*l20+4.*egf.*k11.^2.*(egf+k22).*(2.*egf+k22) ...
  .*rtot+k21.*k22.*((egf+k11).*(2.*egf.*k11+(-1).*egf.*k22+k11.*k22) ...
  .*l20+4.*k11.^2.*(2.*egf+k22).*rtot)+2.*egf.*k11.*((egf.*k21.* ...
  k22.*l20+k11.*k21.*k22.*l20).^2+8.*k11.^2.*k21.*k22.*(egf.^2+(egf+ ...
  k21).*k22).*l20.*rtot).^(1/2)+(-1).*egf.*k22.*((egf.*k21.*k22.* ...
  l20+k11.*k21.*k22.*l20).^2+8.*k11.^2.*k21.*k22.*(egf.^2+(egf+k21) ...
  .*k22).*l20.*rtot).^(1/2)+k11.*k22.*((egf.*k21.*k22.*l20+k11.* ...
  k21.*k22.*l20).^2+8.*k11.^2.*k21.*k22.*(egf.^2+(egf+k21).*k22).* ...
  l20.*rtot).^(1/2)+(-2).*k21.*k22.*((egf.*k21.*k22.*l20+k11.*k21.* ...
  k22.*l20).^2+8.*k11.^2.*k21.*k22.*(egf.^2+(egf+k21).*k22).*l20.* ...
  rtot).^(1/2));
m1=(-1/4).*k11.^(-1).*(egf.^2+(egf+k21).*k22).^(-1).*((egf+k11).* ...
  k21.*k22.*l20+(k21.*k22.*l20.*((egf+k11).^2.*k21.*k22.*l20+8.* ...
  k11.^2.*(egf.^2+(egf+k21).*k22).*rtot)).^(1/2));
megf1=(-1/4).*egf.*k11.^(-2).*(egf.^2+(egf+k21).*k22).^(-1).*((egf+k11) ...
  .*k21.*k22.*l20+(k21.*k22.*l20.*((egf+k11).^2.*k21.*k22.*l20+8.* ...
  k11.^2.*(egf.^2+(egf+k21).*k22).*rtot)).^(1/2));
d1=(1/8).*k11.^(-2).*k21.*k22.*(egf.^2+(egf+k21).*k22).^(-2).*(4.* ...
  egf.*k11.^2.*(egf+k22).*rtot+k21.*k22.*((egf+k11).^2.*l20+4.* ...
  k11.^2.*rtot)+egf.*((egf.*k21.*k22.*l20+k11.*k21.*k22.*l20).^2+8.* ...
  k11.^2.*k21.*k22.*(egf.^2+(egf+k21).*k22).*l20.*rtot).^(1/2)+k11.* ...
  ((egf.*k21.*k22.*l20+k11.*k21.*k22.*l20).^2+8.*k11.^2.*k21.*k22.*( ...
  egf.^2+(egf+k21).*k22).*l20.*rtot).^(1/2));
degf1=(1/8).*egf.*k11.^(-2).*k22.*(egf.^2+(egf+k21).*k22).^(-2).*(4.* ...
  egf.*k11.^2.*(egf+k22).*rtot+k21.*k22.*((egf+k11).^2.*l20+4.* ...
  k11.^2.*rtot)+egf.*((egf.*k21.*k22.*l20+k11.*k21.*k22.*l20).^2+8.* ...
  k11.^2.*k21.*k22.*(egf.^2+(egf+k21).*k22).*l20.*rtot).^(1/2)+k11.* ...
  ((egf.*k21.*k22.*l20+k11.*k21.*k22.*l20).^2+8.*k11.^2.*k21.*k22.*( ...
  egf.^2+(egf+k21).*k22).*l20.*rtot).^(1/2));
d2egf1=(1/8).*egf.^2.*k11.^(-2).*(egf.^2+(egf+k21).*k22).^(-2).*(4.*egf.* ...
  k11.^2.*(egf+k22).*rtot+k21.*k22.*((egf+k11).^2.*l20+4.*k11.^2.* ...
  rtot)+egf.*((egf.*k21.*k22.*l20+k11.*k21.*k22.*l20).^2+8.*k11.^2.* ...
  k21.*k22.*(egf.^2+(egf+k21).*k22).*l20.*rtot).^(1/2)+k11.*((egf.* ...
  k21.*k22.*l20+k11.*k21.*k22.*l20).^2+8.*k11.^2.*k21.*k22.*(egf.^2+ ...
  (egf+k21).*k22).*l20.*rtot).^(1/2));
signal2=(1/8).*egf.*k11.^(-2).*(egf.^2+(egf+k21).*k22).^(-2).*((-2).*(egf+ ...
  k11).*k21.^2.*k22.^2.*l20+4.*egf.*k11.^2.*(egf+k22).*(2.*egf+k22) ...
  .*rtot+k21.*k22.*((egf+k11).*(2.*egf.*k11+(-1).*egf.*k22+k11.*k22) ...
  .*l20+4.*k11.^2.*(2.*egf+k22).*rtot)+(-2).*egf.*k11.*((egf.*k21.* ...
```

```
  k22.*l20+k11.*k21.*k22.*l20).^2+8.*k11.^2.*k21.*k22.*(egf.^2+(egf+ ...
  k21).*k22).*l20.*rtot).^(1/2)+egf.*k22.*((egf.*k21.*k22.*l20+k11.* ...
  k21.*k22.*l20).^2+8.*k11.^2.*k21.*k22.*(egf.^2+(egf+k21).*k22).* ...
  l20.*rtot).^(1/2)+(-1).*k11.*k22.*((egf.*k21.*k22.*l20+k11.*k21.* ...
  k22.*l20).^2+8.*k11.^2.*k21.*k22.*(egf.^2+(egf+k21).*k22).*l20.* ...
  rtot).^(1/2)+2.*k21.*k22.*((egf.*k21.*k22.*l20+k11.*k21.*k22.*l20) ...
  .^2+8.*k11.^2.*k21.*k22.*(egf.^2+(egf+k21).*k22).*l20.*rtot).^( ...
  1/2));
m2=(1/4).*k11.^(-1).*(egf.^2+(egf+k21).*k22).^(-1).*(((-1).*egf+(-1) ...
  .*k11).*k21.*k22.*l20+(k21.*k22.*l20.*((egf+k11).^2.*k21.*k22.* ...
  l20+8.*k11.^2.*(egf.^2+(egf+k21).*k22).*rtot)).^(1/2));
megf2=(1/4).*egf.*k11.^(-2).*(egf.^2+(egf+k21).*k22).^(-1).*(((-1).*egf+ ...
  (-1).*k11).*k21.*k22.*l20+(k21.*k22.*l20.*((egf+k11).^2.*k21.* ...
  k22.*l20+8.*k11.^2.*(egf.^2+(egf+k21).*k22).*rtot)).^(1/2));
d2=(1/8).*k11.^(-2).*k21.*k22.*(egf.^2+(egf+k21).*k22).^(-2).*(4.* ...
  egf.*k11.^2.*(egf+k22).*rtot+k21.*k22.*((egf+k11).^2.*l20+4.* ...
  k11.^2.*rtot)+(-1).*egf.*((egf.*k21.*k22.*l20+k11.*k21.*k22.*l20) ...
  .^2+8.*k11.^2.*k21.*k22.*(egf.^2+(egf+k21).*k22).*l20.*rtot).^( ...
  1/2)+(-1).*k11.*((egf.*k21.*k22.*l20+k11.*k21.*k22.*l20).^2+8.* ...
  k11.^2.*k21.*k22.*(egf.^2+(egf+k21).*k22).*l20.*rtot).^(1/2));
degf2=(1/8).*egf.*k11.^(-2).*k22.*(egf.^2+(egf+k21).*k22).^(-2).*(4.* ...
  egf.*k11.^2.*(egf+k22).*rtot+k21.*k22.*((egf+k11).^2.*l20+4.* ...
  k11.^2.*rtot)+(-1).*egf.*((egf.*k21.*k22.*l20+k11.*k21.*k22.*l20) ...
  .^2+8.*k11.^2.*k21.*k22.*(egf.^2+(egf+k21).*k22).*l20.*rtot).^( ...
  1/2)+(-1).*k11.*((egf.*k21.*k22.*l20+k11.*k21.*k22.*l20).^2+8.* ...
  k11.^2.*k21.*k22.*(egf.^2+(egf+k21).*k22).*l20.*rtot).^(1/2));
d2egf2=(1/8).*egf.^2.*k11.^(-2).*(egf.^2+(egf+k21).*k22).^(-2).*(4.*egf.* ...
  k11.^2.*(egf+k22).*rtot+k21.*k22.*((egf+k11).^2.*l20+4.*k11.^2.* ...
  rtot)+(-1).*egf.*((egf.*k21.*k22.*l20+k11.*k21.*k22.*l20).^2+8.* ...
  k11.^2.*k21.*k22.*(egf.^2+(egf+k21).*k22).*l20.*rtot).^(1/2)+(-1) ...
  .*k11.*((egf.*k21.*k22.*l20+k11.*k21.*k22.*l20).^2+8.*k11.^2.* ...
  k21.*k22.*(egf.^2+(egf+k21).*k22).*l20.*rtot).^(1/2));

numOfElements=numel([signal1,m1,megf1,d1,degf1,d2egf1]);
if sum(sum([signal1,m1,megf1,d1,degf1,d2egf1]>=0))==numOfElements
    boundSignal=signal1;
    allSpecies=[m1,megf1,d1,degf1,d2egf1];
    whichRoot=1;
elseif sum(sum([signal2,m2,megf2,d2,degf2,d2egf2]>=0))==numOfElements
    boundSignal=signal2;
    allSpecies=[m2,megf2,d2,degf2,d2egf2];
    whichRoot=2;
else
    boundSignal=nan;
    allSpecies=nan;
    whichRoot=0;
end
```

While association constants were used in the original paper, dissociation constants are used in the above derivation in order to maintain compatibility with our model described in the main text. Taking this fact into consideration the model parameters are the following: K_{11}=0.2174 nM, K_{21}=0.1887 nM, K_{22}=2.94 nM, $L_{20}=5\cdot10^4$ receptors/cell. Using these constants and an expression level of $R_{tot}=5.6\cdot10^5$

receptors/cell, corresponding to the F1-4 cells used in the current manuscript, predictions for the different molecular species are shown in the figure below:

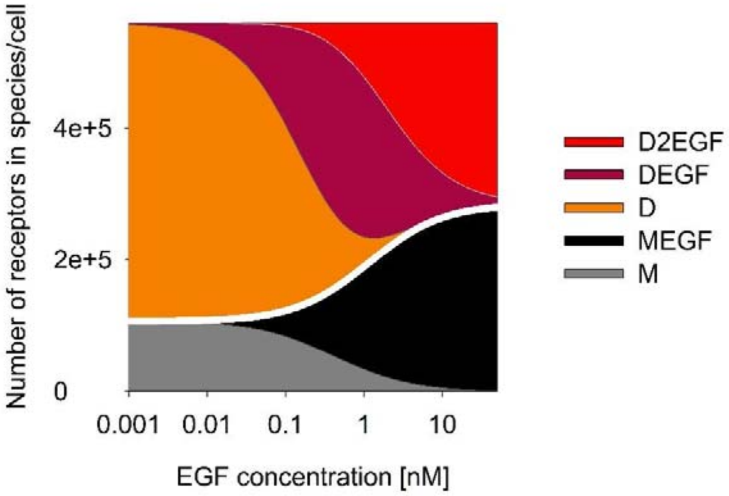

The thick white line separates monomeric and dimeric receptor species from each other. According to the model the overwhelming majority of receptors are present in preformed, constitutive dimers at an expression level of $5 \cdot 10^5$/cell. The graph also predicts that EGF induces a decline in the number of receptors in dimers. This unexpected conclusion, which is the consequence of the significantly lower homodimerization tendency of liganded monomers as compared to unliganded ones, follows from the parameters of the model, and it has been pointed out by the authors as well.

2.3 Analysis of the kinetics of reaching the equilibrium by a receptor dimer

We aimed at characterizing what kind of effect the lack of reaching an equilibrium exerts on the apparent cooperativity of EGF binding. First, we set up a system of differential equations describing the binding kinetics of the ligand:

$$\frac{dR0(t)}{dt} = -2k_{1,\mathrm{ON}}\ R0(t)\ c_{EGF} + k_{1,\mathrm{OFF}}\ R1(t) \tag{S10}$$

$$\frac{dR1(t)}{dt} = 2k_{1,\mathrm{ON}}\ R0(t)\ c_{EGF} - k_{1,\mathrm{OFF}}\ R1(t) - k_{2,\mathrm{ON}}\ R1(t)\ c_{EGF} + 2k_{2,\mathrm{OFF}}\ R2(t) \tag{S11}$$

$$\frac{dR2(t)}{dt} = k_{2,\mathrm{ON}}\ R1(t)\ c_{EGF} - 2k_{2,\mathrm{OFF}}\ R2(t) \tag{S12}$$

where $R0$, $R1$ and $R2$ are concentrations of the unoccupied, singly and doubly liganded dimers, respectively, c_{EGF} is the concentration of EGF in the solution. $k_{1,\mathrm{ON}}$ and $k_{1,\mathrm{OFF}}$ are the association and dissociation rate constants of EGF for the first binding site, and $k_{2,\mathrm{ON}}$ and $k_{2,\mathrm{OFF}}$ are the same parameters for the second binding site. Multiplication with two of certain terms on the right-hand side of the equations takes the fact into account that certain transitions can take place via two, kinetically different

ways, e.g. the *R0*→*R1* step can be accomplished by the ligand associating with either one of the receptor monomers:

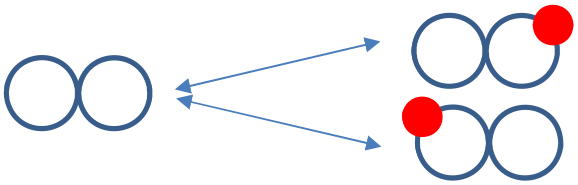

The above differential equation system can be rewritten as a matrix differential equation as follows:

$$\begin{pmatrix} R0'(t) \\ R1'(t) \\ R2'(t) \end{pmatrix} = \mathbf{A} \begin{pmatrix} R0(t) \\ R1(t) \\ R2(t) \end{pmatrix} \tag{S13}$$

$$\mathbf{A} = \begin{pmatrix} -2k_{1,\mathrm{ON}}c_{EGF} & k_{1,\mathrm{OFF}} & 0 \\ 2k_{1,\mathrm{ON}}c_{EGF} & -k_{1,\mathrm{OFF}} - k_{2,\mathrm{ON}}c_{EGF} & 2k_{2,\mathrm{OFF}} \\ 0 & k_{2,\mathrm{ON}}c_{EGF} & -2k_{2,\mathrm{OFF}} \end{pmatrix}$$

The general solution of the equation above is given below:

$$\begin{pmatrix} R0(t) \\ R1(t) \\ R2(t) \end{pmatrix} = C_1 e^{-\lambda_1 t}\mathbf{v}_1 + C_2 e^{-\lambda_2 t}\mathbf{v}_2 + C_3 e^{-\lambda_3 t}\mathbf{v}_3 \tag{S14}$$

where C_1, C_2 and C_3 are the eigenvalues of matrix **A**, and $\mathbf{v}_1$, $\mathbf{v}_2$ and $\mathbf{v}_3$ are the eigenvectors of matrix **A**. The eigenvalues and eigenvectors were found using Mathematica. The specific solution corresponding to the current problem was found by setting the initial concentrations as follows:

$$R0(t) = R_{tot},\ R1(t) = R2(t) = 0 \tag{S15}$$

For reasons described above the dissociation constants of EGF for the 1st and 2nd binding sites are given by the following equations:

$$K_{d,1} = \frac{k_{1,\mathrm{OFF}}}{2k_{1,\mathrm{ON}}},\ K_{d,2} = \frac{2k_{2,\mathrm{OFF}}}{k_{2,\mathrm{ON}}} \tag{S16}$$

In order to reveal the effect of the lack of reaching equilibrium binding conditions, the following, arbitrary dissociation constants generating negative cooperativity were used: K_{d1}=3 nM, K_{d2}=12 nM. Assuming $k_{1,\mathrm{ON}}$= $k_{2,\mathrm{ON}}$=1, the dissociation rate constants were calculated and substituted into the solution of the differential equation system. The kinetics of ligand binding were calculated at an EGF concentration of 10 nM (panel A). Next, we analyzed the dependence of cell-bound EGF at two time points corresponding to before (t=0.1 s) and after (t=10 s) reaching the equilibrium. The Scatchard plots in panel B demonstrate that the concave-up plot corresponding to t=10 s, indicative of negative

cooperativity, is converted to a concave-down curve at t=0.1 s implying positive cooperativity. Fitting of the binding curves with the Hill equation confirmed the conclusions reached based on the Scatchard plots (K_d=8.2 nM, n=0.88 at t=10 s; K_d=11 nM, n=1.1 at t=0.1 s).

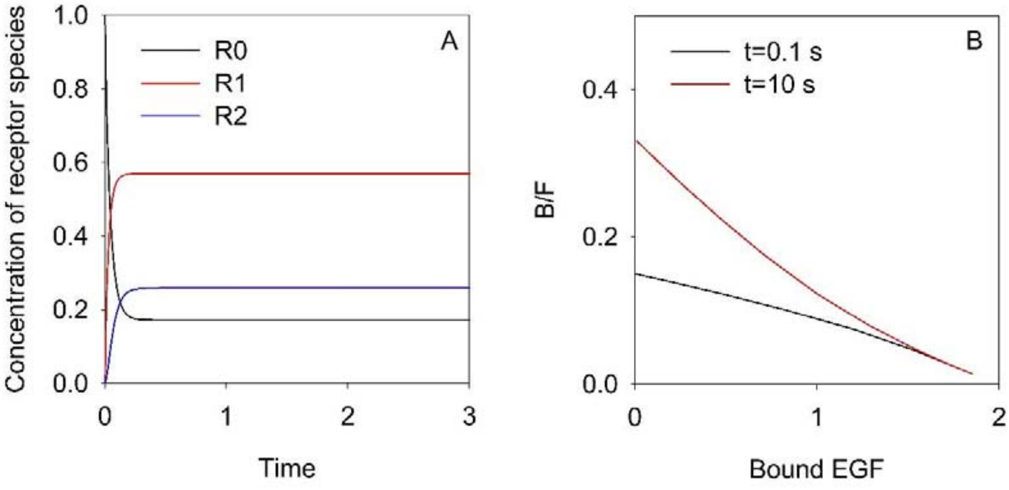

3 References

1. Szabó A, Horváth G, Szöllősi J, Nagy P. Quantitative characterization of the large-scale association of ErbB1 and ErbB2 by flow cytometric homo-FRET measurements. Biophys J. 2008;95(4):2086-96.

2. Macdonald JL, Pike LJ. Heterogeneity in EGF-binding affinities arises from negative cooperativity in an aggregating system. Proc Natl Acad Sci U S A. 2008;105(1):112-7.
